# Evaluation of *Ogataea* (*Hansenula*) *polymorpha* for Hyaluronic Acid Production

**DOI:** 10.3390/microorganisms9020312

**Published:** 2021-02-03

**Authors:** João Heitor Colombelli Manfrão-Netto, Enzo Bento Queiroz, Kelly Assis Rodrigues, Cintia M. Coelho, Hugo Costa Paes, Elibio Leopoldo Rech, Nádia Skorupa Parachin

**Affiliations:** 1 Grupo Engenharia de Biocatalisadores, Instituto de Ciências Biológicas, Universidade de Brasília, Brasília 70910-900, Brazil; jhnettobio@gmail.com (J.H.C.M.-N.); enzo.beno@gmail.com (E.B.Q.); rodrigues.bio23@gmail.com (K.A.R.); 2 Department of Genetics and Morphology, Institute of Biological Science, University of Brasília, Brasília 70910-900, Brazil; cintia.coelhom@gmail.com; 3 Clinical Medicine Division, University of Brasília Medical School, University of Brasília, Brasília 70910-900, Brazil; sorumbatico@gmail.com; 4 Brazilian Agriculture Research Corporation—Embrapa—Genetic Resources and Biotechnology—CENARGEN, Brasília 70770-917, Brazil; elibio.rech@embrapa.br; 5 Ginkgo Bioworks, Boston, MA 02210, USA

**Keywords:** *Ogataea polymorpha* 1, hyaluronic acid 2, methylotrophic yeast 3, genomic editing 4, genetic switch 5, promoters 6

## Abstract

Hyaluronic acid (HA) is a biopolymer formed by UDP-glucuronic acid and UDP-N-acetyl-glucosamine disaccharide units linked by β-1,4 and β-1,3 glycosidic bonds. It is widely employed in medical and cosmetic procedures. HA is synthesized by hyaluronan synthase (HAS), which catalyzes the precursors’ ligation in the cytosol, elongates the polymer chain, and exports it to the extracellular space. Here, we engineer *Ogataea* (*Hansenula*) *polymorpha* for HA production by inserting the genes encoding UDP-glucose 6-dehydrogenase, for UDP-glucuronic acid production, and HAS. Two microbial HAS, from *Streptococcus zooepidemicus* (*hasAs*) and *Pasteurella multocida* (*hasAp*), were evaluated separately. Additionally, we assessed a genetic switch using integrases in *O. polymorpha* to uncouple HA production from growth. Four strains were constructed containing both *has* genes under the control of different promoters. In the strain containing the genetic switch, HA production was verified by a capsule-like layer around the cells by scanning electron microscopy in the first 24 h of cultivation. For the other strains, the HA was quantified only after 48 h and in an optimized medium, indicating that HA production in *O. polymorpha* is limited by cultivation conditions. Nevertheless, these results provide a proof-of-principle that *O. polymorpha* is a suitable host for HA production.

## 1. Introduction

Microorganisms have been widely used to obtain pharmaceuticals, biopolymers, vaccines, enzymes, and various other chemicals [1]. Through metabolic engineering and synthetic biology approaches, it is possible to generate recombinant strains to produce desired compounds and bypass the chemical synthesis [2]. One of the value-added products that can be obtained through microorganism-based processes is hyaluronic acid (HA). This biopolymer features high viscosity and elasticity and is abundant in the extracellular matrix of vertebrates’ connective tissues. Due to its high biocompatibility, HA has various applications in the medical, cosmetic, and pharmaceutical areas (see details in [3]). According to Grand View Research Inc., the global HA market may reach USD 16.6 billion by 2027 [4].

**Figure 1:**
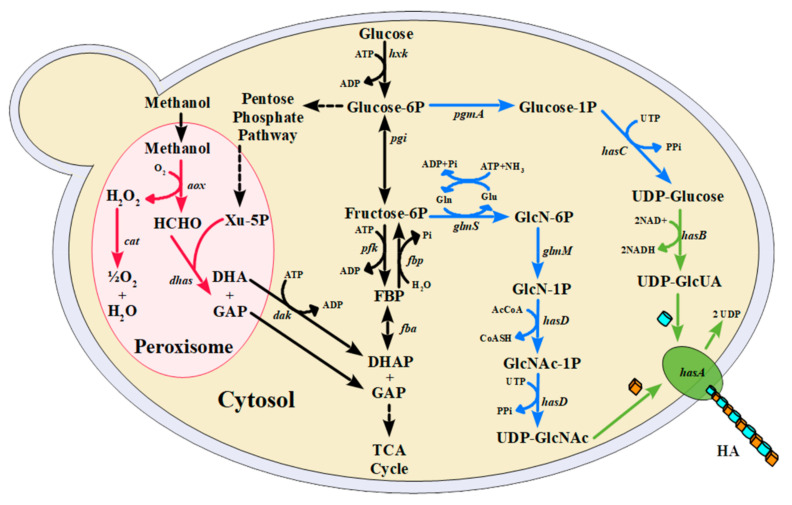
Biosynthetic pathway for hyaluronic acid (HA) production in *Ogataea* (*Hansenula*) *polymorpha*. The black and blue arrows indicate the endogenous glycolysis and HA precursor pathways, respectively. The green arrows indicate the exogenous pathway inserted in *O. polymorpha* for HA production. The red arrows represent methanol metabolism. Genes encoding enzymes of the pathways are shown: *aox*: alcohol oxidase; *cat*: catalase; *dhas*: dihydroxyacetone synthase; *dak*: dihydroxyacetone kinase; *hxk*: hexokinase; *pgi*: glucose-6-phosphate isomerase; *pfk*: phosphofructokinase; *fbp*: fructose-1, 6-bisphosphatase; *fba*: fructose-bisphosphate aldolase; *pgmA*: phosphoglucomutase; *hasC*: UDP-glucose pyrophosphorylase; *hasB*: UDP-glucose dehydrogenase; *glmS*: amidotransferase; *glmM*: phosphoglucosamine mutase; *hasD*: acetyltransferase and UDP-N-acetyl-glucosamine pyrophosphorylase; *hasA*: hyaluronic acid synthase. Molecules: DHA: dihydroxyacetone; DHAP: dihydroxyacetone phosphate; GAP: glyceraldehyde-3-phosphate; Xu-5P: xylulose-5-phosphate; FBP: fructose-1, 6-bisphosphate; Glucose-6P: glucose-6-phosphate; Fructose-6P: fructose-6-phosphate; Glucose-1P: glucose-1-phosphate; UDP-glcUA: UDP-glucuronic acid; GlcN-6P: glucosamine-6-phosphate; GlcN-1P: glucosamine-1-phosphate; GlcNAc-1P: N-acetyl-glucosamine-1-phosphate; UDP-GlcNAc: UDP-N-acetyl-glucosamine; HA: hyaluronic acid.The methylotrophic yeast *O. polymorpha* (formerly known as *Hansenula polymorpha* or *Pichia angusta*) is used as an expression platform because of its features, which include fermentation at high cell densities, low-cost substrate utilization, defined synthetic medium, secretion of heterologous proteins in high levels, established strategies for bioreactor cultivation, and availability of synthetic biology tools for genetic manipulation [13]. *O. polymorpha* is widely used to produce biopharmaceuticals, including Insulin (Wosulin^®^), α-Interferon (Reiferon^®^), and others [14], and therefore it is an attractive host platform to produce HA. Furthermore, this non-conventional yeast is thermotolerant, and this can be explored for HA production, as it was recently reported that temperature changes affect the chain length of HA as well as the titers [15]. In *B. subtilis*, an increase in the cultivation temperature led to an increase in HA’s molecular weight but a decrease in its concentration in the culture broth.

HA is a glycosaminoglycan formed by disaccharide repeats of UDP-glucuronic acid (UDP-GlcUA) and UDP-N-acetylglucosamine (UDP-GlcNAc) linked by β-1,4 and β-1,3 glycosidic bonds [5]. Its synthesis is catalyzed by the enzyme hyaluronan synthase (HAS), encoded by the gene *hasA*, and responsible for assembling the two precursors in the cytosol, elongating the polymer chain and releasing it into the extracellular matrix. Gram-positive bacteria and yeasts are the most used microorganisms for producing heterologous HA, especially those with Generally Recognized as Safe (GRAS) status. Many such strains have been developed in recent years, using different approaches to improve HA titers [6]. *Bacillus subtilis* [7,8], *Corynebacterium glutamicum* [9], and *Lactococcus lactis* [10] are the main bacteria used for the production of this polymer. Bacteria possess the complete pathway for the synthesis of both precursors, whereas yeasts are only naturally capable of synthesizing UDP-GlcNAc. Thus, in addition to *hasA*, HA production in yeast cells requires the expression of the *hasB* gene, which encodes the UDP-glucose 6-dehydrogenase needed for UDP-GlcUA synthesis (Figure 1). To the best of our knowledge, only two yeasts have been engineered for HA production so far: *Kluyveromyces lactis* [11] and *Komagataella phaffii* (previously known as *Pichia pastoris*) [12]. Here, we describe the heterologous production of HA by *Ogataea* (*Hansenula*) *polymorpha*.

In terms of available genetic tools, a set of endogenous promoters was previously described for *O. polymorpha* with different regulatory mechanisms that allow for developing strategies to regulate gene expression [16]. As a methylotrophic yeast, many of these characterized promoters are related to methanol metabolism, controlling the expression of genes encoding enzymes required to metabolize methanol. Additionally, promoters regulating genes related to nitrate metabolism and inducible by this nitrogen source (*YNR1* [17] and *YNI1* [18]) as well as related to temperature increase (*TPS1* and *HSA1* [19]) were described in *O. polymorpha*. Although a set of promoters is available for this non-conventional yeast, the methanol-inducible one is preferred to control heterologous gene expression. Cultivation strategies using these promoters usually are based on two phases, although other approaches based on derepression of these promoters were also reported [20]. The first step is the growth phase, which focuses on biomass production using glucose or glycerol, followed by a methanol induction phase to produce the heterologous protein. Therefore, the utilization of methanol-inducible promoters allows developing strategies that decouple growth and product synthesis, because in the absence of methanol no significant levels of heterologous protein are achieved. Such decoupled strategies are advantageous for synthesis of compounds which metabolic pathways compete with biomass production, such as HA.

In *O. polymorpha*, the promoter which controls the methanol oxidase gene (pMOX, also referred as pAOX, since the methanol oxidase is the primary alcohol oxidase described in this organism [17]) is preferred for heterologous gene expression [21], and its regulatory mechanisms are widely studied [22]. Although it is mainly regulated by methanol and repressed by glucose, it is active under limiting-glucose conditions [23], leading to promoter leakage, which might affect strategies that require gene regulation. Additionally, pMOX is derepressed in the presence of other carbon sources such as glycerol, xylose, ribose, and sorbitol [24]. Other promoters upregulated by methanol and repressed by glucose include pDHAS (dihydroxyacetone synthase) and pFMD (formaldehyde dehydrogenase) [25]. The latter is an alternative to pMOX since it is considered a strong promoter in the presence of methanol. However, a high level of enzyme production (13.5 g/L) has been achieved using glucose as the carbon source [26]. For HA production, the utilization of methanol-inducible promoters is a feasible strategy to avoid competition between its synthesis and biomass production, which is the main limitation for the heterologous production of HA (Figure 1) [27,28]. 

Although methanol-inducible promoters are widely used in heterologous protein expression by *O. polymorpha*, their use may be discouraged due to its leakage under some carbon sources and methanol toxicity and flammability, especially at an industrial scale. Thus, promoters regulated by other inducers or constitutive promoters are available alternatives. The strong constitutive pGAP promoter is commonly used for gene regulation in *O. polymorpha* [29]. Other endogenous constitutive promoters already described for this yeast include pTEF1 [13] and pADH1 [30]. However, this type of promoter does not allow one to tune gene regulation, and the heterologous protein is produced similarly regardless of cultivation conditions. Therefore, an alternative genetic tool must be considered for tunning gene regulation. For example, the utilization of genetic switches such as serine integrases enables the building of genetic circuits through targeted DNA rearrangement. If two recognizing sites (*attB* and *attP*) are inserted flanking the desired DNA sequence, the integrase is capable of identifying these sites and flip the sequence at 180° (see details in [31]). Thus, this strategy works as a genetic tool for gene regulation once any genetic element (promoter, coding sequence, and/or terminator) can be constructed flanked by these *att* sites. The rotation of the desired DNA sequence can occur in a specific condition. Recently, a system using the sites *attB* and *attP* and different serine integrases was applied to build a genetic switch in other eukaryotic cells [32]. 

In this work, the genetic modifications necessary for the heterologous production of HA using *O. polymorpha* as a chassis organism are described (Figure 1). For this, the *hasA* and *hasB* genes were integrated into the *O. polymorpha* genome under different promoters’ regulation. Various combinations were tested to evaluate their influence on HA titers. Two *hasA* genes were used, referred to as *hasAs* (from *Streptococcus zooepidemicus*) and *hasAp* (from *Pasteurella multocida*). The *hasAp* gene was evaluated because, as with the *hasB* (from *Xenopus laevis*) used, we previously demonstrated that the enzymes encoded by these genes were active in *K. lactis*, another non-conventional yeast [11]. The *hasAs* was selected since it is widely used for heterologous production of HA [7,33,34].

Moreover, to better control HA production, we have also employed a genetic switch using a serine integrase to control both *hasAp* and *hasB* gene expression. The integrase-13 (Int13) was selected due to its versatility, successfully applied to design genetic switches in human, bovine, and plant cells [32]. Besides, no point mutations or changes in cell viability caused by the Int13 were detected in cells analyzed in the previous study. Thus, we evaluated a genetic switch using the Int13 as a proof-of-concept in *O. polymorpha* to control the expression of both *hasB* and *hasAp* genes aiming at HA production by this yeast. A capsule-like layer could be seen around cells of the strain containing the genetic switch controlling *hasB* and *hasAp* expression named here as EMB103, using a scanning electron microscopy analysis. In the other strains expressing only *hasB* and *hasA*, the cell surface was similar to the wild-type strain. However, after 48 h of cultivation in an optimized medium, HA was quantified from the culture broth of all three constructed strains tested. This is the first report of an *O. polymorpha* strain developed for HA production to the best of our knowledge.

## 2. Materials and Methods 

### 2.1. Chemicals and Molecular Biology Procedures

Ampicillin, hygromycin, zeocin, and analytical grade HA for quantification standards were purchased from Sigma-Aldrich. T4 DNA Ligase, Taq DNA polymerase, and restriction endonucleases were from Thermo Fisher. The *hasB* (GenBank accession number MH728986), *hasAp* (GenBank accession number MH728990), and *hasAs* (GenBank AF414053.1) genes were each synthesized and delivered in pBSK plasmids by Síntese Biotecnologia, which also supplied DNA oligonucleotides. The genes *hasB* and *hasAp* were selected from a previous study [11]. The nucleotide sequence for all genes is shown in Supplementary Materials, Sequence S1–S3). The pGEM-T Easy vector, used in cloning procedures, was purchased from Promega (Madison, WI, USA).

General molecular biology procedures were conducted according to Sambrook and Russel [35]. All reagents and kits were supplied by Thermo Fisher (Waltham, MA, USA) and used according to their recommendations. The primers used in this study are shown in Table S1. PCR products were purified using the GeneJet PCR Purification Kit from Thermofisher, and fragments treated with restriction enzymes were recovered from agarose gels 0.8% using the GeneJET Gel Extraction Kit. Plasmid cloning was carried out using T4 DNA Ligase after treating inserts and vectors with appropriate restriction endonucleases. Transformation of *Escherichia coli* through heat shock was performed as described previously [36]. Plasmids were extracted using the GeneJet Plasmid Miniprep Kit from Thermofisher.

### 2.2. Construction of Plasmids and Strains

**Figure 2:**
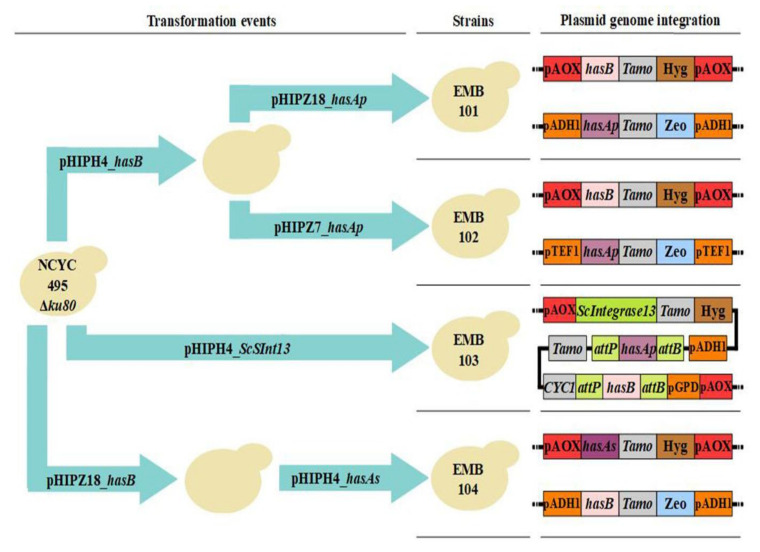
Strains constructed in this study to evaluate HA production in *O. polymorpha*. Light blue arrows represent transformation events with the plasmids specified. Integration of plasmids by single crossover is depicted. Hyg: hygromycin resistance cassette. Zeo: zeocin resistance cassette.

The plasmids and strains used in this study are listed in Tables S2 and S3, respectively. Sequences of constructed plasmids are shown in Supplementary Materials, Sequences S5–S11. The *O. polymorpha* strain NCYC495 *yku80* was used and is referred to as Wild Type (WT). This strain, in which the gene encoding KU80 protein was deleted to diminish recombination by non-homologous end joining (NHEJ), and the plasmids of the pHIP series, used as backbone vectors, were cordially provided by Dr. Ida J van der Klei [37]. The *E. coli* DH10B strain (Thermo Fisher) was used for plasmid cloning and expansion. All plasmids were confirmed by restriction enzyme analysis and PCR. In total, four strains of *O. polymorpha* were constructed from the NCYC495 *yku80* strain. The overall strategy for strain construction and the final strain genotypes are summarized in Figure 2 and described in the Supplementary Material. Stability of construct integration was confirmed thoroughly throughout the study through PCR of colonies resulting from consecutive passaging on selective media plates.

To construct the pHIPH4_*hasB* vector, the *hasB* gene was amplified from pBSK_*hasB* using the primers described in Table S1. Amplification added sites for the restriction enzymes HindIII and SalI to the *hasB* amplicon, which was then ligated into pGEM-T Easy. The resulting pGEM_*hasB* vector and pHIPH4 were treated with HindIII and SalI. The fragment containing *hasB* was purified from an agarose gel for ligation into pHIPH4 between pAOX and the Tamo terminator, resulting in the pHIPH4_*hasB* vector. Similarly, the *hasAp* gene was extracted from the pBSK_*hasAp* vector using HindIII and XhoI, which were also used to remove eGFP-SKL from the pHIPZ18_eGFP-SKL vector. The *hasAp* fragment and the pHIPZ18 backbone were purified from an agarose gel. The former was then ligated between the pADHI promoter and the Tamo terminator of the latter, resulting in the pHIPZ18_*hasAp* vector. The pHIPH4_*hasB* and pHIPZ18_*hasAp* plasmids were inserted into strain NCYC495 *yku80*, resulting in the strain EMB101 (Figure 2).

To construct pHIPZ7_*hasAp*, the pHIPZ18_*hasAp* and pHIPZ7 vectors were treated with HindIII and XhoI and cloned as described above pHIPZ7_*hasAp*, which has the *hasAp* gene under the control of pTEF1 and Tamo terminator. The strain containing the *hasB* gene was transformed with pHIPZ7_*hasAp*, resulting in EMB102 (Figure 2).

The whole pHIPH4_*ScSInt13* plasmid was designed to contain a genetic switch controlling the expression of *hasAp* and *hasB*. The pHIPH4 plasmid was used as a backbone to construct a vector bearing a gene encoding a serine-type phage integrase-13, codon-optimized for *S. cerevisiae*, and the same *hasAp* and *hasB* expression cassettes as in the pHIPZ18_*hasAp* and pHIPH4_*hasB* plasmids. The Integrase13 target sites *attB* and its reverse complement sequence *attP* [38,39] were placed flanking both with genes synthesized in reverse complement orientation [25,26]. The nucleotide sequence for the integrase-13 and the sequences of *attB* and *attP* was based on a previous study [40]. The whole plasmid was synthesized by Epoch Life Science Inc (Missouri City, TX, USA) and confirmed by sequencing and restriction enzyme analysis. It was linearized in the pAOX promoter and transformed into NCYC495 *yku80* to generate EMB103 (Figure 2).

The pHIPZ18_*hasB* plasmid was constructed using HindIII and XbaI to excise *hasB* from pHIPH4_*hasB* and insert it into the pHIPZ18 vector treated with the same enzymes. Fragment and backbone were gel-purified before ligation. The same restriction enzymes were used to generate the pHIPH4_*hasAs* plasmid by cloning *hasAs* from pBSK_*hasAs* into the pHIPH4 backbone. Strain NCYC495 *yku80* was transformed with linearized pHIPZ18_*hasB* and, after confirmation of *hasB* integration in transformants by colony PCR, the pHIPH4_*hasAs* was inserted, resulting in strain EMB104 (Figure 2).

### 2.3. Media and Culture Conditions

*Escherichia coli* cells were cultured in lysogeny broth (LB) at 37 °C and 200 rpm. To select cells harboring plasmids, the medium was supplemented with ampicillin (100 µg/mL).

*O. polymorpha* strains were cultured and maintained in YPD (1% yeast extract, 2% peptone, and 2% glucose) at 37 °C and 200 rpm. Transformant strains were selected and maintained in media supplemented with 100 μg/mL zeocin or 300 μg/mL hygromycin, according to the resistance markers.

### 2.4. Electrocompetent Cell Preparation and Transformation

The *O. polymorpha* electrocompetent cells were prepared as described previously [41], using 175 mM β-mercaptoethanol in a substitution of dithiothreitol TED buffer. Sixty microliters of the freshly prepared electrocompetent cells and 5–10 μg of the plasmid linearized at the *O. polymorpha* promoter sequence with the appropriate restriction enzyme were mixed and transferred into an ice-cold 2-m-gap electroporation cuvette. The pulse was performed using a Bio-Rad Xcell MicroPulser cellular electroporator set to 50 μF, 129 Ω, and 1.5 kV (7.5 kV/cm). The recovered cells were plated onto selective media and incubated at 37 °C.0020

### 2.5. Determination of the Growth Profile

Each constructed strain and the WT were grown overnight in YPD supplemented with 2% glucose and the appropriate antibiotics. On the following day, the cultures were used to inoculate 500 mL baffled shake flasks containing 50 mL of YPD such that initial optical density at 600 nm (OD600) was 0.3. Flasks were then incubated at 37 °C and 200 rpm for biomass production. The cultures were monitored, and samples were taken every two hours during the first 12 h for OD600 measurement using a SpectraMax M2 (Molecular Devices^®^, San Jose, CA, USA). The cultures were started with glucose as the carbon source as a step focused on producing biomass, thus decoupling growth from the heterologous expression of the genes regulated by the pAOX promoter. Consequently, methanol had to be added (1% at 7 and 21 h) to induce the heterologous expression of *has* genes.

At 25 h, cells were transferred to a fresh medium, since glucose is required to synthesize both HA’s precursors (Figure 1). High-density cell cultures were harvested by centrifugation (4000 rpm for 10 min) and transferred to 1-L baffled shake flasks containing 100 mL of medium and kept at 37 °C under agitation (200–250 rpm). The culture ended after 24 h and samples were prepared for Scanning Electron Microscopy (SEM) analysis. All cultures were performed twice.

The maximum growth rate (μMAX) was determined as the slope of the log-transformed linear regression equation. The period of growth used was from 0 h to 6 h, which comprises the exponential growth phase on glucose in the applied conditions.

### 2.6. Statistical Analysis and Data Presentation

**Figure 3:**
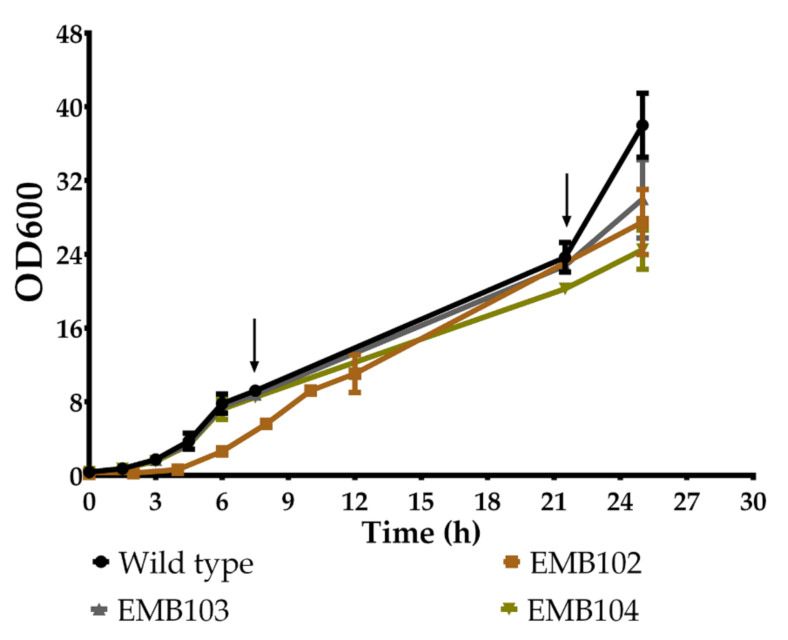
Growth profile of EMB102, EMB103, and EMB104 and *O. polymorpha* NCYC495 *yku80* strain. The experiment was performed in YPD, using shake flasks. The arrows indicate the approximate times of supplementation with methanol to a final concentration of 1%. Means and standard deviations were calculated from two biological replicates.

The parameters µMAX (h^−1^), Final OD, and Doubling time (h), calculated from the growth curves, and HA titer were submitted to analysis of variance (ANOVA) followed by Tukey’s post-test using the Graphpad Prism software (version 6.0). The parameters evaluated follow the criteria to choose the one-way ANOVA test. The growth curve in Figure 3 displays the average values obtained from two biological replicates, with the bars representing the standard deviation (SD). All graphs were prepared using the Graphpad Prism software (version 6.0). All genetic constructions represented in the figures presented in the Supplementary Material were obtained from the software SnapGene^®^ version 5.1.5.

### 2.7. Analysis through Scanning Electron Microscopy (SEM)

Cells from 1 mL aliquots of cultures conducted as described above (Section 2.5) were prepared for SEM as described in [11,42]. Images of the surfaces of the cells were made on a Jeol JSM-7000F field emission scanning electron microscope with the assistance of an Emitech K550 automated sputter coater and an Emitech K850 critical point dryer.

### 2.8. HA Quantification

The protocol applied for HA production and extraction was adapted from a previous study [12]. Cells from each constructed strain and the WT were pre-grown overnight in YPD medium supplemented with appropriate antibiotics, if needed. These cultures were transferred to 1-L baffled shake flasks containing YPD and grown for 24 h aiming a high-cell density culture. Additionally, methanol was added to 1% at 7 and 21 h for induction of *has* genes, as described in Section 2.5. After the 24 h, cells were harvested and washed using a saline solution (9 g/L of NaCl), then transferred to new 1-L baffled shake flasks containing an optimized medium for HA production [43]: 4% glucose, 0.75% yeast extract, 1% peptone, 0.25% K_2_HPO_4_, 0.05% MgSO_4_, 0.5% NaCl, 0.04% glutamine, 0.06% glutamic acid, and 0.02% oxalic acid. Glucose was added to 2% during the cultivation to avoid glucose-starvation as indicated by the OD_600_ measured. The cultivation ended after 48 h and cultures were prepared for HA quantification.

For HA extraction, 100 mL samples from each culture were purified as described by [12]. Briefly, an equal volume of 0.1% (w/v) SDS solution was added, then the samples were kept under orbital agitation for 10 min, so that the HA capsule was separated from the cells after being centrifuged for 5 min at 4500× *g*. 1.5 volumes of ethanol were added to the supernatant, which was incubated at 4 °C overnight, for at least 10 h. Then, samples were centrifuged at 4500× *g* and 4 °C for 30 min. The pellet was washed with 25 mL of a 75% ethanol and 25% 0.15 M NaCl solution, then centrifuged once more. The supernatant was discarded, and the pellet was incubated at room temperature and resuspended in MilliQ water after the ethanol evaporated. The WT strain culture was used as a negative control. The quantifications were performed in three technical replicates from each biological replicate.

The carbazole method was used to quantify HA as previously described [44]. Firstly, 200 μL of water (blank), HA standard and purified samples were added to ice-cold glass tubes with 500 μL of a 25 mM solution of sodium tetraborate in sulfuric acid. After homogenizing, the tubes were incubated at 100 °C under agitation for 10 min and kept at room temperature for cooling. Twenty μL of a 0.125% carbazole solution on methanol was added to each tube. After a second incubation (100 °C for 15 min under agitation), the samples were cooled to room temperature prior reading. The 200 μL volume of each tube was applied to a 96-well microplate, which was read at 530 nm by the Biotek EON microplate reader. The samples, as well as the points of the standard curve, were analyzed in technical triplicate (Supplementary Materials, Figures S16 and S17). The ten-point standard curve used comprised the concentrations of 10 μg/mL, 20 μg/mL, 50 μg/mL, 100 μg/mL, 200 μg/mL, 300 μg/mL, 400 μg/mL, 500 μg/mL, 600 μg/mL, and 700 μg/mL.

## 3. Results and Discussion

### 3.1. Strain Construction

Table 1 lists the strains developed in this study, including the promoters utilized for each gene as well as the origin of the heterologous genes. Integration of synthetic constructs into their genomes and their genetic stability were assessed through consecutive cultivation in plates containing antibiotics, followed by PCR. Figures of the 1% agarose gels are shown in Supplementary Material (Figures S1–S5).

The presence of *hasB* in strain EMB101 was confirmed by colony PCR. However, even though EMB101 was capable of growing in the presence of the pHIPZ18 plasmid selection marker, zeocin, the presence of *hasAp* was not detected by colony PCR after the third passage in the selective medium (Supplementary Materials, Figure S2). For this reason, EMB101 was not used in further experiments. Assessment of *hasAp* instability when integrated into the *ADH1* locus has not been investigated further. Both *hasA* and *hasB* could be verified in strains EMB102, EMB103, and EMB104 (Supplementary Materials, Figures S3–S5). For the strain EMB103, in which Int13 was used as a genetic switch to control the expression of *hasA* and *hasB*, PCR analysis confirmed the rotation of both genes (Supplementary Materials, Figure S4). Thus, these strains were used in subsequent experiments.

### 3.2. Growth Profile of Recombinant Strains

Decoupling cell growth and HA production is crucial to its synthesis. Thus, the cultivation was divided into two steps: biomass and enzymes production, and HA synthesis. For the first step, the constructed strains EMB102, EMB103, and EMB104 as well the WT strain were grown on shake flasks containing YPD aiming at achieving high-cell density cultures (Figure 3). Since in the presence of glucose the pAOX promoter is repressed [23], no HA synthesis is expected in this step. At the end of the exponential phase estimated by the OD600 measured (7 h), methanol was added to the cultures to induce the expression of *has* genes required for HA production. Methanol was also added at 21 h of cultivation to ensure high levels of enzymes for HA production (Figure 3). Therefore, this first step of cultivation comprised biomass production and accumulation of the enzymes required for HA synthesis.

While the strains had similar specific growth rate during the exponential phase, they reached different final ODs (Table 2). Nevertheless, the highest mean of final OD, from the WT strain, was statistically different only for the EMB104 strain (*p* < 0.05). This negative impact on cell growth for the engineered strains could be related to (i) a high-level of heterologous protein production after induction using methanol or (ii) production of HA, which impacts cell growth. Indeed, the competition between HA production and cell growth was previously described [6,28].

For the *K. lactis* HA-producing yeast, HA production had a negative impact on final cell density [11]. This might be linked to the fact that the HA precursor UDP-GlcNAc is a cell wall component for yeasts, and thus, HA synthesis competes with biomass formation. As methanol is necessary for the expression of the HA synthesis enzymes on the constructed strains, the competition between biomass and HA production only becomes noticeable after the addition of methanol.

After the first phase of growth for biomass generation and induction of *has* genes (Figure 3), the entire culture was transferred to fresh YPD medium, aiming at HA production. In this case, the production was assessed by SEM.

### 3.3. HA Production Assessed by Scanning Electron Microscopy Analysis

Given that HA is produced and secreted by the cells, a capsular structure can be visualized on their surface. This method was reported previously to confirm the production of HA by *L. lactis* [45] and by the yeast *K. lactis* [11]. The formation of a capsule-like layer was also observed in HA-producing strains of *C. glutamicum* [46], although a phase-contrast microscopy analysis was applied instead of SEM. In this case, the capsule-like layer affected nutrient uptake and cell metabolism, which impaired HA production [46].

**Figure 4:**
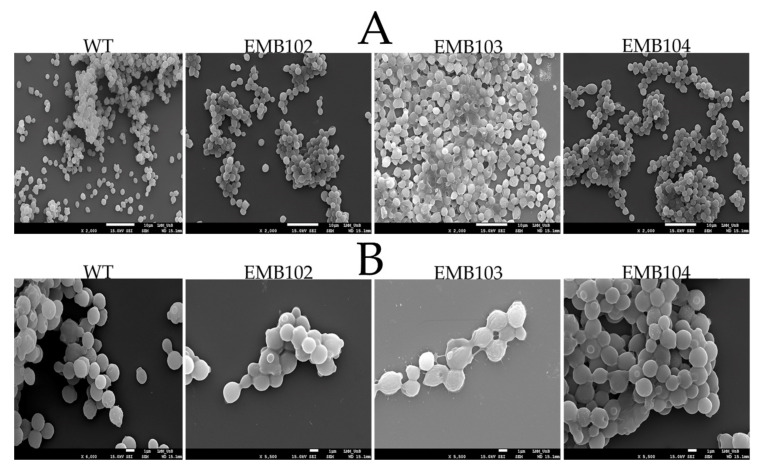
Scanning Electron Microscopy of different *O. polymorpha* strains. The names above the image correspond to the respective strain. Images were acquired at two magnifications: (**A**) 2000× and (**B**) 5500×.

**Figure 5:**
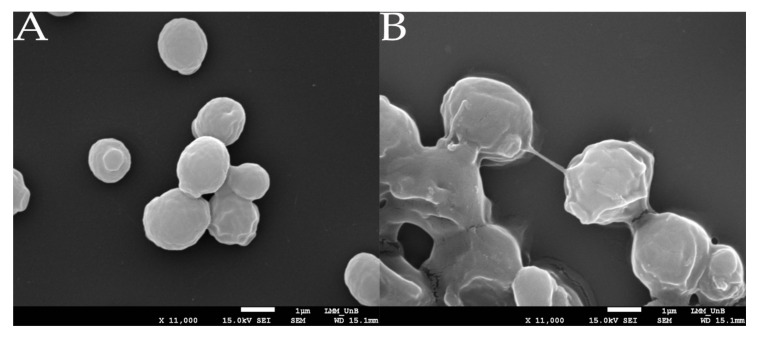
Scanning Electron Microscopy of Wild Type (WT) (**A**) and EMB103 (**B**) in the magnitude of 11,000×.

As seen on SEM images from 24 h cultures, WT cells are clearly discrete, with separate cell walls and moderate aggregation. Cells from the EMB102 and EMB104 strains showed no significant superficial differences compared to the WT strain, although a discrete capsule-like structure was observed (Figure 4B). Strain EMB103 is clearly distinct from the others. Firstly, discrete cell walls between cells are not distinguishable at the same magnification as in the other strains (Figure 4B). Likewise, seemingly intensified aggregation is observed, and free individual cells are less common. Lastly, EMB103 cells seem to be covered in a substance that glues them together and generates an irregular cell surface indicating the production of HA (Figure 5).

The EMB103 strain was constructed containing a genetic switch for the expression of *hasAp* and *hasB* which allows expression of both genes after the induction of a gene encoding a serine-like integrase-13. Thus, the formation of a capsule-like layer by the EMB103 cells indicates the genetic switch constructed for *O. polymorpha* works properly. Recently, the utilization of integrases to design genetic circuits for gene regulation is emerging as a powerful synthetic biology tool [31]. Serine integrases are also used as a genetic tool to mediate in vivo site-specific recombination between an exogenous DNA fragment, such as plasmids or gene expression cassettes, into a target genome [47]. This has become a versatile system since its application was demonstrated in prokaryotes [48] and higher eukaryotes such as human, bovine, and plant cells [32]. For yeast cells, several integrases showed activity when expressed by *S. cerevisiae*, with the highest activity reached by the ϕBT1 integrase [49]. Additionally, the authors verified no site damage by integrase-mediated recombination, which is important to reduce point mutations during the site-specific recombination events. In another study using baker’s yeast, the PhiC31 integrase was used to successfully recycle the selective markers HIS5 and LEU2 [50]. However, it was verified that the system was efficient only when a low-copy vector bearing the integrase-encoding gene was used, probably due to the toxicity of PhiC31 integrase. The Int13 utilized in this study showed no point mutation or impact on cell viability when tested in different eukaryotic cells [23], representing a reliable genetic tool for yeast cells. Nevertheless, there is no report of a genetic switch using serine integrases for gene regulation in non-conventional yeasts. Here, as a proof of concept, the system was applied to construct an *O. polymorpha* strain that produces HA and showed to be the best genetic approach for constructing HA producing *O. polymorpha* strains.

### 3.4. HA Quantification

Since a discrete difference between the cell surface of EMB102 and EMB104 compared to the WT was observed, it was hypothesized that HA production could be limited by growth conditions as well as the HA purification protocol applied. Therefore, an adaptation from the previous protocol applied for *K. phaffi* was performed [12], which is an optimized medium for HA. By this approach, it was possible to quantify the HA in the supernatant from all constructed strains (Table 2). After 48 h of culture, HA titers were assessed through the carbazole method, which was able to accurately quantify the production of HA (Table 2 and Supplementary Materials, Figure S17).

The highest titer of HA (197.76 μg/mL) was achieved by strain EMB103, although its result is not statistically different from that of the EMB102 strain (151.20 μg/mL). The EMB104 strain achieved the lowest HA concentration in the study (123.20 μg/mL). Furthermore, both HAS encoding genes (*hasAp* and *hasAs*) seem to work properly in *O. polymorpha*, indicating that this yeast is a suitable host platform for the application of different strategies for HA production.

Apparently, HA production in *O. polymorpha* in the conditions applied is limited leading to a low synthesis by all strains. Nevertheless, these results demonstrate the potential of *O. polymorpha* as a platform host for HA production, although the process requires optimization regarding cell cultivation. For instance, for the *K.phaffi* HA-producing strain, cultivation was performed in fermenters using a fed-batch mode with controlled dissolved oxygen levels and only 200 mg/L of HA was detected after 48 h of culture when both *hasA* and *hasB* were expressed [12]. Therefore, the conditions used here to produce, and isolate HA are sub-optimal. Furthermore, the purification of HA was conducted solely on the supernatant of cultures. Yet, it has been determined that the HAS enzyme from *P. multocida* serogroup A:1, differently from other HAS, is actually cytosolic and does not possess an HA exporting function [51]. Indeed, there is published evidence of up to 80% of heterologously produced HA inside of algae host cells [52]. While it is established that HA is secreted to the extracellular medium simultaneously with its synthesis by streptococcal HAS enzymes, strains EMB 102 and EMB 103, harboring the *hasAp* gene, might still produce intracellular HA. This evidence suggests that lysates of cultured cells might also be sources for HA quantification and their use in quantification in addition to the supernatant should be considered, depending on the experimental setup and enzymes used.

Additionally, the balance between the two precursors plays a key role in HA production [53,54]. Intracellular concentrations of precursors present a challenge for HA production, and equimolar concentration of UDP-GlcNAc and UDP-GlcUA is required for efficient synthesis of HA. In this regard, the availability of UDP-GlcNAc presents a hurdle, for it is diverted to cell wall formation in *O. polymorpha* [55]. Lastly, the Pentose Phosphate Pathway (PPP) deviates Glucose-6-phosphate which is required for HA precursors biosynthesis. Downregulation of the *zwf* gene, which encodes for the first enzyme of PPP, is a well-described strategy for improving HA production [8,9,56]. The *O. polymorpha* NCYC495 strain, when grown at temperatures above 30 °C, showed an increased flux towards PPP higher than the other thermotolerant yeast tested, *Kluyveromyces marxianus* [57]. It is worth mentioning that PPP and methanol assimilation are intrinsically related in *Ogataea* sp. [58] as well as in other methylotrophic yeasts [59]. Xylulose-5-phosphate, an intermediate from PPP, is required for methanol assimilation, specifically for formaldehyde fixation by the enzyme dihydroxyacetone synthase (Figure 1). In fact, in *O. parapolymorpha* (previously known as *Hansenula polymorpha* DL-1), genes encoding enzymes from the non-oxidative phase of PPP are upregulated in the presence of methanol [58]. Therefore, PPP flux is an important bottleneck to be considered for the heterologous production of HA by *O. polymorpha*. Strategies aiming at funneling the metabolic framework of *O. polymorpha* are required to ensure a proper flux through HA precursors and its efficient synthesis.

Nevertheless, the production of up to 197.76 µg/mL of HA by *O. polymorpha* has been reported, demonstrating the potential of this non-conventional yeast for production of HA, although several aspects of the process require further optimization, as discussed above.

## 4. Conclusions

*O. polymorpha* was engineered for HA production. Both *hasB* (*X. laevis*) and two different *hasA* genes, from *P. multocida* (*hasAp*) and *S. zooepidemicus* (*hasAs*), were successfully integrated into the *O. polymorpha* genome under the control of different promoters. Additionally, a genetic switch using a serine-type integrase-13 to control the expression of both *hasAp* and *hasB* was demonstrated. Both the qualitative and quantitative methods were able to show HA synthesis by the yeast. The capsule-like structure observed in EMB103 cells by SEM analysis indicates HA production by this strain. Indeed, the EMB103 was able to produce 197.76 µg/mL, which is considered a low HA titer. Additionally, it was demonstrated that both HAS encoding genes from *P. multocida* and *S. zooepidemicus* can be utilized in *O. polymorpha*, which enables the design of different strategies for HA production in this industrial yeast. These results demonstrate not only the potential of *O. polymorpha* as a host platform for HA production but also of integrases for gene regulation in this yeast.

## Figures and Tables

**Table 1:** Summary of all EMB strains constructed in this study.

EMB Strains	Promoter Driving *hasA*	Source of *hasA*	Promoter Driving *hasB*	Source of *hasB*
**EMB102**	pTEF1	*P. multocida*	pAOX	*Xenopus laevis*
**EMB103**	pADH1	*P. multocida*	pGPD (*S. cerevisiae*)	*X. laevis*
**EMB104**	pAOX	*S. zooepidemicus*	pADH1	*X. laevis*

**Table 2:** The final OD_600_ and maximum specific growth rates (μMAX) from wild type and EMB strains as well as the HA titer obtained by EMB strains when cultivated in shake flasks.

Strains	μMAX (h^−1^) *	Final OD *	Doubling Time (h) *	Hyaluronic Acid (HA) Titer (μg/mL) **	Final OD **
**WT**	0.50 ± 0.02 ^a^	38.00 ± 3.46 ^a^	1.35 ± 0.06 ^a^	NA	21.00 ± 1.41 ^a^
**EMB102**	0.45 ± 0.04 ^a^	27.50 ± 3.54 ^a,b^	1.55 ± 0.15 ^a^	151.20 ± 13.04 ^a,b^	27.00 ± 0.00 ^a^
**EMB103**	0.52 ± 0.01 ^a^	30.00 ± 4.24 ^a,b^	1.34 ± 0.01 ^a^	197.76 ± 5.66 ^b^	27.50 ± 0.71 ^a^
**EMB104**	0.49 ± 0.02 ^a^	24.50 ± 2.12 ^b^	1.41 ± 0.07 ^a^	123.20 ± 26.56 ^a^	21.50 ± 3.54 ^a^

NA: Not applied. Equal letters indicate no statistical difference between the strains (*p* < 0.05). * Parameters were calculated based on the data obtained by the growth curves presented in Figure 3 and described in Material and Methods (Section 2.5). ** Values were obtained after 48 h of cultivation in medium optimized for HA production [12,43].

## Data Availability

The data presented in this study are available in the text and Supplementary Materials.

## Supplementary Materials

The following are available online at https://www.mdpi.com/2076-2607/9/2/312/s1. Table S1: List of primers used in this work; Table S2: List of plasmids used in this work; Table S3: List of strains used in this work; Sequences S1–S4: Gene sequences; Sequences S5–S11: Sequences of constructed vectors; Figures S1–S5: Confirmation of constructed strains; Figures S6–S15: Plasmid maps; Figure S16: Carbazole standard curve; Figure S17: Carbazole assay plate picture.
