# Supplementary material for: Evaluation of Ogataea (Hansenula) polymorpha for Hyaluronic Acid Production
Source: Microorganisms. 2021 Feb 3;9(2):312. doi: 10.3390/microorganisms9020312 (PMC7913781; doi:10.3390/microorganisms9020312)
Supplement: Supplementary file 1 [file microorganisms-09-00312-s001.pdf]

Supplementary Material

Evaluation of *Ogataea* (*Hansenula*) *polymorpha* for hyaluronic acid production

João Heitor Colombelli Manfrão-Netto[1], Enzo Bento Queiroz[1], Kelly Assis Rodrigues[1], Cintia M. Coelho[2], Hugo Costa Paes[3], Elíbio Leopoldo Rech Filho[4], Nádia Skorupa Parachin*[1,5]

[1] Grupo Engenharia de Biocatalisadores, Instituto de Ciências Biológicas, Universidade de Brasília, Brasília-DF, Brazil

[2] Department of Genetics and Morphology, Institute of Biological Science, University of Brasília, Brasília, 70910900, DF, Brazil

[3] Clinical Medicine Division, University of Brasília Medical School, University of Brasília, Brasília, Federal District 70910-900, Brazil

[4] Brazilian Agriculture Research Corporation – Embrapa – Genetic Resources and Biotechnology – CENARGEN, Brasília 70770917 DF, Brazil.

[5] Present address: Ginkgo Bioworks 27th Drydock Ave. 8th floor, Boston, MA, 02210.

*Correspondence: nadiasp@unb.br; nadiasp@gmail.com

Summary of Supporting information

Table S1. List of primers used in this work --- 3

Table S2. List of plasmids used in this work --- 4

Table S3. List of strains used in this work -- 5

Sequence S1. The nucleotide sequence of *hasB* gene from *Xenopus laevis* --- 6

Sequence S2. The nucleotide sequence of *hasAp* from *Pasteurella multocida* -------------------------------------- 6

Sequence S3. The nucleotide sequence of *hasAs* from *Streptococcus zooepidemicus* ------------------------------ 7

Sequence S4. The nucleotide sequence of Int13 encoding gene, codon-optimized for *S. cerevisiae*----------- 8

Sequence S5. The nucleotide sequence of the pGEM_hasB plasmid --- 9

Sequence S6. The nucleotide sequence of the pHIPZ7_hasAp plasmid --- 11

Sequence S7. The nucleotide sequence of the pHIPZ18_hasAp plasmid --------------------------------------- 15

Sequence S8. The nucleotide sequence of the pHIPZ18_hasB plasmid --- 19

Sequence S9. The nucleotide sequence of the pHIPH4_hasB plasmid -- 22

Sequence S10. The nucleotide sequence of the pHIPH4_hasAs plasmid --- 25

Sequence S11. The nucleotide sequence of the pHIPH4_ScSInt13 plasmid -------------------------------------- 29

Figure S1. Confirmation of gene integration on the EMB101 strain -- 35

Figure S2. Confirmation of gene stability on the EMB101 strain --- 36

Figure S3. Confirmation of gene integration on the EMB102 strain -- 37

Figure S4. Scheme representing the genetic switch constructed --- 38

Figure S5. Confirmation of gene integration on the EMB104 strain -- 39

Figure S6. Map of the synthetic plasmid pBSK_*hasB* -- 40

Figure S7. Map of the synthetic plasmid pBSK_*hasAp* -- 40

Figure S8. Map of the synthetic plasmid pBSK_*hasAs* -- - 41

Figure S9. Map of the pGEM_*hasB* plasmid -- 41

Figure S10. Map of the pHIPZ7_*hasAp* plasmid -- 42

Figure S11. Map of the pHIPZ18_*hasAp* plasmid -- 42

Figure S12. Map of the pHIPZ18_*hasB* plasmid --- 43

Figure S13. Map of the pHIPH4_*hasB* plasmid --- 43

Figure S14. Map of the pHIPH4_*hasAs* plasmid --- 44

Figure S15. Map of the pHIPH4_*ScSInt13* plasmid -- 44

Figure S16. Standard curve obtained for the Carbazole method --- 45

Figure S17. Carbazole assay plate picture --- 45

Table S1. List of primers used in this work

Primer name	Sequence to amplify	Primer orientation	Restriction enzyme 5′	[2]Primer sequence 5′→3′
attB_hasB_F	*attL* (*hasB*)	Forward	NA	CCAGTTCCCTGAAATTATTCCCCT
attP_hasB_R	*attL* (*hasB*)	Reverse	NA	CAGCTTCTTGAATTGCACCATCAAT
attB_hasAp_F	*attL* (*hasAp*)	Forward	NA	CTCTTCTTAGGCATCCTTCTATC
attP_hasAp_R	*attL* (*hasAp*)	Reverse	NA	GCAGCACTAGCTTGGAAACC
hasB_F	*hasB*	Forward	HindIII	AAA**AAGCTT**AATGTTTCAGATCAAGAAGATTTGTTGTA
hasB_R	*hasB*	Reverse	SalI	AAA**GTCGAC**TTACACACGTTGCTTC
hasAp_F	*hasAp*	Forward	HindIII	AAGCTT**AAGCTT**ATGAATACCTTATCTC
hasAp_R	*hasAp*	Reverse	XhoI	AAAGTCGACGCCTTTACAATGTGATTGA
AOX-Integration_F[1]	*AOX* promoter	Forward	NA	CAGTTTTTGCCCTACTTGATC
AOX-Integration_R[1]	*AMO*[3] terminator	Reverse	NA	GTAGGAAGGCTGGATGTC

NA: Not applied

[1]These primers were designed to confirm the insert integration when the plasmid pHIPH4 is used for cloning.

[2]In bold, the recognizing sequence for the respective enzyme.

[3] *AMO*: Amino oxidase

Table S2. List of plasmids used in this work

Name	Description	Reference
pBSK_*hasB*	Cloning vector carrying the synthetic *hasB* gene encoding the UDP-glucose dehydrogenase from *Xenopus laevis*	This work
pBSK_*hasAp*	Cloning vector carrying the synthetic *hasAp* gene encoding the hyaluronic acid synthase from *Pasteurella multocida*	This work
pBSK_*hasAs*	Cloning vector carrying the synthetic *hasAs* gene encoding the hyaluronic acid synthase from *Streptococcus zooepidemicus*	This work
pGEM-T Easy	Commercial plasmid used in the cloning steps	Promega
pGEM_*hasB*	pGEM-T Easy plasmid carrying the *hasB* gene	This work
pHIPH4	*O. polymorpha* integrative plasmid containing the native promoter pAOX and the terminator Tamo	[1]
pHIPZ7	*O. polymorpha* integrative plasmid containing the native promoter pTEF1 and the terminator Tamo	[1]
pHIPZ18_eGFP_SKL	*O. polymorpha* integrative plasmid containing the native promoter pAHD1 and the terminator Tamo and carrying the gene encoding eGFP	[1]
pHIPH4_*hasB*	pHIPH4-derived plasmid carrying the *hasB* gene under control of the pAOX promoter	This work
pHIPH4_*hasAs*	pHIPH4-derived plasmid carrying the *hasAs* gene under control of the pAOX promoter	This work
pHIPH4_*ScSInt13*	Synthetic plasmid containing a genetic switch controlling the expression of *hasAp* and *hasB* and carrying a gene of a serine-type phage integrase-13, codon-optimized for *S. cerevisiae*	This work
pHIPZ7_*hasAp*	pHIPZ7-derived plasmid carrying the *hasAp* gene under control of the pTEF1 promoter	This work
pHIPZ18_*hasAp*	pHIPZ18-derived plasmid carrying the *hasAp* gene under control of the pADH1 promoter	This work
pHIPZ18_*hasB*	pHIPZ18-derived plasmid carrying the *hasB* gene under control of the pADH1 promoter	This work

Table S3. List of strains used in this work

Name	Description	Reference
E. coli DH10B	Bacteria strain for plasmid cloning	Invitrogen
O. polymorpha NCYC 495 *yku80*	Methylotrophic yeast with a NHEJ-deficient phenotype; referred to as Wild Type (WT) in this work	[1]
EMB100.1	WT strain transformed with the linearized pHIPH4_*hasB* plasmid. The *hasB* gene was integrated into the genome under control of the pAOX promoter. This strain was used as an intermediate for the construction of the EMB101 and EMB102 strains.	This work
EMB101	EMB100.1 transformed with the linearized pHIPZ18_*hasAp* plasmid. The *hasAp* gene was integrated into the genome under control of the pADH1 promoter. The *hasB* gene is regulated by the pAOX promoter.	This work
EMB102	EMB100.1 transformed with the linearized pHIPZ7_*hasAp* plasmid. The *hasAp* gene was integrated into the genome under control of the pTEF1 promoter. The *hasB* gene is regulated by the pAOX promoter.	This work
EMB103	WT strain transformed with the linearized pHIPH4_ScsInt13 containing a genetic switch controlling the expression of *hasAp* and *hasB* and carrying a gene for a serine-type phage integrase-13, codon-optimized for *S. cerevisiae* and under control of the pAOX promoter. The genes *hasB* and *hasAp* are regulated by the pGDP promoter from *S. cerevisiae* and the pADH1 promoter from *O. polymorpha*, respectively.	This work
EMB100.2	WT strain transformed with the linearized pHIPZ18_*hasB* plasmid. The *hasB* gene was integrated into the genome under control of the pAHD1 promoter. This strain was used as an intermediate for the construction of EMB104.	This work
EMB104	EMB100.2 transformed with the linearized pHIPH4_*hasAs* plasmid. The *hasAs* gene was integrated into the genome under control of the pAOX promoter. The *hasB* gene is regulated by the pADH1 promoter.	This work

Sequence S1. Nucleotide sequence of the *hasB* gene from *Xenopus laevis* (Genbank ID: MH728986). The start and stop codons of the ORF are shown in bold.

ATGTTTCAGATCAAGAAGATTTGTTGTATTGGTGCCGGTTACGTCGGTGGTCCAACCTGT
TCTGTCATTGCACAGATGTGCCCTGACATTAAGGTCACTGTTGTGGATGTGAACCAAGC
CAGGATCAATGCTTGGAATAGTGACACTTTGCCTATCTACGAACCAGGTTTGAAGGAAG
TCGTAGAGTCATGCAGGGGAAAGAATTTGTTCTACTCAACTGACATTGATGGTGCAATT
CAAGAAGCTGATTTGGTGTTCATCTCAGTCAACACTCCAACAAAAACTTACGGTATGGG
TAAGGGAAGGGCAGCCGACTTGAAATACATTGAGGCTTGCGCTAGAAGAATAGTACAG
AATAGTAACGGATACAAGATTGTTACAGAGAAATCTACTGTGCCAGTTAGAGCTGCTG
AATCAATAAGACGTATCTTCGATGCAAATACTAAACCAGATTTGAACTTGCAGGTATTG
AGTAACCCAGAGTTTTTGGCAGAGGGTACAGCCATTAAGGATTTGAAGAACCCTGATA
GAGTTTTGATAGGTGGTGACGAAACCCCTGAAGGTCAGAAAGCTGTTAGAGCTTTGTGT
GACGTATACGAACACTGGGTACCATCTGAGAAAATCATAACCACAAACACCTGGTCTT
CTGAGTTGAGTAAGTTAGCAGCCAACGCATTCTTAGCTCAAAGAATTTCTTCAATCAAC
TCAATTAGTGCCTTATGTGAAGCCACAGGAGCTGACGTTGAAGAGGTTGCCAGAGCTAT
TGGTATGGATCAAAGAATTGGTAACAAGTTTTTGAAGGCTTCAGTGGGATTTGGAGGTT
CATGTTTTCAGAAGGACGTTTTGAACTTGGTTTACTTGTGTGAGGTGTTAAACTTGCACG
AAGTGGCCAAGTACTGGCAACAAGTGATTGATATGAATGATTATCAAAGGAGACGTTT
TACAACTAGGATAATCGATTGTTTGTTTAACACCGTGACCGATAAGAAAATCGCATTGT
TAGGTTTCGCTTTCAAGAAGGATACAGGTGATACTAGAGAGAGTAGTTCAATCTATATC
TCTAAGTATTTGATGGATGAAGGTGCTAAGTTACATATCTACGATCCAAAGGTCCCACG
TGAGCAGATCATCACTGACTTGAGTCAACCTGGTGTTGCAGCTGACGACAGGGTTTCTC
AATTGGTCCACATAAGTACAGATTTGTACGAAGCCTGTGAGAATGCACACGCTATGGTC
ATTTGTACTGAATGGGATATGTTCAAGGAATTAGATTTCAATAGAATCCATAGGATGAT
GTTAAAGCCTGCTTTCATATTCGATGGTAGACGTGTTTTAGATGAATTGCATGGAGAATT
GCAAAACATTGGATTTCAGGTGGAAACCATCGGAAAGAAGGTAGCTTCAAAAAGAAT
ACCATTCACTCCAACTGCTGATATCCCTAAGTTCGGTTTACAGGACTTGCCACACAAGA
AGCAACGTGTG**TAA**

Sequence S2. Nucleotide sequence of the *hasAp* gene from *Pasteurella multocida* (Genbank ID: MH728990). The start and stop codons of the ORF are shown in bold.

ATGAATACCTTATCTCAAGCCATCAAGGCATACAATTCAAATGACTATCAATTGGCTTT
GAAATTGTTCGAAAAGTCAGCAGAAATCTACGGACGTAAGATAGTAGAGTTTCAGATT
ACTAAGTGCAAGGAGAAATTGTCTGCCCATCCAAGTGTCAATTCAGCTCATCCTTCAGT
CAACTCAGCACATTTGTCAGTAAACAAAGAGGAAAAGGTTAATGTGTGCGATTCTCCAT
TGGATATTGCTACCCAATTGTTGTTGTCAAATGTCAAGAAATTAGTGTTATCTGACAGTG
AGAAGAACACATTGAAGAATAAGTGGAAGTTGTTAACTGAAAAGAAAAGTGAGAATG
CTGAAGTTCGTGCTGTAGCATTGGTACCAAAGGATTTCCCAAAGGATTTGGTGTTGGCA
CCATTACCTGACCATGTGAATGACTTTACTTGGTACAAGAAGAGAAAGAAACGTTTAG
GTATCAAACCAGAACATCAACACGTGGGTTTGTCAATAATTGTCACCACTTTCAATCGT
CCTGCAATCTTAAGTATAACTTTGGCATGCTTAGTTAATCAAAAGACTCACTATCCATTC
GAGGTGATTGTCACAGATGATGGATCACAAGAAGATTTGTCTCCAATCATAAGACAAT
ATGAAAACAAATTGGATATCCGTTATGTCAGACAAAAGGACAATGGTTTCCAAGCTAG
TGCTGCTAGGAATATGGGTTTGAGATTAGCAAAGTATGATTTCATTGGTTTGTTGGATTG

CGATATGGCACCTAACCCATTATGGGTGCATTCATATGTCGCTGAATTGTTAGAAGATG
ATGATTTGACAATCATTGGACCAAGAAAGTACATTGATACACAACATATCGACCCAAA
GGACTTCTTAAACAATGCATCTTTGTTGGAATCATTGCCAGAAGTTAAGACCAATAACT
CAGTGGCCGCAAAAGGTGAAGGTACCGTTTCATTGGATTGGAGGTTGGAGCAATTCGA
AAAGACTGAAAACTTAAGATTGTCAGACTCTCCTTTTAGATTCTTCGCAGCTGGTAATGT
TGCTTTCGCCAAGAAGTGGTTGAACAAATCTGGATTCTTTGATGAAGAGTTCAACCATT
GGGGTGGTGAAGATGTTGAGTTTGGATATAGATTGTTTAGGTATGGTTCATTCTTCAAGA
CTATTGACGGTATCATGGCCTACCATCAAGAGCCACCTGGTAAGGAAAACGAAACAGA
TAGGGAAGCTGGAAAGAACATCACATTGGATATTATGAGGGAGAAGGTACCATATATT
TACAGGAAGTTGTTGCCTATCGAAGATTCACACATCAATAGAGTCCCTTTGGTTTCTATC
TATATCCCAGCTTACAACTGTGCCAATTATATTCAACGTTGTGTTGATTCTGCCTTGAAC
CAGACAGTTGTAGATTTGGAAGTCTGTATTTGCAATGATGGTTCTACAGATAATACTTTG
GAAGTTATCAACAAGTTGTACGGTAACAATCCAAGAGTCAGAATCATGAGTAAACCAA
ATGGTGGTATTGCTAGTGCTTCTAATGCAGCAGTGAGTTTTGCCAAAGGATATTACATA
GGTCAATTAGATTCAGATGACTATTTGGAGCCAGATGCCGTAGAGTTATGTTTGAAAGA
GTTCTTGAAAGACAAAACTTTGGCTTGTGTATATACAACAAACAGAAATGTCAATCCTG
ATGGTTCTTTGATAGCAAATGGTTACAACTGGCCAGAGTTTAGTAGGGAGAAGTTGACT
ACTGCAATGATTGCTCATCACTTCCGTATGTTCACTATCAGGGCATGGCATTTGACCGAT
GGTTTTAATGAGAAGATTGAGAATGCTGTGGACTACGATATGTTCTTGAAGTTGAGTGA
AGTTGGTAAGTTCAAGCACTTAAACAAAATCTGCTATAACAGGGTATTGCATGGTGATA
ATACAAGTATTAAGAAGTTGGGTATCCAAAAGAAGAACCATTTCGTGGTCGTCAACCA
GAGTTTGAACAGGCAAGGAATCACTTACTACAATTACGACGAGTTCGATGACTTAGAT
GAGTCTAGGAAATACATCTTTAACAAAACAGCTGAGTACCAGGAAGAAATTGACATCT
TAAAGGACATTAAGATCATACAAAACAAGGACGCTAAAATAGCAGTATCTATCTTCTA
CCCAAATACTTTGAATGGTTTGGTCAAGAAATTGAATAACATCATCGAGTACAACAAG
AACATATTCGTTATTGTCTTGCATGTGGACAAGAACCATTTGACCCCAGATATCAAGAA
AGAGATATTGGCTTTCTACCACAAGCATCAAGTGAATATTTTGTTGAATAACGATATCT
CATACTACACATCAAACCGTTTAATCAAGACCGAGGCACATTTATCAAACATTAATAA
GTTGTCACAGTTGAACTTGAATTGTGAATATATCATATTCGACAATCATGACTCTTTGTT
CGTGAAGAATGATTCTTATGCCTATATGAAGAAGTACGATGTTGGTATGAATTTCTCAG
CCTTAACTCATGATTGGATTGAAAAGATTAACGCACATCCACCATTCAAGAAGTTGATT
AAGACATACTTTAACGATAATGACTTGAAATCTATGAACGTTAAAGGAGCTAGTCAAG
GAATGTTTATGACATATGCATTGGCTCACGAATTGTTGACTATTATCAAAGAGGTTATCA
CTTCTTGCCAATCTATCGATTCTGTACCAGAATACAACACTGAGGACATATGGTTTCAAT
TTGCATTGTTGATCTTGGAAAAGAAAACTGGTCATGTCTTTAACAAGACAAGTACCTTG
ACATACATGCCTTGGGAGAGGAAGTTGCAATGGACCAATGAACAAATTGAATCAGCTA
AACGTGGAGAAAACATTCCAGTGAACAAGTTCATAATCAATTCAATCACATTG**TAA**

Sequence S3. Nucleotide sequence of the *hasAs* gene from *Streptococcus zooepidemicus* (Genbank ID: AF414053.1). The start and stop codons of the ORF are shown in bold.

ATGAGAACTTTGAAAAATTTAATTACAGTTGTTGCATTCTCAATTTTCTGGGTTTTATTA
ATTTATGTTAATGTTTATTTGTTCGGTGCTAAAGGTTCATTGTCAATTTATGGTTTCTTATT
AATTGCATATTTGTTAGTTAAAATGTCTTTGTCATTCTTCTATAAACCATTCAAAGGTAG
AGCAGGTCAATATAAAGTTGCTGCAATTATTCCATCATATAATGAAGATGCTGAATCTT

TATTGGAAACTTTGAAATCAGTTCAACAACAAACATATCCATTGGCAGAAATTTATGTT
GTTGATGATGGTTCAGCCGATGAAACAGGTATTAAAAGAATTGAAGATTATGTTAGAG
ATACTGGTGATTTATCATCTAATGTTATTGTTCATCGTAGCGAAAAAAATCAAGGTAAA
AGACATGCACAAGCATGGGCTTTCGAAAGAAGCGATGCAGATGTTTTCTTGACAGTTGA
TTCAGATACATATATTTATCCAGATGCTTTGGAAGAATTATTGAAAACTTTCAATGATCC
TACAGTTTTCGCAGCTACAGGTCATTTGAATGTTAGAAATAGACAAACTAATTTGTTAA
CAAGATTGACTGATATTAGATATGATAATGCATTCGGTGTTGAAAGAGCAGCACAATCA
GTTACTGGTAATATTTTAGTTTGTTCTGGTCCTTTGTCAGTTTATAGAAGAGAAGTTGTTG
TTCCTAATATTGATAGATATATTAATCAAACTTTCTTAGGTATTCCAGTTTCAATTGGTGA
TGATAGATGTTTAACTAATTATGCTACTGATTTGGGTAAAACAGTTTATCAATCTACAGC
TAAATGTATTACTGATGTTCCAGATAAAATGTCTACATATTTGAAACAACAAAATAGAT
GGAATAAATCATTCTTCAGAGAATCTATTATTTCAGTTAAAAAAATTATGAATAATCCA
TTCGTTGCTTTGTGGACTATTTTGGAAGTTTCTATGTTCATGATGTTAGTTTATTCAGTTGT
TGATTTCTTCGTTGATAATGTTAGAGAGTTCGATTGGTTAAGAGTTTTGGCATTCTTGGTT
ATTATTTTCATTGTTGCTTTGTGTAGAAATATTCATTATATGTTGAAACATCCTTTGTCTTT
CTTGTTATCTCCTTTCTATGGTGTTTTACATTTATTCGTTTTGCAACCATTGAAATTGTATT
CATTGTTCACTATTAGAAATGCTGATTGGGGTACTAGAAAAAAATTGTTG**TAA**

Sequence S4. Nucleotide sequence of the gene of serine integrase-13 (Int13), codon-optimized for *S. cerevisiae*. The start and stop codons of the ORF are shown in bold.

ATGGCGGTTGGGATTTACATCAGAGTCTCAACCCAAGAGCAGGCGAGTGAAGGGCACA
GTATTGAAAGCCAAAAAAAGAAACTGGCGTCTTATTGTGAAATCCAAGGATGGGATGA
CTACAGGTTCTACATCGAAGAGGGCATATCCGGGAAAAACACAAATAGACCGAAGCTT
AAGCTATTAATGGAACATATCGAAAAGGGAAAAATTAACATTTTATTGGTCTACAGGCT
GGATAGGTTGACTAGGTCTGTGATCGATTTACATAAGCTATTAAACTTTTTACAGGAAC
ATGGGTGCGCGTTTAAATCTGCTACAGAAACTTACGACACAACTACTGCAAACGGAAG
GATGAGTATGGGTATAGTGAGTCTTCTAGCCCAATGGGAGACAGAAAATATGAGTGAG
CGTATTAAACTAAACCTGGAACACAAAGTCTTGGTTGAGGGGGAAAGAGTAGGGGCGA
TTCCCTATGGATTCGACTTGTCAGATGATGAAAAGCTTGTGAAGAATGAAAAGTCTGCA
ATTTTATTGGACATGGTCGAAAGGGTGGAGAACGGCTGGTCCGTCAATAGGATCGTCAA
CTATCTTAATTTAACTAACAATGATCGTAACTGGTCACCTAATGGGGTGCTACGTTTGTT
AAGGAACCCTGCACTATATGGCGCTACAAGGTGGAATGATAAAATCGCAGAGAACAC
ACACGAGGGTATAATTAGCAAGGAACGTTTCAACCGTCTGCAGCAAATACTTGCAGAC
CGTAGCATCCATCACAGACGTGATGTGAAAGGAACATACATATTTCAAGGAGTTTTGA
GATGTCCGGTTTGTGATCAGACGCTGTCCGTTAATAGGTTTATTAAGAAGCGTAAGGAT
GGAACAGAGTACTGTGGTGTCCTTTATAGGTGTCAGCCATGTATTAAGCAAAACAAGTA
CAATTTAGCTATCGGCGAAGCTAGGTTCCTGAAGGCCCTTAACGAGTACATGTCTACGG
TGGAATTCCAGACAGTTGAAGACGAGGTGATACCCAAGAAAAGTGAGAGAGAAATGT
TGGAATCTCAGCTGCAACAGATCGCAAGAAAGAGGGAGAAATACCAAAAGGCATGGG
CGAGCGATTTAATGTCCGATGATGAATTTGAGAAACTTATGGTCGAGACCCGTGAAACT
TATGACGAATGCAAGCAAAAACTGGAGAGTTGCGAGGACCCTATTAAGATCGACGAG
ACATATTTGAAGGAAATAGTTTACATGTTTCATCAAACATTCAATGATTTAGAGTCCGA
GAAGCAAAAGGAGTTTATATCAAAATTTATAAGGACTATCCGTTACACCGTCAAAGAG
CAGCAACCTATCAGACCTGATAAGTCTAAGACAGGTAAGGGTAAACAGAAAGTGATA
ATTACGGAAGTGGAGTTTTACCAG**TAA**

Sequence S5. The nucleotide sequence of the pGEM_*hasB* plasmid. The sequence of the *hasB* gene is shown in yellow.

TATAGTGAGTCGTATTACAATTCACTGGCCGTCGTTTTACAACGTCGTGACTGGGAAAA
CCCTGGCGTTACCCAACTTAATCGCCTTGCAGCACATCCCCCTTTCGCCAGCTGGCGTA
ATAGCGAAGAGGCCCGCACCGATCGCCCTTCCCAACAGTTGCGCAGCCTGAATGGCGA
ATGGACGCGCCCTGTAGCGGCGCATTAAGCGCGGCGGGTGTGGTGGTTACGCGCAGCG
TGACCGCTACACTTGCCAGCGCCCTAGCGCCCGCTCCTTTCGCTTTCTTCCCTTCCTTTCT
CGCCACGTTCGCCGGCTTTCCCCGTCAAGCTCTAAATCGGGGGCTCCCTTTAGGGTTCCG
ATTTAGTGCTTTACGGCACCTCGACCCCAAAAAACTTGATTAGGGTGATGGTTCACGTA
GTGGGCCATCGCCCTGATAGACGGTTTTTCGCCCTTTGACGTTGGAGTCCACGTTCTTTA
ATAGTGGACTCTTGTTCCAAACTGGAACAACACTCAACCCTATCTCGGTCTATTCTTTTG
ATTTATAAGGGATTTTGCCGATTTCGGCCTATTGGTTAAAAAATGAGCTGATTTAACAA
AAATTTAACGCGAATTTTAACAAAATATTAACGCTTACAATTTCCTGATGCGGTATTTTC
TCCTTACGCATCTGTGCGGTATTTCACACCGCATCAGGTGGCACTTTTCGGGGAAATGTG
CGCGGAACCCCTATTTGTTTATTTTTCTAAATACATTCAAATATGTATCCGCTCATGAGA
CAATAACCCTGATAAATGCTTCAATAATATTGAAAAAGGAAGAGTATGAGTATTCAAC
ATTTCCGTGTCGCCCTTATTCCCTTTTTTGCGGCATTTTGCCTTCCTGTTTTTGCTCACCCA
GAAACGCTGGTGAAAGTAAAAGATGCTGAAGATCAGTTGGGTGCACGAGTGGGTTACA
TCGAACTGGATCTCAACAGCGGTAAGATCCTTGAGAGTTTTCGCCCCGAAGAACGTTTT
CCAATGATGAGCACTTTTAAAGTTCTGCTATGTGGCGCGGTATTATCCCGTATTGACGCC
GGGCAAGAGCAACTCGGTCGCCGCATACACTATTCTCAGAATGACTTGGTTGAGTACTC
ACCAGTCACAGAAAAGCATCTTACGGATGGCATGACAGTAAGAGAATTATGCAGTGCT
GCCATAACCATGAGTGATAACACTGCGGCCAACTTACTTCTGACAACGATCGGAGGAC
CGAAGGAGCTAACCGCTTTTTTGCACAACATGGGGGATCATGTAACTCGCCTTGATCGT
TGGGAACCGGAGCTGAATGAAGCCATACCAAACGACGAGCGTGACACCACGATGCCT
GTAGCAATGGCAACAACGTTGCGCAAACTATTAACTGGCGAACTACTTACTCTAGCTTC
CCGGCAACAATTAATAGACTGGATGGAGGCGGATAAAGTTGCAGGACCACTTCTGCGC
TCGGCCCTTCCGGCTGGCTGGTTTATTGCTGATAAATCTGGAGCCGGTGAGCGTGGGTCT
CGCGGTATCATTGCAGCACTGGGGCCAGATGGTAAGCCCTCCCGTATCGTAGTTATCTA
CACGACGGGGAGTCAGGCAACTATGGATGAACGAAATAGACAGATCGCTGAGATAGG
TGCCTCACTGATTAAGCATTGGTAACTGTCAGACCAAGTTTACTCATATATACTTTAGAT
TGATTTAAAACTTCATTTTTAATTTAAAAGGATCTAGGTGAAGATCCTTTTTGATAATCT
CATGACCAAAATCCCTTAACGTGAGTTTTCGTTCCACTGAGCGTCAGACCCCGTAGAAA
AGATCAAAGGATCTTCTTGAGATCCTTTTTTTCTGCGCGTAATCTGCTGCTTGCAAACAA
AAAAACCACCGCTACCAGCGGTGGTTTGTTTGCCGGATCAAGAGCTACCAACTCTTTTT
CCGAAGGTAACTGGCTTCAGCAGAGCGCAGATACCAAATACTGTTCTTCTAGTGTAGCC
GTAGTTAGGCCACCACTTCAAGAACTCTGTAGCACCGCCTACATACCTCGCTCTGCTAA

TCCTGTTACCAGTGGCTGCTGCCAGTGGCGATAAGTCGTGTCTTACCGGGTTGGACTCA
AGACGATAGTTACCGGATAAGGCGCAGCGGTCGGGCTGAACGGGGGGTTCGTGCACAC
AGCCCAGCTTGGAGCGAACGACCTACACCGAACTGAGATACCTACAGCGTGAGCTATG
AGAAAGCGCCACGCTTCCCGAAGGGAGAAAGGCGGACAGGTATCCGGTAAGCGGCAG
GGTCGGAACAGGAGAGCGCACGAGGGAGCTTCCAGGGGGAAACGCCTGGTATCTTTAT
AGTCCTGTCGGGTTTCGCCACCTCTGACTTGAGCGTCGATTTTTGTGATGCTCGTCAGGG
GGGCGGAGCCTATGGAAAAACGCCAGCAACGCGGCCTTTTTACGGTTCCTGGCCTTTTG
CTGGCCTTTTGCTCACATGTTCTTTCCTGCGTTATCCCCTGATTCTGTGGATAACCGTATT
ACCGCCTTTGAGTGAGCTGATACCGCTCGCCGCAGCCGAACGACCGAGCGCAGCGAGT
CAGTGAGCGAGGAAGCGGAAGAGCGCCCAATACGCAAACCGCCTCTCCCCGCGCGTTG
GCCGATTCATTAATGCAGCTGGCACGACAGGTTTCCCGACTGGAAAGCGGGCAGTGAG
CGCAACGCAATTAATGTGAGTTAGCTCACTCATTAGGCACCCCAGGCTTTACACTTTAT
GCTTCCGGCTCGTATGTTGTGTGGAATTGTGAGCGGATAACAATTTCACACAGGAAACA
GCTATGACCATGATTACGCCAAGCTATTTAGGTGACACTATAGAATACTCAAGCTATGC
ATCCAACGCGTTGGGAGCTCTCCCATATGGTCGACCTGCAGGCGGCCGCGAATTCACTA
GTGATTAAAAAGCTTAATGTTTCAGATCAAGAAGATTTGTTGTATTGGTGCCGGTTACGT
CGGTGGTCCAACCTGTTCTGTCATTGCACAGATGTGCCCTGACATTAAGGTCACTGTTGT
GGATGTGAACCAAGCCAGGATCAATGCTTGGAATAGTGACACTTTGCCTATCTACGAAC
CAGGTTTGAAGGAAGTCGTAGAGTCATGCAGGGGAAAGAATTTGTTCTACTCAACTGA
CATTGATGGTGCAATTCAAGAAGCTGATTTGGTGTTCATCTCAGTCAACACTCCAACAA
AAACTTACGGTATGGGTAAGGGAAGGGCAGCCGACTTGAAATACATTGAGGCTTGCGC
TAGAAGAATAGTACAGAATAGTAACGGATACAAGATTGTTACAGAGAAATCTACTGTG
CCAGTTAGAGCTGCTGAATCAATAAGACGTATCTTCGATGCAAATACTAAACCAGATTT
GAACTTGCAGGTATTGAGTAACCCAGAGTTTTTGGCAGAGGGTACAGCCATTAAGGATT
TGAAGAACCCTGATAGAGTTTTGATAGGTGGTGACGAAACCCCTGAAGGTCAGAAAGC
TGTTAGAGCTTTGTGTGACGTATACGAACACTGGGTACCATCTGAGAAAATCATAACCA
CAAACACCTGGTCTTCTGAGTTGAGTAAGTTAGCAGCCAACGCATTCTTAGCTCAAAGA
ATTTCTTCAATCAACTCAATTAGTGCCTTATGTGAAGCCACAGGAGCTGACGTTGAAGA
GGTTGCCAGAGCTATTGGTATGGATCAAAGAATTGGTAACAAGTTTTTGAAGGCTTCAG
TGGGATTTGGAGGTTCATGTTTTCAGAAGGACGTTTTGAACTTGGTTTACTTGTGTGAGG
TGTTAAACTTGCACGAAGTGGCCAAGTACTGGCAACAAGTGATTGATATGAATGATTAT
CAAAGGAGACGTTTTACAACTAGGATAATCGATTGTTTGTTTAACACCGTGACCGATAA
GAAAATCGCATTGTTAGGTTTCGCTTTCAAGAAGGATACAGGTGATACTAGAGAGAGT
AGTTCAATCTATATCTCTAAGTATTTGATGGATGAAGGTGCTAAGTTACATATCTACGAT
CCAAAGGTCCCACGTGAGCAGATCATCACTGACTTGAGTCAACCTGGTGTTGCAGCTGA
CGACAGGGTTTCTCAATTGGTCCACATAAGTACAGATTTGTACGAAGCCTGTGAGAATG
CACACGCTATGGTCATTTGTACTGAATGGGATATGTTCAAGGAATTAGATTTCAATAGA

ATCCATAGGATGATGTTAAAGCCTGCTTTCATATTCGATGGTAGACGTGTTTTAGATGAA
TTGCATGGAGAATTGCAAAACATTGGATTTCAGGTGGAAACCATCGGAAAGAAGGTAG
CTTCAAAAAGAATACCATTCACTCCAACTGCTGATATCCCTAAGTTCGGTTTACAGGAC
TTGCCACACAAGAAGCAACGTGTGTAAGTCGACTTTAATCGAATTCCCGCGGCCGCCAT
GGCGGCCGGGAGCATGCGACGTCGGGCCCAATTCGCCC

Sequence S6. The nucleotide sequence of the pHIPZ7_*hasAp* plasmid. The sequence of the *hasAp* gene is shown in green.

AGGGGATATCCTCGAGACTTGCCTTTGAAGGCTCTTGTTGCGGTAAATAAGTATATAGG
ACACGACAATCTAGTAATCTCCACTATTGACGAGCTCGTCGAACTGCGAAAATAGGTTT
TCCATCTGGTCTGTAGGCATCAGCCCGGCGTCATCCTCCTGCGCAGGAGCAGCGGGCTC
AGGGCCGGCCTGGGCGGGCTGATCCAGAAAGTCGAGGTTCAGATCCCCCACACACCAT
AGCTTCAAAATGTTTCTACTCCTTTTTTACTCTTCCAGATTTTCTCGGACTCCGCGCATCG
CCGTACCACTTCAAAACACCCAAGCACAGCATACTAAATTTCCCTCTTTCTTCCTCTAG
GGTGTCGTTAATTACCCGTACTAAAGGTTTGGAAAAGAAAAAAGAGACCGCCTCGTTTC
TTTTTCTTCGTCGAAAAAGGCAATAAAAATTTTTATCACGTTTCTTTTTCTTGAAATTTTTT
TTTTTAGTTTTTTTCTCTTTCAGTGACCTCCATTGATATTTAAGTTAATAAACGGTCTTCAA
TTTCTCAAGTTTCAGTTTCATTTTTCTTGTTCTATTACAACTTTTTTTACTTCTTGTTCATTA
GAAAGAAAGCATAGCAATCTAATCTAAGGGGCGGTGTTGACAATTAATCATCGGCATA
GTATATCGGCATAGTATAATACGACAAGGTGAGGAACTAAACCATGGCCAAGTTGACC
AGTGCCGTTCCGGTGCTCACCGCGCGCGACGTCGCCGGAGCGGTCGAGTTCTGGACCG
ACCGGCTCGGGTTCTCCCGGGACTTCGTGGAGGACGACTTCGCCGGTGTGGTCCGGGAC
GACGTGACCCTGTTCATCAGCGCGGTCCAGGACCAGGTGGTGCCGGACAACACCCTGG
CCTGGGTGTGGGTGCGCGGCCTGGACGAGCTGTACGCCGAGTGGTCGGAGGTCGTGTCC
ACGAACTTCCGGGACGCCTCCGGGCCGGCCATGACCGAGATCGGCGAGCAGCCGTGGG
GGCGGGAGTTCGCCCTGCGCGACCCGGCCGGCAACTGCGTGCACTTCGTGGCCGAGGA
GCAGGACTGACACGTCCGACGGCGGCCCACGGGTCCCAGGCCTCGGAGATCCGTCCCC
CTTTTCCTTTGTCGATATCATGTAATTAGTTATGTCACGCTTACATTCACGCCCTCCCCCC
ACATCCGCTCTAACCGAAAAGGAAGGAGTTAGACAACCTGAAGTCTAGGTCCCTATTT
ATTTTTTTATAGTTATGTTAGTATTAAGAACGTTATTTATATTTCAAATTTTTCTTTTTTTTC
TGTACAGACGCGGTACCCAGCTTTTGTTCCCTTTAGTGAGGGTTAATTCCGAGCTTGGCG
TAATCATGGTCATAGCTGTTTCCTGTGTGAAATTGTTATCCGCTCACAATTCCACACAAC
ATACGAGCCGGAAGCATAAAGTGTAAAGCCTGGGGTGCCTAATGAGTGAGCTAACTCA
CATTAATTGCGTTGCGCTCACTGCCCGCTTTCCAGTCGGGAAACCTGTCGTGCCAGCTGC
ATTAATGAATCGGCCAACGCGCGGGGAGAGGCGGTTTGCGTATTGGGCGCTCTTCCGCT
TCCTCGCTCACTGACTCGCTGCGCTCGGTCGTTCGGCTGCGGCGAGCGGTATCAGCTCA
CTCAAAGGCGGTAATACGGTTATCCACAGAATCAGGGGATAACGCAGGAAAGAACAT

GTGAGCAAAAGGCCAGCAAAAGGCCAGGAACCGTAAAAAGGCCGCGTTGCTGGCGTT
TTTCCATAGGCTCCGCCCCCCTGACGAGCATCACAAAAATCGACGCTCAAGTCAGAGG
TGGCGAAACCCGACAGGACTATAAAGATACCAGGCGTTTCCCCCTGGAAGCTCCCTCG
TGCGCTCTCCTGTTCCGACCCTGCCGCTTACCGGATACCTGTCCGCCTTTCTCCCTTCGGG
AAGCGTGGCGCTTTCTCATAGCTCACGCTGTAGGTATCTCAGTTCGGTGTAGGTCGTTCG
CTCCAAGCTGGGCTGTGTGCACGAACCCCCCGTTCAGCCCGACCGCTGCGCCTTATCCG
GTAACTATCGTCTTGAGTCCAACCCGGTAAGACACGACTTATCGCCACTGGCAGCAGCC
ACTGGTAACAGGATTAGCAGAGCGAGGTATGTAGGCGGTGCTACAGAGTTCTTGAAGT
GGTGGCCTAACTACGGCTACACTAGAAGGACAGTATTTGGTATCTGCGCTCTGCTGAAG
CCAGTTACCTTCGGAAAAAGAGTTGGTAGCTCTTGATCCGGCAAACAAACCACCGCTG
GTAGCGGTGGTTTTTTTGTTTGCAAGCAGCAGATTACGCGCAGAAAAAAAGGATCTCAA
GAAGATCCTTTGATCTTTTCTACGGGGTCTGACGCTCAGTGGAACGAAAACTCACGTTA
AGGGATTTTGGTCATGAGATTATCAAAAAGGATCTTCACCTAGATCCTTTTAAATTAAA
AATGAAGTTTTAAATCAATCTAAAGTATATATGAGTAAACTTGGTCTGACAGTTACCAA
TGCTTAATCAGTGAGGCACCTATCTCAGCGATCTGTCTATTTCGTTCATCCATAGTTGCC
TGACTCCCCGTCGTGTAGATAACTACGATACGGGAGGGCTTACCATCTGGCCCCAGTGC
TGCAATGATACCGCGAGACCCACGCTCACCGGCTCCAGATTTATCAGCAATAAACCAG
CCAGCCGGAAGGGCCGAGCGCAGAAGTGGTCCTGCAACTTTATCCGCCTCCATCCAGT
CTATTAATTGTTGCCGGGAAGCTAGAGTAAGTAGTTCGCCAGTTAATAGTTTGCGCAAC
GTTGTTGCCATTGCTACAGGCATCGTGGTGTCACGCTCGTCGTTTGGTATGGCTTCATTC
AGCTCCGGTTCCCAACGATCAAGGCGAGTTACATGATCCCCCATGTTGTGCAAAAAAG
CGGTTAGCTCCTTCGGTCCTCCGATCGTTGTCAGAAGTAAGTTGGCCGCAGTGTTATCAC
TCATGGTTATGGCAGCACTGCATAATTCTCTTACTGTCATGCCATCCGTAAGATGCTTTT
CTGTGACTGGTGAGTACTCAACCAAGTCATTCTGAGAATAGTGTATGCGGCGACCGAGT
TGCTCTTGCCCGGCGTCAATACGGGATAATACCGCGCCACATAGCAGAACTTTAAAAGT
GCTCATCATTGGAAAACGTTCTTCGGGGCGAAAACTCTCAAGGATCTTACCGCTGTTGA
GATCCAGTTCGATGTAACCCACTCGTGCACCCAACTGATCTTCAGCATCTTTTACTTTCA
CCAGCGTTTCTGGGTGAGCAAAAACAGGAAGGCAAAATGCCGCAAAAAAGGGAATAA
GGGCGACACGGAAATGTTGAATACTCATACTCTTCCTTTTTCAATATTATTGAAGCATTT
ATCAGGGTTATTGTCTCATGAGCGGATACATATTTGAATGTATTTAGAAAAATAAACAA
ATAGGGGTTCCGCGCACATTTCCCCGAAAAGTGCCACCTGGGAAATTGTAAACGTTAAT
ATTTTGTTAAAATTCGCGTTAAATTTTTGTTAAATCAGCTCATTTTTTAACCAATAGGCCG
AAATCGGCAAAATCCCTTATAAATCAAAAGAATAGACCGAGATAGGGTTGAGTGTTGT
TCCAGTTTGGAACAAGAGTCCACTATTAAAGAACGTGGACTCCAACGTCAAAGGGCGA
AAAACCGTCTATCAGGGCGATGGCCCACTACGTGAACCATCACCCTAATCAAGTTTTTT
GGGGTCGAGGTGCCGTAAAGCACTAAATCGGAACCCTAAAGGGAGCCCCCGATTTAGA
GCTTGACGGGGAAAGCCGGCGAACGTGGCGAGAAAGGAAGGGAAGAAAGCGAAAGG

AGCGGGCGCTAGGGCGCTGGCAAGTGTAGCGGTCACGCTGCGCGTAACCACCACACCC
GCCGCGCTTAATGCGCCGCTACAGGGCGCGTCGCGCCATTCGCCATTCAGGCTGCGCAA
CTGTTGGGAAGGGCGATCGGTGCGGGCCTCTTCGCTATTACGCCAGCTGGCGAAAGGG
GGATGTGCTGCAAGGCGATTAAGTTGGGTAACGCCAGGGTTTTCCCAGTCACGACGTTG
TAAAACGACGGCCAGTGAATTGTAATACGACTCACTATAGGGCGAATTGGAGCTCCAC
CGCGGTGGCGGCCGCTCTAGCATGGAACCAAGACCCATGACGTTGTTTCTTGATGATCT
CTTTTCGTTTCTGCTTTAATGTTTTTATTTGCTGATCGATTGCCTCTACATCGTTTTTCTGC
ACATCGCTTGAATCGCAGTCGTTATTTGTTTCCACTTTTTCTTTCGGGTTTGACAACCTCC
TCGATTCGTTGACAGTATCAAACACATCGGTAAAAGAACTGCTACGTGAAGTCTTGTCA
GGCTCAGGTATCATTTCCTCTGACTTTTCTTCCTCCTTTTGGCTTTCATGAGAGAACTGCT
TCTCGGAATCACACACACTATCTAGTGTTCGGTCTGCATCTTCTGGCGTCGAATGCTCGG
GCTCAAAGCATAGAATGTCCAAGTTTGAGGGCTCTGTTTGGAACTGCTCCAATTCTTTGC
TCGTTGGTCTTTGGAATAAATTGTTGTTGAGTAGTTTTGATCGAACTGGAGAGTCCCTAT
AATTGCGATTTGTATGTGCTGCAAGAATATGTGTAGAAGCAGTATTATGGATACTTCTCA
AAGAGCTACTTCTCACCGATACCTCGTCCTTGAACGAGACCGTTTTTTTACCCAATTGCT
CCTCGTCCACTAGTTTGTTTGGTTTCATGATGGACTTCAGCATTGAGCTATGTAGATCAC
CTTCTGGGCCTCCTGGGTTGTCTGGCTCAACTTGCTCTTTGTCATCATGTACAAAGGATA
ATGGGATGGTGGTGACTTTTGGAAACTGTACAATGGGGTGAGTATTGAGTTGTAATGTC
CCCCAGACATTCAGAAGGTCAGGCTTGGGAGCAGGCTTTTTACTCAGCCTCAAACTACC
GTTTCTCCGCCTTCGTCTTCTTGACACAGTTGATACGCTGTTGGAAATGGAATGGTCGTC
AGTCTCGTTTAGCCCTAAAGACACCGTTGCCATAATTAGGGATAAATCTATTTAGGCTC
CTTATTTTTCACTCGGCGCGAAAAAAAATGGGCACACGGCGATCGACGAGAGGTCGAT
CGTCTCTACGTTCCAGCTACATGGCAGTTCTAAGACGGGAAGTAAGATGACACTAGTAG
ATGTTTTGCAAATTAGGATCACAGGCCTGCTCCAGAAAACTTTTTATCCTTCAGCGGAA
GGTCTGTCCAGGGGCACAAATCTCAAACAGAGCAAAGGCACCCTTACCCTCTTATCTCC
TTGATGAATTTTTTTTTTCTTTTAAATTCTTTTTTAGAAGGCCGGGTAACAACTTTAAGAA
CCTGTTTCAGCTGTCCCCAGGAAGCTCCATTGGCAGTTCCAGTACGTTGTAGATGTGTAT
AGTGTGCTGAAAAGCGTTGTCCCTGGTTTTTTCAAGCAAAATCTTCGTCTCGGAGCTGGA
TAGCCCACCAAGGTATTGTTCCTGTGCGTAATTTTTGGCACGCAGACGACTCGAATAAG
TTTGGCAATAAAAAAATTTTTTTCACTATATAAAGAGGAGACATTCCCACATGAGATTT
TTTCTGATCTTTTAATTAGTACATTCGTAGGATCCAAGCTTATGAATACCTTATCTCAAG
CCATCAAGGCATACAATTCAAATGACTATCAATTGGCTTTGAAATTGTTCGAAAAGTCA
GCAGAAATCTACGGACGTAAGATAGTAGAGTTTCAGATTACTAAGTGCAAGGAGAAAT
TGTCTGCCCATCCAAGTGTCAATTCAGCTCATCCTTCAGTCAACTCAGCACATTTGTCAG
TAAACAAAGAGGAAAAGGTTAATGTGTGCGATTCTCCATTGGATATTGCTACCCAATTG
TTGTTGTCAAATGTCAAGAAATTAGTGTTATCTGACAGTGAGAAGAACACATTGAAGAA
TAAGTGGAAGTTGTTAACTGAAAAGAAAAGTGAGAATGCTGAAGTTCGTGCTGTAGCA

TTGGTACCAAAGGATTTCCCAAAGGATTTGGTGTTGGCACCATTACCTGACCATGTGAA
TGACTTTACTTGGTACAAGAAGAGAAAGAAACGTTTAGGTATCAAACCAGAACATCAA
CACGTGGGTTTGTCAATAATTGTCACCACTTTCAATCGTCCTGCAATCTTAAGTATAACT
TTGGCATGCTTAGTTAATCAAAAGACTCACTATCCATTCGAGGTGATTGTCACAGATGA
TGGATCACAAGAAGATTTGTCTCCAATCATAAGACAATATGAAAACAAATTGGATATC
CGTTATGTCAGACAAAAGGACAATGGTTTCCAAGCTAGTGCTGCTAGGAATATGGGTTT
GAGATTAGCAAAGTATGATTTCATTGGTTTGTTGGATTGCGATATGGCACCTAACCCATT
ATGGGTGCATTCATATGTCGCTGAATTGTTAGAAGATGATGATTTGACAATCATTGGAC
CAAGAAAGTACATTGATACACAACATATCGACCCAAAGGACTTCTTAAACAATGCATC
TTTGTTGGAATCATTGCCAGAAGTTAAGACCAATAACTCAGTGGCCGCAAAAGGTGAA
GGTACCGTTTCATTGGATTGGAGGTTGGAGCAATTCGAAAAGACTGAAAACTTAAGATT
GTCAGACTCTCCTTTTAGATTCTTCGCAGCTGGTAATGTTGCTTTCGCCAAGAAGTGGTT
GAACAAATCTGGATTCTTTGATGAAGAGTTCAACCATTGGGGTGGTGAAGATGTTGAGT
TTGGATATAGATTGTTTAGGTATGGTTCATTCTTCAAGACTATTGACGGTATCATGGCCT
ACCATCAAGAGCCACCTGGTAAGGAAAACGAAACAGATAGGGAAGCTGGAAAGAAC
ATCACATTGGATATTATGAGGGAGAAGGTACCATATATTTACAGGAAGTTGTTGCCTAT
CGAAGATTCACACATCAATAGAGTCCCTTTGGTTTCTATCTATATCCCAGCTTACAACTG
TGCCAATTATATTCAACGTTGTGTTGATTCTGCCTTGAACCAGACAGTTGTAGATTTGGA
AGTCTGTATTTGCAATGATGGTTCTACAGATAATACTTTGGAAGTTATCAACAAGTTGTA
CGGTAACAATCCAAGAGTCAGAATCATGAGTAAACCAAATGGTGGTATTGCTAGTGCT
TCTAATGCAGCAGTGAGTTTTGCCAAAGGATATTACATAGGTCAATTAGATTCAGATGA
CTATTTGGAGCCAGATGCCGTAGAGTTATGTTTGAAAGAGTTCTTGAAAGACAAAACTT
TGGCTTGTGTATATACAACAAACAGAAATGTCAATCCTGATGGTTCTTTGATAGCAAAT
GGTTACAACTGGCCAGAGTTTAGTAGGGAGAAGTTGACTACTGCAATGATTGCTCATCA
CTTCCGTATGTTCACTATCAGGGCATGGCATTTGACCGATGGTTTTAATGAGAAGATTGA
GAATGCTGTGGACTACGATATGTTCTTGAAGTTGAGTGAAGTTGGTAAGTTCAAGCACT
TAAACAAAATCTGCTATAACAGGGTATTGCATGGTGATAATACAAGTATTAAGAAGTTG
GGTATCCAAAAGAAGAACCATTTCGTGGTCGTCAACCAGAGTTTGAACAGGCAAGGAA
TCACTTACTACAATTACGACGAGTTCGATGACTTAGATGAGTCTAGGAAATACATCTTT
AACAAAACAGCTGAGTACCAGGAAGAAATTGACATCTTAAAGGACATTAAGATCATAC
AAAACAAGGACGCTAAAATAGCAGTATCTATCTTCTACCCAAATACTTTGAATGGTTTG
GTCAAGAAATTGAATAACATCATCGAGTACAACAAGAACATATTCGTTATTGTCTTGCA
TGTGGACAAGAACCATTTGACCCCAGATATCAAGAAAGAGATATTGGCTTTCTACCAC
AAGCATCAAGTGAATATTTTGTTGAATAACGATATCTCATACTACACATCAAACCGTTT
AATCAAGACCGAGGCACATTTATCAAACATTAATAAGTTGTCACAGTTGAACTTGAATT
GTGAATATATCATATTCGACAATCATGACTCTTTGTTCGTGAAGAATGATTCTTATGCCT
ATATGAAGAAGTACGATGTTGGTATGAATTTCTCAGCCTTAACTCATGATTGGATTGAA

AAGATTAACGCACATCCACCATTCAAGAAGTTGATTAAGACATACTTTAACGATAATG
ACTTGAAATCTATGAACGTTAAAGGAGCTAGTCAAGGAATGTTTATGACATATGCATTG
GCTCACGAATTGTTGACTATTATCAAAGAGGTTATCACTTCTTGCCAATCTATCGATTCT
GTACCAGAATACAACACTGAGGACATATGGTTTCAATTTGCATTGTTGATCTTGGAAAA
GAAAACTGGTCATGTCTTTAACAAGACAAGTACCTTGACATACATGCCTTGGGAGAGG
AAGTTGCAATGGACCAATGAACAAATTGAATCAGCTAAACGTGGAGAAAACATTCCAG
TGAACAAGTTCATAATCAATTCAATCACATTGTAAAGGCCTAA

Sequence S7. The nucleotide sequence of the pHIPZ18_*hasAp* plasmid. The sequence of the *hasAp* gene is shown in green.

TCGAGACTTGCCTTTGAAGGCTCTTGTTGCGGTAAATAAGTATATAGGACACGACAATC
TAGTAATCTCCACTATTGACGAGCTCGTCGAACTGCGAAAATAGGTTTTCCATCTGGTCT
GTAGGCATCAGCCCGGCGTCATCCTCCTGCGCAGGAGCAGCGGGCTCAGGGCCGGCCT
GGGCGGGCTGATCCAGAAAGTCGAGGTTCAGATCCCCCACACACCATAGCTTCAAAAT
GTTTCTACTCCTTTTTTACTCTTCCAGATTTTCTCGGACTCCGCGCATCGCCGTACCACTT
CAAAACACCCAAGCACAGCATACTAAATTTTCCCTCTTTCTTCCTCTAGGGTGTCGTTAA
TTACCCGTACTAAAGGTTTGGAAAAGAAAAAAGAGACCGCCTCGTTTCTTTTTCTTCGTC
GAAAAAGGCAATAAAAATTTTTATCACGTTTCTTTTTCTTGAAATTTTTTTTTTTAGTTTTT
TTCTCTTTCAGTGACCTCCATTGATATTTAAGTTAATAAACGGTCTTCAATTTCTCAAGTT
TCAGTTTCATTTTTCTTGTTCTATTACAACTTTTTTTACTTCTTGTTCATTAGAAAGAAAGC
ATAGCAATCTAATCTAAGGGGCGGTGTTGACAATTAATCATCGGCATAGTATATCGGCA
TAGTATAATACGACAAGGTGAGGAACTAAACCATGGCCAAGTTGACCAGTGCCGTTCC
GGTGCTCACCGCGCGCGACGTCGCCGGAGCGGTCGAGTTCTGGACCGACCGGCTCGGG
TTCTCCCGGGACTTCGTGGAGGACGACTTCGCCGGTGTGGTCCGGGACGACGTGACCCT
GTTCATCAGCGCGGTCCAGGACCAGGTGGTGCCGGACAACACCCTGGCCTGGGTGTGG
GTGCGCGGCCTGGACGAGCTGTACGCCGAGTGGTCGGAGGTCGTGTCCACGAACTTCC
GGGACGCCTCCGGGCCGGCCATGACCGAGATCGGCGAGCAGCCGTGGGGGCGGGAGT
TCGCCCTGCGCGACCCGGCCGGCAACTGCGTGCACTTCGTGGCCGAGGAGCAGGACTG
ACACGTCCGACGGCGGCCCACGGGTCCCAGGCCTCGGAGATCCGTCCCCCTTTTCCTTT
GTCGATATCATGTAATTAGTTATGTCACGCTTACATTCACGCCCTCCCCCCACATCCGCT
CTAACCGAAAAGGAAGGAGTTAGACAACCTGAAGTCTAGGTCCCTATTTATTTTTTTAT
AGTTATGTTAGTATTAAGAACGTTATTTATATTTCAAATTTTTCTTTTTTTTCTGTACAGAC
GCGGTACCCAGCTTTTGTTCCCTTTAGTGAGGGTTAATTCCGAGCTTGGCGTAATCATGG
TCATAGCTGTTTCCTGTGTGAAATTGTTATCCGCTCACAATTCCACACAACATACGAGCC
GGAAGCATAAAGTGTAAAGCCTGGGGTGCCTAATGAGTGAGCTAACTCACATTAATTG
CGTTGCGCTCACTGCCCGCTTTCCAGTCGGGAAACCTGTCGTGCCAGCTGCATTAATGA
ATCGGCCAACGCGCGGGGAGAGGCGGTTTGCGTATTGGGCGCTCTTCCGCTTCCTCGCT

CACTGACTCGCTGCGCTCGGTCGTTCGGCTGCGGCGAGCGGTATCAGCTCACTCAAAGG
CGGTAATACGGTTATCCACAGAATCAGGGGATAACGCAGGAAAGAACATGTGAGCAA
AAGGCCAGCAAAAGGCCAGGAACCGTAAAAAGGCCGCGTTGCTGGCGTTTTTCCATAG
GCTCCGCCCCCCTGACGAGCATCACAAAAATCGACGCTCAAGTCAGAGGTGGCGAAAC
CCGACAGGACTATAAAGATACCAGGCGTTTCCCCCTGGAAGCTCCCTCGTGCGCTCTCC
TGTTCCGACCCTGCCGCTTACCGGATACCTGTCCGCCTTTCTCCCTTCGGGAAGCGTGGC
GCTTTCTCATAGCTCACGCTGTAGGTATCTCAGTTCGGTGTAGGTCGTTCGCTCCAAGCT
GGGCTGTGTGCACGAACCCCCCGTTCAGCCCGACCGCTGCGCCTTATCCGGTAACTATC
GTCTTGAGTCCAACCCGGTAAGACACGACTTATCGCCACTGGCAGCAGCCACTGGTAA
CAGGATTAGCAGAGCGAGGTATGTAGGCGGTGCTACAGAGTTCTTGAAGTGGTGGCCT
AACTACGGCTACACTAGAAGGACAGTATTTGGTATCTGCGCTCTGCTGAAGCCAGTTAC
CTTCGGAAAAAGAGTTGGTAGCTCTTGATCCGGCAAACAAACCACCGCTGGTAGCGGT
GGTTTTTTTGTTTGCAAGCAGCAGATTACGCGCAGAAAAAAAGGATCTCAAGAAGATC
CTTTGATCTTTTCTACGGGGTCTGACGCTCAGTGGAACGAAAACTCACGTTAAGGGATTT
TGGTCATGAGATTATCAAAAAGGATCTTCACCTAGATCCTTTTAAATTAAAAATGAAGT
TTTAAATCAATCTAAAGTATATATGAGTAAACTTGGTCTGACAGTTACCAATGCTTAATC
AGTGAGGCACCTATCTCAGCGATCTGTCTATTTCGTTCATCCATAGTTGCCTGACTCCCC
GTCGTGTAGATAACTACGATACGGGAGGGCTTACCATCTGGCCCCAGTGCTGCAATGAT
ACCGCGAGACCCACGCTCACCGGCTCCAGATTTATCAGCAATAAACCAGCCAGCCGGA
AGGGCCGAGCGCAGAAGTGGTCCTGCAACTTTATCCGCCTCCATCCAGTCTATTAATTG
TTGCCGGGAAGCTAGAGTAAGTAGTTCGCCAGTTAATAGTTTGCGCAACGTTGTTGCCA
TTGCTACAGGCATCGTGGTGTCACGCTCGTCGTTTGGTATGGCTTCATTCAGCTCCGGTT
CCCAACGATCAAGGCGAGTTACATGATCCCCCATGTTGTGCAAAAAAGCGGTTAGCTC
CTTCGGTCCTCCGATCGTTGTCAGAAGTAAGTTGGCCGCAGTGTTATCACTCATGGTTAT
GGCAGCACTGCATAATTCTCTTACTGTCATGCCATCCGTAAGATGCTTTTCTGTGACTGG
TGAGTACTCAACCAAGTCATTCTGAGAATAGTGTATGCGGCGACCGAGTTGCTCTTGCC
CGGCGTCAATACGGGATAATACCGCGCCACATAGCAGAACTTTAAAAGTGCTCATCAT
TGGAAAACGTTCTTCGGGGCGAAAACTCTCAAGGATCTTACCGCTGTTGAGATCCAGTT
CGATGTAACCCACTCGTGCACCCAACTGATCTTCAGCATCTTTTACTTTCACCAGCGTTT
CTGGGTGAGCAAAAACAGGAAGGCAAAATGCCGCAAAAAAGGGAATAAGGGCGACA
CGGAAATGTTGAATACTCATACTCTTCCTTTTTCAATATTATTGAAGCATTTATCAGGGTT
ATTGTCTCATGAGCGGATACATATTTGAATGTATTTAGAAAAATAAACAAATAGGGGTT
CCGCGCACATTTCCCCGAAAAGTGCCACCTGGGAAATTGTAAACGTTAATATTTTGTTA
AAATTCGCGTTAAATTTTTGTTAAATCAGCTCATTTTTTAACCAATAGGCCGAAATCGGC
AAAATCCCTTATAAATCAAAAGAATAGACCGAGATAGGGTTGAGTGTTGTTCCAGTTTG
GAACAAGAGTCCACTATTAAAGAACGTGGACTCCAACGTCAAAGGGCGAAAAACCGT
CTATCAGGGCGATGGCCCACTACGTGAACCATCACCCTAATCAAGTTTTTTGGGGTCGA

GGTGCCGTAAAGCACTAAATCGGAACCCTAAAGGGAGCCCCCGATTTAGAGCTTGACG
GGGAAAGCCGGCGAACGTGGCGAGAAAGGAAGGGAAGAAAGCGAAAGGAGCGGGCG
CTAGGGCGCTGGCAAGTGTAGCGGTCACGCTGCGCGTAACCACCACACCCGCCGCGCT
TAATGCGCCGCTACAGGGCGCGTCGCGCCATTCGCCATTCAGGCTGCGCAACTGTTGGG
AAGGGCGATCGGTGCGGGCCTCTTCGCTATTACGCCAGCTGGCGAAAGGGGGATGTGC
TGCAAGGCGATTAAGTTGGGTAACGCCAGGGTTTTCCCAGTCACGACGTTGTAAAACGA
CGGCCAGTGAATTGTAATACGACTCACTATAGGGCGAATTGGAGCTCCACCGCGGTGG
CGGCCGCCCCCTGCATTATTAATCACCACCCCGTCTACGATGACAGGCTCGCGACTGCA
GAGATGGCCTTTGTTACGGGCAATACATTTGTCACACGGCACCTCGAAGTTGCACTTAA
CTTTGCGCTTCTTGCAGCTGAGACATGTCTTGGAAATACGAGTAGTACGCCGTAGCTTG
GTTATGTCGAGCCTAGTAAACTCCGCAGGAGAATTCCTCTTCTCCGAAGGCACTCTCTC
CGCAGAAGCCATAAATTATTTGAAATCAGAAATGTGAGCGATATAAACACCCCTGCAC
GGGGCTGCACCCCGATTTTTTTCGGGTGATCGTATGATGCTACTATGAGCCCGCAGATA
ACATCTGACTTTACATTAAGGCAAAATTCTGGTGTAGGGTGTCCGCAGGCCGAGTAATG
CTGACCGGTACGCACCTTTTTCGTCCGTTGTGGTGTGCTGCTTCATGGTCAATTTTTTTA
GCTCAGATGACATCGGAAGGTGTTCCTACAAAGCACACCTCCAATTCGCCGTAAAACCT
CGGAGTAAATCATTCTCCCTGTAGTGTAAACTAAGCTGATGAGGCGTTCGGCAGATCCA
ATTGGACACACCCCGTCATGGGGTGTGAAATACCCCGCCACGGCCATCCGGCAGCCCC
ACTCCACCACTTTGGGTTGAATATGGTCCAATTGTTATGTGCAAATTTTCGGACATTGGA
AATCACCCACTCGGAGTAAAGCAACTCGTAGTGACTGAAAAATAAGACGTCATCATTA
ATTAGGCTGCAACAGACATATAAATACGAGACACTTTTCCCTCTTCTTAGGCATCCTTCT
ATCAATCAATCAATCAATTTAAAAAGCTTATGAATACCTTATCTCAAGCCATCAAGGCA
TACAATTCAAATGACTATCAATTGGCTTTGAAATTGTTCGAAAAGTCAGCAGAAATCTA
CGGACGTAAGATAGTAGAGTTTCAGATTACTAAGTGCAAGGAGAAATTGTCTGCCCATC
CAAGTGTCAATTCAGCTCATCCTTCAGTCAACTCAGCACATTTGTCAGTAAACAAAGAG
GAAAAGGTTAATGTGTGCGATTCTCCATTGGATATTGCTACCCAATTGTTGTTGTCAAAT
GTCAAGAAATTAGTGTTATCTGACAGTGAGAAGAACACATTGAAGAATAAGTGGAAGT
TGTTAACTGAAAAGAAAAGTGAGAATGCTGAAGTTCGTGCTGTAGCATTGGTACCAAA
GGATTTCCCAAAGGATTTGGTGTTGGCACCATTACCTGACCATGTGAATGACTTTACTTG
GTACAAGAAGAGAAAGAAACGTTTAGGTATCAAACCAGAACATCAACACGTGGGTTTG
TCAATAATTGTCACCACTTTCAATCGTCCTGCAATCTTAAGTATAACTTTGGCATGCTTA
GTTAATCAAAAGACTCACTATCCATTCGAGGTGATTGTCACAGATGATGGATCACAAGA
AGATTTGTCTCCAATCATAAGACAATATGAAAACAAATTGGATATCCGTTATGTCAGAC
AAAAGGACAATGGTTTCCAAGCTAGTGCTGCTAGGAATATGGGTTTGAGATTAGCAAA
GTATGATTTCATTGGTTTGTTGGATTGCGATATGGCACCTAACCCATTATGGGTGCATTC
ATATGTCGCTGAATTGTTAGAAGATGATGATTTGACAATCATTGGACCAAGAAAGTACA
TTGATACACAACATATCGACCCAAAGGACTTCTTAAACAATGCATCTTTGTTGGAATCA

TTGCCAGAAGTTAAGACCAATAACTCAGTGGCCGCAAAAGGTGAAGGTACCGTTTCAT
TGGATTGGAGGTTGGAGCAATTCGAAAAGACTGAAAACTTAAGATTGTCAGACTCTCCT
TTTAGATTCTTCGCAGCTGGTAATGTTGCTTTCGCCAAGAAGTGGTTGAACAAATCTGGA
TTCTTTGATGAAGAGTTCAACCATTGGGGTGGTGAAGATGTTGAGTTTGGATATAGATTG
TTTAGGTATGGTTCATTCTTCAAGACTATTGACGGTATCATGGCCTACCATCAAGAGCCA
CCTGGTAAGGAAAACGAAACAGATAGGGAAGCTGGAAAGAACATCACATTGGATATT
ATGAGGGAGAAGGTACCATATATTTACAGGAAGTTGTTGCCTATCGAAGATTCACACAT
CAATAGAGTCCCTTTGGTTTCTATCTATATCCCAGCTTACAACTGTGCCAATTATATTCA
ACGTTGTGTTGATTCTGCCTTGAACCAGACAGTTGTAGATTTGGAAGTCTGTATTTGCAA
TGATGGTTCTACAGATAATACTTTGGAAGTTATCAACAAGTTGTACGGTAACAATCCAA
GAGTCAGAATCATGAGTAAACCAAATGGTGGTATTGCTAGTGCTTCTAATGCAGCAGTG
AGTTTTGCCAAAGGATATTACATAGGTCAATTAGATTCAGATGACTATTTGGAGCCAGA
TGCCGTAGAGTTATGTTTGAAAGAGTTCTTGAAAGACAAAACTTTGGCTTGTGTATATAC
AACAAACAGAAATGTCAATCCTGATGGTTCTTTGATAGCAAATGGTTACAACTGGCCAG
AGTTTAGTAGGGAGAAGTTGACTACTGCAATGATTGCTCATCACTTCCGTATGTTCACTA
TCAGGGCATGGCATTTGACCGATGGTTTTAATGAGAAGATTGAGAATGCTGTGGACTAC
GATATGTTCTTGAAGTTGAGTGAAGTTGGTAAGTTCAAGCACTTAAACAAAATCTGCTA
TAACAGGGTATTGCATGGTGATAATACAAGTATTAAGAAGTTGGGTATCCAAAAGAAG
AACCATTTCGTGGTCGTCAACCAGAGTTTGAACAGGCAAGGAATCACTTACTACAATTA
CGACGAGTTCGATGACTTAGATGAGTCTAGGAAATACATCTTTAACAAAACAGCTGAG
TACCAGGAAGAAATTGACATCTTAAAGGACATTAAGATCATACAAAACAAGGACGCTA
AAATAGCAGTATCTATCTTCTACCCAAATACTTTGAATGGTTTGGTCAAGAAATTGAAT
AACATCATCGAGTACAACAAGAACATATTCGTTATTGTCTTGCATGTGGACAAGAACCA
TTTGACCCCAGATATCAAGAAAGAGATATTGGCTTTCTACCACAAGCATCAAGTGAATA
TTTTGTTGAATAACGATATCTCATACTACACATCAAACCGTTTAATCAAGACCGAGGCA
CATTTATCAAACATTAATAAGTTGTCACAGTTGAACTTGAATTGTGAATATATCATATTC
GACAATCATGACTCTTTGTTCGTGAAGAATGATTCTTATGCCTATATGAAGAAGTACGA
TGTTGGTATGAATTTCTCAGCCTTAACTCATGATTGGATTGAAAAGATTAACGCACATCC
ACCATTCAAGAAGTTGATTAAGACATACTTTAACGATAATGACTTGAAATCTATGAACG
TTAAAGGAGCTAGTCAAGGAATGTTTATGACATATGCATTGGCTCACGAATTGTTGACT
ATTATCAAAGAGGTTATCACTTCTTGCCAATCTATCGATTCTGTACCAGAATACAACACT
GAGGACATATGGTTTCAATTTGCATTGTTGATCTTGGAAAAGAAAACTGGTCATGTCTTT
AACAAGACAAGTACCTTGACATACATGCCTTGGGAGAGGAAGTTGCAATGGACCAATG
AACAAATTGAATCAGCTAAACGTGGAGAAAACATTCCAGTGAACAAGTTCATAATCAA
TTCAATCACATTGTAAAGGCCTAAAGGGGATATCC

Sequence S8. The nucleotide sequence of the pHIPZ18_*hasB* plasmid. The sequence of the *hasB* gene is shown in yellow.

CTAGAGGATCGATCCCCGGGCCTGGACATCCAGCCTTCCTACGCCATGACCACCTCCGA
GGCTAAGAGGGCCGTGCACAAGGAGACCAAGGACAAAACCTCGAGACTTGCCTTTGA
AGGCTCTTGTTGCGGTAAATAAGTATATAGGACACGACAATCTAGTAATCTCCACTATT
GACGAGCTCGTCGAACTGCGAAAATAGGTTTTCCATCTGGTCTGTAGGCATCAGCCCGG
CGTCATCCTCCTGCGCAGGAGCAGCGGGCTCAGGGCCGGCCTGGGCGGGCTGATCCAG
AAAGTCGAGGTTCAGATCCCCCACACACCATAGCTTCAAAATGTTTCTACTCCTTTTTTA
CTCTTCCAGATTTTCTCGGACTCCGCGCATCGCCGTACCACTTCAAAACACCCAAGCAC
AGCATACTAAATTTTCCCTCTTTCTTCCTCTAGGGTGTCGTTAATTACCCGTACTAAAGGT
TTGGAAAAGAAAAAAGAGACCGCCTCGTTTCTTTTTCTTCGTCGAAAAAGGCAATAAA
AATTTTTATCACGTTTCTTTTTCTTGAAATTTTTTTTTTTAGTTTTTTTCTCTTTCAGTGACCT
CCATTGATATTTAAGTTAATAAACGGTCTTCAATTTCTCAAGTTTCAGTTTCATTTTTCTT
GTTCTATTACAACTTTTTTTACTTCTTGTTCATTAGAAAGAAAGCATAGCAATCTAATCT
AAGGGGCGGTGTTGACAATTAATCATCGGCATAGTATATCGGCATAGTATAATACGAC
AAGGTGAGGAACTAAACCATGGCCAAGTTGACCAGTGCCGTTCCGGTGCTCACCGCGC
GCGACGTCGCCGGAGCGGTCGAGTTCTGGACCGACCGGCTCGGGTTCTCCCGGGACTTC
GTGGAGGACGACTTCGCCGGTGTGGTCCGGGACGACGTGACCCTGTTCATCAGCGCGGT
CCAGGACCAGGTGGTGCCGGACAACACCCTGGCCTGGGTGTGGGTGCGCGGCCTGGAC
GAGCTGTACGCCGAGTGGTCGGAGGTCGTGTCCACGAACTTCCGGGACGCCTCCGGGC
CGGCCATGACCGAGATCGGCGAGCAGCCGTGGGGGCGGGAGTTCGCCCTGCGCGACCC
GGCCGGCAACTGCGTGCACTTCGTGGCCGAGGAGCAGGACTGACACGTCCGACGGCGG
CCCACGGGTCCCAGGCCTCGGAGATCCGTCCCCCTTTTCCTTTGTCGATATCATGTAATT
AGTTATGTCACGCTTACATTCACGCCCTCCCCCCACATCCGCTCTAACCGAAAAGGAAG
GAGTTAGACAACCTGAAGTCTAGGTCCCTATTTATTTTTTTATAGTTATGTTAGTATTAAG
AACGTTATTTATATTTCAAATTTTTCTTTTTTTTCTGTACAGACGCGGTACCCAGCTTTTGT
TCCCTTTAGTGAGGGTTAATTCCGAGCTTGGCGTAATCATGGTCATAGCTGTTTCCTGTG
TGAAATTGTTATCCGCTCACAATTCCACACAACATACGAGCCGGAAGCATAAAGTGTA
AAGCCTGGGGTGCCTAATGAGTGAGCTAACTCACATTAATTGCGTTGCGCTCACTGCCC
GCTTTCCAGTCGGGAAACCTGTCGTGCCAGCTGCATTAATGAATCGGCCAACGCGCGGG
GAGAGGCGGTTTGCGTATTGGGCGCTCTTCCGCTTCCTCGCTCACTGACTCGCTGCGCTC
GGTCGTTCGGCTGCGGCGAGCGGTATCAGCTCACTCAAAGGCGGTAATACGGTTATCCA
CAGAATCAGGGGATAACGCAGGAAAGAACATGTGAGCAAAAGGCCAGCAAAAGGCC
AGGAACCGTAAAAAGGCCGCGTTGCTGGCGTTTTTCCATAGGCTCCGCCCCCCTGACGA
GCATCACAAAAATCGACGCTCAAGTCAGAGGTGGCGAAACCCGACAGGACTATAAAG
ATACCAGGCGTTTCCCCCTGGAAGCTCCCTCGTGCGCTCTCCTGTTCCGACCCTGCCGCT
TACCGGATACCTGTCCGCCTTTCTCCCTTCGGGAAGCGTGGCGCTTTCTCATAGCTCACG

CTGTAGGTATCTCAGTTCGGTGTAGGTCGTTCGCTCCAAGCTGGGCTGTGTGCACGAAC
CCCCCGTTCAGCCCGACCGCTGCGCCTTATCCGGTAACTATCGTCTTGAGTCCAACCCG
GTAAGACACGACTTATCGCCACTGGCAGCAGCCACTGGTAACAGGATTAGCAGAGCGA
GGTATGTAGGCGGTGCTACAGAGTTCTTGAAGTGGTGGCCTAACTACGGCTACACTAGA
AGGACAGTATTTGGTATCTGCGCTCTGCTGAAGCCAGTTACCTTCGGAAAAAGAGTTGG
TAGCTCTTGATCCGGCAAACAAACCACCGCTGGTAGCGGTGGTTTTTTTGTTTGCAAGCA
GCAGATTACGCGCAGAAAAAAAGGATCTCAAGAAGATCCTTTGATCTTTTCTACGGGGT
CTGACGCTCAGTGGAACGAAAACTCACGTTAAGGGATTTTGGTCATGAGATTATCAAA
AAGGATCTTCACCTAGATCCTTTTAAATTAAAAATGAAGTTTTAAATCAATCTAAAGTA
TATATGAGTAAACTTGGTCTGACAGTTACCAATGCTTAATCAGTGAGGCACCTATCTCA
GCGATCTGTCTATTTCGTTCATCCATAGTTGCCTGACTCCCCGTCGTGTAGATAACTACG
ATACGGGAGGGCTTACCATCTGGCCCCAGTGCTGCAATGATACCGCGAGACCCACGCT
CACCGGCTCCAGATTTATCAGCAATAAACCAGCCAGCCGGAAGGGCCGAGCGCAGAA
GTGGTCCTGCAACTTTATCCGCCTCCATCCAGTCTATTAATTGTTGCCGGGAAGCTAGAG
TAAGTAGTTCGCCAGTTAATAGTTTGCGCAACGTTGTTGCCATTGCTACAGGCATCGTGG
TGTCACGCTCGTCGTTTGGTATGGCTTCATTCAGCTCCGGTTCCCAACGATCAAGGCGAG
TTACATGATCCCCCATGTTGTGCAAAAAAGCGGTTAGCTCCTTCGGTCCTCCGATCGTTG
TCAGAAGTAAGTTGGCCGCAGTGTTATCACTCATGGTTATGGCAGCACTGCATAATTCT
CTTACTGTCATGCCATCCGTAAGATGCTTTTCTGTGACTGGTGAGTACTCAACCAAGTCA
TTCTGAGAATAGTGTATGCGGCGACCGAGTTGCTCTTGCCCGGCGTCAATACGGGATAA
TACCGCGCCACATAGCAGAACTTTAAAAGTGCTCATCATTGGAAAACGTTCTTCGGGGC
GAAAACTCTCAAGGATCTTACCGCTGTTGAGATCCAGTTCGATGTAACCCACTCGTGCA
CCCAACTGATCTTCAGCATCTTTTACTTTCACCAGCGTTTCTGGGTGAGCAAAAACAGG
AAGGCAAAATGCCGCAAAAAAGGGAATAAGGGCGACACGGAAATGTTGAATACTCAT
ACTCTTCCTTTTTCAATATTATTGAAGCATTTATCAGGGTTATTGTCTCATGAGCGGATAC
ATATTTGAATGTATTTAGAAAAATAAACAAATAGGGGTTCCGCGCACATTTCCCCGAAA
AGTGCCACCTGGGAAATTGTAAACGTTAATATTTTGTTAAAATTCGCGTTAAATTTTTGT
TAAATCAGCTCATTTTTTAACCAATAGGCCGAAATCGGCAAAATCCCTTATAAATCAAA
AGAATAGACCGAGATAGGGTTGAGTGTTGTTCCAGTTTGGAACAAGAGTCCACTATTAA
AGAACGTGGACTCCAACGTCAAAGGGCGAAAAACCGTCTATCAGGGCGATGGCCCACT
ACGTGAACCATCACCCTAATCAAGTTTTTTGGGGTCGAGGTGCCGTAAAGCACTAAATC
GGAACCCTAAAGGGAGCCCCCGATTTAGAGCTTGACGGGGAAAGCCGGCGAACGTGG
CGAGAAAGGAAGGGAAGAAAGCGAAAGGAGCGGGCGCTAGGGCGCTGGCAAGTGTA
GCGGTCACGCTGCGCGTAACCACCACACCCGCCGCGCTTAATGCGCCGCTACAGGGCG
CGTCGCGCCATTCGCCATTCAGGCTGCGCAACTGTTGGGAAGGGCGATCGGTGCGGGCC
TCTTCGCTATTACGCCAGCTGGCGAAAGGGGGATGTGCTGCAAGGCGATTAAGTTGGGT
AACGCCAGGGTTTTCCCAGTCACGACGTTGTAAAACGACGGCCAGTGAATTGTAATAC

GACTCACTATAGGGCGAATTGGAGCTCCACCGCGGTGGCGGCCGCCCCCTGCATTATTA
ATCACCACCCCGTCTACGATGACAGGCTCGCGACTGCAGAGATGGCCTTTGTTACGGGC
AATACATTTGTCACACGGCACCTCGAAGTTGCACTTAACTTTGCGCTTCTTGCAGCTGAG
ACATGTCTTGGAAATACGAGTAGTACGCCGTAGCTTGGTTATGTCGAGCCTAGTAAACT
CCGCAGGAGAATTCCTCTTCTCCGAAGGCACTCTCTCCGCAGAAGCCATAAATTATTTG
AAATCAGAAATGTGAGCGATATAAACACCCCTGCACGGGGCTGCACCCCGATTTTTTTC
GGGTGATCGTATGATGCTACTATGAGCCCGCAGATAACATCTGACTTTACATTAAGGCA
AAATTCTGGTGTAGGGTGTCCGCAGGCCGAGTAATGCTGACCGGTACGCACCTTTTTCG
TCCGTTGTGGTGTGCTGCTTCATGGTCAATTTTTTTTAGCTCAGATGACATCGGAAGGTG
TTCCTACAAAGCACACCTCCAATTCGCCGTAAAACCTCGGAGTAAATCATTCTCCCTGT
AGTGTAAACTAAGCTGATGAGGCGTTCGGCAGATCCAATTGGACACACCCCGTCATGG
GGTGTGAAATACCCCGCCACGGCCATCCGGCAGCCCCACTCCACCACTTTGGGTTGAAT
ATGGTCCAATTGTTATGTGCAAATTTTCGGACATTGGAAATCACCCACTCGGAGTAAAG
CAACTCGTAGTGACTGAAAAATAAGACGTCATCATTAATTAGGCTGCAACAGACATAT
AAATACGAGACACTTTTCCCTCTTCTTAGGCATCCTTCTATCAATCAATCAATCAATTTA
AAAAGCTTAATGTTTCAGATCAAGAAGATTTGTTGTATTGGTGCCGGTTACGTCGGTGGT
CCAACCTGTTCTGTCATTGCACAGATGTGCCCTGACATTAAGGTCACTGTTGTGGATGTG
AACCAAGCCAGGATCAATGCTTGGAATAGTGACACTTTGCCTATCTACGAACCAGGTTT
GAAGGAAGTCGTAGAGTCATGCAGGGGAAAGAATTTGTTCTACTCAACTGACATTGAT
GGTGCAATTCAAGAAGCTGATTTGGTGTTCATCTCAGTCAACACTCCAACAAAAACTTA
CGGTATGGGTAAGGGAAGGGCAGCCGACTTGAAATACATTGAGGCTTGCGCTAGAAGA
ATAGTACAGAATAGTAACGGATACAAGATTGTTACAGAGAAATCTACTGTGCCAGTTA
GAGCTGCTGAATCAATAAGACGTATCTTCGATGCAAATACTAAACCAGATTTGAACTTG
CAGGTATTGAGTAACCCAGAGTTTTTGGCAGAGGGTACAGCCATTAAGGATTTGAAGA
ACCCTGATAGAGTTTTGATAGGTGGTGACGAAACCCCTGAAGGTCAGAAAGCTGTTAG
AGCTTTGTGTGACGTATACGAACACTGGGTACCATCTGAGAAAATCATAACCACAAAC
ACCTGGTCTTCTGAGTTGAGTAAGTTAGCAGCCAACGCATTCTTAGCTCAAAGAATTTCT
TCAATCAACTCAATTAGTGCCTTATGTGAAGCCACAGGAGCTGACGTTGAAGAGGTTGC
CAGAGCTATTGGTATGGATCAAAGAATTGGTAACAAGTTTTTGAAGGCTTCAGTGGGAT
TTGGAGGTTCATGTTTTCAGAAGGACGTTTTGAACTTGGTTTACTTGTGTGAGGTGTTAA
ACTTGCACGAAGTGGCCAAGTACTGGCAACAAGTGATTGATATGAATGATTATCAAAG
GAGACGTTTTACAACTAGGATAATCGATTGTTTGTTTAACACCGTGACCGATAAGAAAA
TCGCATTGTTAGGTTTCGCTTTCAAGAAGGATACAGGTGATACTAGAGAGAGTAGTTCA
ATCTATATCTCTAAGTATTTGATGGATGAAGGTGCTAAGTTACATATCTACGATCCAAA
GGTCCCACGTGAGCAGATCATCACTGACTTGAGTCAACCTGGTGTTGCAGCTGACGACA
GGGTTTCTCAATTGGTCCACATAAGTACAGATTTGTACGAAGCCTGTGAGAATGCACAC
GCTATGGTCATTTGTACTGAATGGGATATGTTCAAGGAATTAGATTTCAATAGAATCCA

TAGGATGATGTTAAAGCCTGCTTTCATATTCGATGGTAGACGTGTTTTAGATGAATTGCA
TGGAGAATTGCAAAACATTGGATTTCAGGTGGAAACCATCGGAAAGAAGGTAGCTTCA
AAAAGAATACCATTCACTCCAACTGCTGATATCCCTAAGTTCGGTTTACAGGACTTGCC
ACACAAGAAGCAACGTGTGTAAGTCGACT

Sequence S9. The nucleotide sequence of the pHIPH4_*hasB* plasmid. The sequence of the *hasB* gene is shown in yellow.

AAGCTTAATGTTTCAGATCAAGAAGATTTGTTGTATTGGTGCCGGTTACGTCGGTGGTCC
AACCTGTTCTGTCATTGCACAGATGTGCCCTGACATTAAGGTCACTGTTGTGGATGTGAA
CCAAGCCAGGATCAATGCTTGGAATAGTGACACTTTGCCTATCTACGAACCAGGTTTGA
AGGAAGTCGTAGAGTCATGCAGGGGAAAGAATTTGTTCTACTCAACTGACATTGATGGT
GCAATTCAAGAAGCTGATTTGGTGTTCATCTCAGTCAACACTCCAACAAAAACTTACGG
TATGGGTAAGGGAAGGGCAGCCGACTTGAAATACATTGAGGCTTGCGCTAGAAGAATA
GTACAGAATAGTAACGGATACAAGATTGTTACAGAGAAATCTACTGTGCCAGTTAGAG
CTGCTGAATCAATAAGACGTATCTTCGATGCAAATACTAAACCAGATTTGAACTTGCAG
GTATTGAGTAACCCAGAGTTTTTGGCAGAGGGTACAGCCATTAAGGATTTGAAGAACCC
TGATAGAGTTTTGATAGGTGGTGACGAAACCCCTGAAGGTCAGAAAGCTGTTAGAGCTT
TGTGTGACGTATACGAACACTGGGTACCATCTGAGAAAATCATAACCACAAACACCTG
GTCTTCTGAGTTGAGTAAGTTAGCAGCCAACGCATTCTTAGCTCAAAGAATTTCTTCAAT
CAACTCAATTAGTGCCTTATGTGAAGCCACAGGAGCTGACGTTGAAGAGGTTGCCAGA
GCTATTGGTATGGATCAAAGAATTGGTAACAAGTTTTTGAAGGCTTCAGTGGGATTTGG
AGGTTCATGTTTTCAGAAGGACGTTTTGAACTTGGTTTACTTGTGTGAGGTGTTAAACTT
GCACGAAGTGGCCAAGTACTGGCAACAAGTGATTGATATGAATGATTATCAAAGGAGA
CGTTTTACAACTAGGATAATCGATTGTTTGTTTAACACCGTGACCGATAAGAAAATCGC
ATTGTTAGGTTTCGCTTTCAAGAAGGATACAGGTGATACTAGAGAGAGTAGTTCAATCT
ATATCTCTAAGTATTTGATGGATGAAGGTGCTAAGTTACATATCTACGATCCAAAGGTC
CCACGTGAGCAGATCATCACTGACTTGAGTCAACCTGGTGTTGCAGCTGACGACAGGGT
TTCTCAATTGGTCCACATAAGTACAGATTTGTACGAAGCCTGTGAGAATGCACACGCTA
TGGTCATTTGTACTGAATGGGATATGTTCAAGGAATTAGATTTCAATAGAATCCATAGG
ATGATGTTAAAGCCTGCTTTCATATTCGATGGTAGACGTGTTTTAGATGAATTGCATGGA
GAATTGCAAAACATTGGATTTCAGGTGGAAACCATCGGAAAGAAGGTAGCTTCAAAAA
GAATACCATTCACTCCAACTGCTGATATCCCTAAGTTCGGTTTACAGGACTTGCCACAC
AAGAAGCAACGTGTGTAAGTCGACTCTAGAGGATCGATCCCCGGGCCTGGACATCCAG
CCTTCCTACGCCATGACCACCTCCGAGGCTAAGAGGGCCGTGCACAAGGAGACCAAGG
ACAAAACCTCGAGACTTGCCTTTGAAGGCTCTTGTTGCGGTAAATAAGTATATAGGACA
CGACAATCTAGTAATCTCCACTATTGACGAGCTCGTCGAACTGCGAAAATAGGTTTTCC
ATCTGGTCTGTAGGCATCAGCCCGGCGTCATCCTCCTGCGCAGGAGCAGCGGGCTCAGG

GCCGGCCTGGGCGGGCTGATCCAGAAAGTCGAGGTTCAGATCCCCCACACACCATAGC
TTCAAAATGTTTCTACTCCTTTTTTACTCTTCCAGATTTTCTCGGACTCCGCGCATCGCCG
TACCACTTCAAAACACCCAAGCACAGCATACTAAATTTTCCCTCTTTCTTCCTCTAGGGT
GTCGTTAATTACCCGTACTAAAGGTTTGGAAAAGAAAAAAGAGACCGCCTCGTTTCTTT
TTCTTCGTCGAAAAAGGCAATAAAAATTTTTATCACGTTTCTTTTTCTTGAAATTTTTTTTT
TTAGTTTTTTTCTCTTTCAGTGACCTCCATTGATATTTAAGTTAATAAACGGTCTTCAATTT
CTCAAGTTTCAGTTTCATTTTTCTTGTTCTATTACAACTTTTTTTACTTCTTGTTCATTAGA
AAGAAAGCATAGCAATCTAATCTAAGGGGCGGTGTTGACAATTAATCATCGGCATAGT
ATATCGGCATAGTATAATACGACAAGGTGAGGAACTAAACCATGGGTAAAAAGCCTGA
ACTCACCGCGACGTCTGTCGAGAAGTTTCTGATCGAAAAGTTCGACAGCGTCTCCGACC
TGATGCAGCTCTCGGAGGGCGAAGAATCTCGTGCTTTCAGCTTCGATGTAGGAGGGCGT
GGATATGTCCTGCGGGTAAATAGCTGCGCCGATGGTTTCTACAAAGATCGTTATGTTTAT
CGGCACTTTGCATCGGCCGCGCTCCCGATTCCGGAAGTGCTTGACATTGGGGAATTCAG
CGAGAGCCTGACCTATTGCATCTCCCGCCGTGCACAGGGTGTCACGTTGCAAGACCTGC
CTGAAACCGAACTGCCCGCTGTTCTGCAGCCGGTCGCGGAGGCCATGGATGCGATCGCT
GCGGCCGATCTTAGCCAGACGAGCGGGTTCGGCCCATTCGGACCGCAAGGAATCGGTC
AATACACTACATGGCGTGATTTCATATGCGCGATTGCTGATCCCCATGTGTATCACTGGC
AAACTGTGATGGACGACACCGTCAGTGCGTCCGTCGCGCAGGCTCTCGATGAGCTGAT
GCTTTGGGCCGAGGACTGCCCCGAAGTCCGGCACCTCGTGCACGCGGATTTCGGCTCCA
ACAATGTCCTGACGGACAATGGCCGCATAACAGCGGTCATTGACTGGAGCGAGGCGAT
GTTCGGGGATTCCCAATACGAGGTCGCCAACATCTTCTTCTGGAGGCCGTGGTTGGCTT
GTATGGAGCAGCAGACGCGCTACTTCGAGCGGAGGCATCCGGAGCTTGCAGGATCGCC
GCGGCTCCGGGCGTATATGCTCCGCATTGGTCTTGACCAACTCTATCAGAGCTTGGTTGA
CGGCAATTTCGATGATGCAGCTTGGGCGCAGGGTCGATGCGACGCAATCGTCCGATCCG
GAGCCGGGACTGTCGGGCGTACACAAATCGCCCGCAGAAGCGCGGCCGTCTGGACCGA
TGGCTGTGTAGAAGTACTCGCCGATAGTGGAAACCGACGCCCCAGCACTCGTCCGAGG
GCAAAGGAATAATCAGTACTGACAATAAAAAGATTCTTGTTTTCAAGAACTTGTCATTT
GTATAGTTTTTTTATATTGTAGTTGTTCTATTTTAATCAAATGTTAGCGTGATTTATATTTT
TTTTCGCCTCGACATCATCTGCCCAGATGCGAAGTTAAGTGCGCAGAAAGTAATATCAT
GCGTCAATCGTATGTGAATGCTGGTCGCTATACTGCTGTCGATTCGATACTAACGCCGC
CATCCAGTGTCGAAAACGAGCTCGAATTCATCGATGATGTACCCAGCTTTTGTTCCCTTT
AGTGAGGGTTAATTCCGAGCTTGGCGTAATCATGGTCATAGCTGTTTCCTGTGTGAAATT
GTTATCCGCTCACAATTCCACACAACATACGAGCCGGAAGCATAAAGTGTAAAGCCTG
GGGTGCCTAATGAGTGAGCTAACTCACATTAATTGCGTTGCGCTCACTGCCCGCTTTCCA
GTCGGGAAACCTGTCGTGCCAGCTGCATTAATGAATCGGCCAACGCGCGGGGAGAGGC
GGTTTGCGTATTGGGCGCTCTTCCGCTTCCTCGCTCACTGACTCGCTGCGCTCGGTCGTTC
GGCTGCGGCGAGCGGTATCAGCTCACTCAAAGGCGGTAATACGGTTATCCACAGAATC

AGGGGATAACGCAGGAAAGAACATGTGAGCAAAAGGCCAGCAAAAGGCCAGGAACC
GTAAAAAGGCCGCGTTGCTGGCGTTTTTCCATAGGCTCCGCCCCCCTGACGAGCATCAC
AAAAATCGACGCTCAAGTCAGAGGTGGCGAAACCCGACAGGACTATAAAGATACCAG
GCGTTTCCCCCTGGAAGCTCCCTCGTGCGCTCTCCTGTTCCGACCCTGCCGCTTACCGGA
TACCTGTCCGCCTTTCTCCCTTCGGGAAGCGTGGCGCTTTCTCATAGCTCACGCTGTAGG
TATCTCAGTTCGGTGTAGGTCGTTCGCTCCAAGCTGGGCTGTGTGCACGAACCCCCCGTT
CAGCCCGACCGCTGCGCCTTATCCGGTAACTATCGTCTTGAGTCCAACCCGGTAAGACA
CGACTTATCGCCACTGGCAGCAGCCACTGGTAACAGGATTAGCAGAGCGAGGTATGTA
GGCGGTGCTACAGAGTTCTTGAAGTGGTGGCCTAACTACGGCTACACTAGAAGGACAG
TATTTGGTATCTGCGCTCTGCTGAAGCCAGTTACCTTCGGAAAAAGAGTTGGTAGCTCTT
GATCCGGCAAACAAACCACCGCTGGTAGCGGTGGTTTTTTTGTTTGCAAGCAGCAGATT
ACGCGCAGAAAAAAAGGATCTCAAGAAGATCCTTTGATCTTTTCTACGGGGTCTGACGC
TCAGTGGAACGAAAACTCACGTTAAGGGATTTTGGTCATGAGATTATCAAAAAGGATCT
TCACCTAGATCCTTTTAAATTAAAAATGAAGTTTTAAATCAATCTAAAGTATATATGAGT
AAACTTGGTCTGACAGTTACCAATGCTTAATCAGTGAGGCACCTATCTCAGCGATCTGT
CTATTTCGTTCATCCATAGTTGCCTGACTCCCCGTCGTGTAGATAACTACGATACGGGAG
GGCTTACCATCTGGCCCCAGTGCTGCAATGATACCGCGAGACCCACGCTCACCGGCTCC
AGATTTATCAGCAATAAACCAGCCAGCCGGAAGGGCCGAGCGCAGAAGTGGTCCTGCA
ACTTTATCCGCCTCCATCCAGTCTATTAATTGTTGCCGGGAAGCTAGAGTAAGTAGTTCG
CCAGTTAATAGTTTGCGCAACGTTGTTGCCATTGCTACAGGCATCGTGGTGTCACGCTCG
TCGTTTGGTATGGCTTCATTCAGCTCCGGTTCCCAACGATCAAGGCGAGTTACATGATCC
CCCATGTTGTGCAAAAAAGCGGTTAGCTCCTTCGGTCCTCCGATCGTTGTCAGAAGTAA
GTTGGCCGCAGTGTTATCACTCATGGTTATGGCAGCACTGCATAATTCTCTTACTGTCAT
GCCATCCGTAAGATGCTTTTCTGTGACTGGTGAGTACTCAACCAAGTCATTCTGAGAAT
AGTGTATGCGGCGACCGAGTTGCTCTTGCCCGGCGTCAATACGGGATAATACCGCGCCA
CATAGCAGAACTTTAAAAGTGCTCATCATTGGAAAACGTTCTTCGGGGCGAAAACTCTC
AAGGATCTTACCGCTGTTGAGATCCAGTTCGATGTAACCCACTCGTGCACCCAACTGAT
CTTCAGCATCTTTTACTTTCACCAGCGTTTCTGGGTGAGCAAAAACAGGAAGGCAAAAT
GCCGCAAAAAAGGGAATAAGGGCGACACGGAAATGTTGAATACTCATACTCTTCCTTT
TTCAATATTATTGAAGCATTTATCAGGGTTATTGTCTCATGAGCGGATACATATTTGAAT
GTATTTAGAAAAATAAACAAATAGGGGTTCCGCGCACATTTCCCCGAAAAGTGCCACC
TGGGAAATTGTAAACGTTAATATTTTGTTAAAATTCGCGTTAAATTTTTGTTAAATCAGC
TCATTTTTTAACCAATAGGCCGAAATCGGCAAAATCCCTTATAAATCAAAAGAATAGAC
CGAGATAGGGTTGAGTGTTGTTCCAGTTTGGAACAAGAGTCCACTATTAAAGAACGTGG
ACTCCAACGTCAAAGGGCGAAAAACCGTCTATCAGGGCGATGGCCCACTACGTGAACC
ATCACCCTAATCAAGTTTTTTGGGGTCGAGGTGCCGTAAAGCACTAAATCGGAACCCTA
AAGGGAGCCCCCGATTTAGAGCTTGACGGGGAAAGCCGGCGAACGTGGCGAGAAAGG

AAGGGAAGAAAGCGAAAGGAGCGGGCGCTAGGGCGCTGGCAAGTGTAGCGGTCACGC
TGCGCGTAACCACCACACCCGCCGCGCTTAATGCGCCGCTACAGGGCGCGTCGCGCCA
TTCGCCATTCAGGCTGCGCAACTGTTGGGAAGGGCGATCGGTGCGGGCCTCTTCGCTAT
TACGCCAGCTGGCGAAAGGGGGATGTGCTGCAAGGCGATTAAGTTGGGTAACGCCAGG
GTTTTCCCAGTCACGACGTTGTAAAACGACGGCCAGTGAATTGTAATACGACTCACTAT
AGGGCGAATTGGAGCTCCACCGCGGTGGCGGCCGCCACGGCGATATCGGATCTCGACG
CGGAGAACGATCTCCTCGAGCTGCTCGCGGATCAGCTTGTGGCCCGGTAATGGAACCA
GGCCGACGCGACGCTCCTTGCGGACCACGGTGGCTGGCGAGCCCAGTTTGTGAACGAG
GTCGTTTAGAACGTCCTGCGCAAAGTCCAGTGTCAGATGAATGTCCTCCTCGGACCAAT
TCAGCATGTTCTCGAGCAGCCATCTGTCTTTGGAGTAGAAGCGTAATCTCTGCTCCTCGT
TACTGTACCGGAAGAGGTAGTTTGCCTCGCCGCCCATAATGAACAGGTTCTCTTTCTGGT
GGCCTGTGAGCAGCGGGGACGTCTGGACGGCGTCGATGAGGCCCTTGAGGCGCTCGTA
GTACTTGTTCCGTCGCTGTAGCCGGCCGCGGTGACGATACCCACATAGAGGTCCTTGGC
CATTAGTTTGATGAGGTGGGGCAGGATGGGCGACTCGGCATCGAAATTTTTGCCGTCGT
CGTACAGTGTGATGTCACCATCGAATGTAATGAGCTGCAGCTTGCGATCTCGGATGGTT
TTGGAATGGAAGAACCGCGACATCTCCAACAGCTGGGCCGTGTTGAGAATGAGCCGGA
CGTCGTTGAACGAGGGGGCCACAAGCCGGCGTTTGCTGATGGCGCGGCGCTCGTCCTCG
ATGTAGAAGGCCTTTTCCAGAGGCAGTCTCGTGAAGAAGCTGCCAACGCTCGGAACCA
GCTGCACGAGCCGAGACAATTCGGGGGTGCCGGCTTTGGTCATTTCAATGTTGTCGTCG
ATGAGGAGTTCGAGGTCGTGGAAGATTTCCGCGTAGCGGCGTTTTGCCTCAGAGTTTAC
CATGAGGTCGTCCACTGCAGAGATGCCGTTGCTCTTCACCGCGTACAGGACGAACGGC
GTGGCCAGCAGGCCCTTGATCCATTCTATGAGGCCATCTCGACGGTGTTCCTTGAGTGC
GTACTCCACTCTGTAGCGACTGGACATCTCGAGACTGGGCTTGCTGTGCTGGATGCACC
AATTAATTGTTGCCGCATGCATCCTTGCACCGCAAGTTTTTAAAACCCACTCGCTTTAGC
CGTCGCGTAAAACTTGTGAATCTGGCAACTGAGGGGGTTCTGCAGCCGCAACCGAACTT
TTCGCTTCGAGGACGCAGCTGGATGGTGTCATGTGAGGCTCTGTTTGCTGGCGTAGCCTA
CAACGTGACCTTGCCTAACCGGACGGCGCTACCCACTGCTGTCTGTGCCTGCTACCAGA
AAATCACCAGAGCAGCAGAGGGCCGATGTGGCAACTGGTGGGGTGTCGGACAGGCTGT
TTCTCCACAGTGCAAATGCGGGTGAACCGGCCAGAAAGTAAATTCTTATGCTACCGTGC
AGTGACTCCGACATCCCCAGTTTTTGCCCTACTTGATCACAGATGGGGTCAGCGCTGCC
GCTAAGTGTACCCAACCGTCCCCACACGGTCCATCTATAAATACTGCTGCCAGTGCACG
GTGGTGACATCAATCTAAAGTACAAAAAC

Sequence S10. The nucleotide sequence of the pHIPH4_*hasAs* plasmid. The sequence of the *hasAs* gene is shown in blue.

AAGCTTAAAAATGAGAACTTTGAAAAATTTAATTACAGTTGTTGCATTCTCAATTTTCTG
GGTTTTATTAATTTATGTTAATGTTTATTTGTTCGGTGCTAAAGGTTCATTGTCAATTTATG

GTTTCTTATTAATTGCATATTTGTTAGTTAAAATGTCTTTGTCATTCTTCTATAAACCATTC
AAAGGTAGAGCAGGTCAATATAAAGTTGCTGCAATTATTCCATCATATAATGAAGATG
CTGAATCTTTATTGGAAACTTTGAAATCAGTTCAACAACAAACATATCCATTGGCAGAA
ATTTATGTTGTTGATGATGGTTCAGCCGATGAAACAGGTATTAAAAGAATTGAAGATTA
TGTTAGAGATACTGGTGATTTATCATCTAATGTTATTGTTCATCGTAGCGAAAAAAATCA
AGGTAAAAGACATGCACAAGCATGGGCTTTCGAAAGAAGCGATGCAGATGTTTTCTTG
ACAGTTGATTCAGATACATATATTTATCCAGATGCTTTGGAAGAATTATTGAAAACTTTC
AATGATCCTACAGTTTTCGCAGCTACAGGTCATTTGAATGTTAGAAATAGACAAACTAA
TTTGTTAACAAGATTGACTGATATTAGATATGATAATGCATTCGGTGTTGAAAGAGCAG
CACAATCAGTTACTGGTAATATTTTAGTTTGTTCTGGTCCTTTGTCAGTTTATAGAAGAG
AAGTTGTTGTTCCTAATATTGATAGATATATTAATCAAACTTTCTTAGGTATTCCAGTTTC
AATTGGTGATGATAGATGTTTAACTAATTATGCTACTGATTTGGGTAAAACAGTTTATCA
ATCTACAGCTAAATGTATTACTGATGTTCCAGATAAAATGTCTACATATTTGAAACAAC
AAAATAGATGGAATAAATCATTCTTCAGAGAATCTATTATTTCAGTTAAAAAAATTATG
AATAATCCATTCGTTGCTTTGTGGACTATTTTGGAAGTTTCTATGTTCATGATGTTAGTTT
ATTCAGTTGTTGATTTCTTCGTTGATAATGTTAGAGAGTTCGATTGGTTAAGAGTTTTGGC
ATTCTTGGTTATTATTTTCATTGTTGCTTTGTGTAGAAATATTCATTATATGTTGAAACAT
CCTTTGTCTTTCTTGTTATCTCCTTTCTATGGTGTTTTACATTTATTCGTTTTGCAACCATTG
AAATTGTATTCATTGTTCACTATTAGAAATGCTGATTGGGGTACTAGAAAAAAATTGTTG
TAAAGGCCTGATATCGAATTCCTGCAGCCCGGGGGATCCACTAGTTCTAGAGGATCGAT
CCCCGGGCCTGGACATCCAGCCTTCCTACGCCATGACCACCTCCGAGGCTAAGAGGGC
CGTGCACAAGGAGACCAAGGACAAAACCTCGAGACTTGCCTTTGAAGGCTCTTGTTGC
GGTAAATAAGTATATAGGACACGACAATCTAGTAATCTCCACTATTGACGAGCTCGTCG
AACTGCGAAAATAGGTTTTCCATCTGGTCTGTAGGCATCAGCCCGGCGTCATCCTCCTG
CGCAGGAGCAGCGGGCTCAGGGCCGGCCTGGGCGGGCTGATCCAGAAAGTCGAGGTTC
AGATCCCCCACACACCATAGCTTCAAAATGTTTCTACTCCTTTTTTACTCTTCCAGATTTT
CTCGGACTCCGCGCATCGCCGTACCACTTCAAAACACCCAAGCACAGCATACTAAATTT
TCCCTCTTTCTTCCTCTAGGGTGTCGTTAATTACCCGTACTAAAGGTTTGGAAAAGAAAA
AAGAGACCGCCTCGTTTCTTTTTCTTCGTCGAAAAAGGCAATAAAAATTTTTATCACGTT
TCTTTTTCTTGAAATTTTTTTTTTTTAGTTTTTTTCTCTTTCAGTGACCTCCATTGATATTTAA
GTTAATAAACGGTCTTCAATTTCTCAAGTTTCAGTTTCATTTTTCTTGTTCTATTACAACTT
TTTTTACTTCTTGTTCATTAGAAAGAAAGCATAGCAATCTAATCTAAGGGGCGGTGTTGA
CAATTAATCATCGGCATAGTATATCGGCATAGTATAATACGACAAGGTGAGGAACTAA
ACCATGGGTAAAAAGCCTGAACTCACCGCGACGTCTGTCGAGAAGTTTCTGATCGAAA
AGTTCGACAGCGTCTCCGACCTGATGCAGCTCTCGGAGGGCGAAGAATCTCGTGCTTTC
AGCTTCGATGTAGGAGGGCGTGGATATGTCCTGCGGGTAAATAGCTGCGCCGATGGTTT
CTACAAAGATCGTTATGTTTATCGGCACTTTGCATCGGCCGCGCTCCCGATTCCGGAAGT

GCTTGACATTGGGGAATTCAGCGAGAGCCTGACCTATTGCATCTCCCGCCGTGCACAGG
GTGTCACGTTGCAAGACCTGCCTGAAACCGAACTGCCCGCTGTTCTGCAGCCGGTCGCG
GAGGCCATGGATGCGATCGCTGCGGCCGATCTTAGCCAGACGAGCGGGTTCGGCCCAT
TCGGACCGCAAGGAATCGGTCAATACACTACATGGCGTGATTTCATATGCGCGATTGCT
GATCCCCATGTGTATCACTGGCAAACTGTGATGGACGACACCGTCAGTGCGTCCGTCGC
GCAGGCTCTCGATGAGCTGATGCTTTGGGCCGAGGACTGCCCCGAAGTCCGGCACCTCG
TGCACGCGGATTTCGGCTCCAACAATGTCCTGACGGACAATGGCCGCATAACAGCGGT
CATTGACTGGAGCGAGGCGATGTTCGGGGATTCCCAATACGAGGTCGCCAACATCTTCT
TCTGGAGGCCGTGGTTGGCTTGTATGGAGCAGCAGACGCGCTACTTCGAGCGGAGGCAT
CCGGAGCTTGCAGGATCGCCGCGGCTCCGGGCGTATATGCTCCGCATTGGTCTTGACCA
ACTCTATCAGAGCTTGGTTGACGGCAATTTCGATGATGCAGCTTGGGCGCAGGGTCGAT
GCGACGCAATCGTCCGATCCGGAGCCGGGACTGTCGGGCGTACACAAATCGCCCGCAG
AAGCGCGGCCGTCTGGACCGATGGCTGTGTAGAAGTACTCGCCGATAGTGGAAACCGA
CGCCCCAGCACTCGTCCGAGGGCAAAGGAATAATCAGTACTGACAATAAAAAGATTCT
TGTTTTCAAGAACTTGTCATTTGTATAGTTTTTTTATATTGTAGTTGTTCTATTTTAATCAA
ATGTTAGCGTGATTTATATTTTTTTTCGCCTCGACATCATCTGCCCAGATGCGAAGTTAA
GTGCGCAGAAAGTAATATCATGCGTCAATCGTATGTGAATGCTGGTCGCTATACTGCTG
TCGATTCGATACTAACGCCGCCATCCAGTGTCGAAAACGAGCTCGAATTCATCGATGAT
GTACCCAGCTTTTGTTCCCTTTAGTGAGGGTTAATTCCGAGCTTGGCGTAATCATGGTCA
TAGCTGTTTCCTGTGTGAAATTGTTATCCGCTCACAATTCCACACAACATACGAGCCGG
AAGCATAAAGTGTAAAGCCTGGGGTGCCTAATGAGTGAGCTAACTCACATTAATTGCGT
TGCGCTCACTGCCCGCTTTCCAGTCGGGAAACCTGTCGTGCCAGCTGCATTAATGAATC
GGCCAACGCGCGGGGAGAGGCGGTTTGCGTATTGGGCGCTCTTCCGCTTCCTCGCTCAC
TGACTCGCTGCGCTCGGTCGTTCGGCTGCGGCGAGCGGTATCAGCTCACTCAAAGGCGG
TAATACGGTTATCCACAGAATCAGGGGATAACGCAGGAAAGAACATGTGAGCAAAAG
GCCAGCAAAAGGCCAGGAACCGTAAAAAGGCCGCGTTGCTGGCGTTTTTCCATAGGCT
CCGCCCCCCTGACGAGCATCACAAAAATCGACGCTCAAGTCAGAGGTGGCGAAACCCG
ACAGGACTATAAAGATACCAGGCGTTTCCCCCTGGAAGCTCCCTCGTGCGCTCTCCTGT
TCCGACCCTGCCGCTTACCGGATACCTGTCCGCCTTTCTCCCTTCGGGAAGCGTGGCGCT
TTCTCATAGCTCACGCTGTAGGTATCTCAGTTCGGTGTAGGTCGTTCGCTCCAAGCTGGG
CTGTGTGCACGAACCCCCCGTTCAGCCCGACCGCTGCGCCTTATCCGGTAACTATCGTCT
TGAGTCCAACCCGGTAAGACACGACTTATCGCCACTGGCAGCAGCCACTGGTAACAGG
ATTAGCAGAGCGAGGTATGTAGGCGGTGCTACAGAGTTCTTGAAGTGGTGGCCTAACTA
CGGCTACACTAGAAGGACAGTATTTGGTATCTGCGCTCTGCTGAAGCCAGTTACCTTCG
GAAAAAGAGTTGGTAGCTCTTGATCCGGCAAACAAACCACCGCTGGTAGCGGTGGTTTT
TTTGTTTGCAAGCAGCAGATTACGCGCAGAAAAAAAGGATCTCAAGAAGATCCTTTGA
TCTTTTCTACGGGGTCTGACGCTCAGTGGAACGAAAACTCACGTTAAGGGATTTTGGTC

ATGAGATTATCAAAAAGGATCTTCACCTAGATCCTTTTAAATTAAAAATGAAGTTTTAA
ATCAATCTAAAGTATATATGAGTAAACTTGGTCTGACAGTTACCAATGCTTAATCAGTG
AGGCACCTATCTCAGCGATCTGTCTATTTCGTTCATCCATAGTTGCCTGACTCCCCGTCG
TGTAGATAACTACGATACGGGAGGGCTTACCATCTGGCCCCAGTGCTGCAATGATACCG
CGAGACCCACGCTCACCGGCTCCAGATTTATCAGCAATAAACCAGCCAGCCGGAAGGG
CCGAGCGCAGAAGTGGTCCTGCAACTTTATCCGCCTCCATCCAGTCTATTAATTGTTGCC
GGGAAGCTAGAGTAAGTAGTTCGCCAGTTAATAGTTTGCGCAACGTTGTTGCCATTGCT
ACAGGCATCGTGGTGTCACGCTCGTCGTTTGGTATGGCTTCATTCAGCTCCGGTTCCCAA
CGATCAAGGCGAGTTACATGATCCCCCATGTTGTGCAAAAAAGCGGTTAGCTCCTTCGG
TCCTCCGATCGTTGTCAGAAGTAAGTTGGCCGCAGTGTTATCACTCATGGTTATGGCAGC
ACTGCATAATTCTCTTACTGTCATGCCATCCGTAAGATGCTTTTCTGTGACTGGTGAGTA
CTCAACCAAGTCATTCTGAGAATAGTGTATGCGGCGACCGAGTTGCTCTTGCCCGGCGT
CAATACGGGATAATACCGCGCCACATAGCAGAACTTTAAAAGTGCTCATCATTGGAAA
ACGTTCTTCGGGGCGAAAACTCTCAAGGATCTTACCGCTGTTGAGATCCAGTTCGATGT
AACCCACTCGTGCACCCAACTGATCTTCAGCATCTTTTACTTTCACCAGCGTTTCTGGGT
GAGCAAAAACAGGAAGGCAAAATGCCGCAAAAAAGGGAATAAGGGCGACACGGAAA
TGTTGAATACTCATACTCTTCCTTTTTCAATATTATTGAAGCATTTATCAGGGTTATTGTC
TCATGAGCGGATACATATTTGAATGTATTTAGAAAAATAAACAAATAGGGGTTCCGCGC
ACATTTCCCCGAAAAGTGCCACCTGGGAAATTGTAAACGTTAATATTTTGTTAAAATTC
GCGTTAAATTTTTGTTAAATCAGCTCATTTTTTAACCAATAGGCCGAAATCGGCAAAATC
CCTTATAAATCAAAAGAATAGACCGAGATAGGGTTGAGTGTTGTTCCAGTTTGGAACAA
GAGTCCACTATTAAAGAACGTGGACTCCAACGTCAAAGGGCGAAAAACCGTCTATCAG
GGCGATGGCCCACTACGTGAACCATCACCCTAATCAAGTTTTTTGGGGTCGAGGTGCCG
TAAAGCACTAAATCGGAACCCTAAAGGGAGCCCCCGATTTAGAGCTTGACGGGGAAAG
CCGGCGAACGTGGCGAGAAAGGAAGGGAAGAAAGCGAAAGGAGCGGGCGCTAGGGC
GCTGGCAAGTGTAGCGGTCACGCTGCGCGTAACCACCACACCCGCCGCGCTTAATGCG
CCGCTACAGGGCGCGTCGCGCCATTCGCCATTCAGGCTGCGCAACTGTTGGGAAGGGC
GATCGGTGCGGGCCTCTTCGCTATTACGCCAGCTGGCGAAAGGGGGATGTGCTGCAAG
GCGATTAAGTTGGGTAACGCCAGGGTTTTCCCAGTCACGACGTTGTAAAACGACGGCC
AGTGAATTGTAATACGACTCACTATAGGGCGAATTGGAGCTCCACCGCGGTGGCGGCC
GCCACGGCGATATCGGATCTCGACGCGGAGAACGATCTCCTCGAGCTGCTCGCGGATC
AGCTTGTGGCCCGGTAATGGAACCAGGCCGACGCGACGCTCCTTGCGGACCACGGTGG
CTGGCGAGCCCAGTTTGTGAACGAGGTCGTTTAGAACGTCCTGCGCAAAGTCCAGTGTC
AGATGAATGTCCTCCTCGGACCAATTCAGCATGTTCTCGAGCAGCCATCTGTCTTTGGA
GTAGAAGCGTAATCTCTGCTCCTCGTTACTGTACCGGAAGAGGTAGTTTGCCTCGCCGC
CCATAATGAACAGGTTCTCTTTCTGGTGGCCTGTGAGCAGCGGGGACGTCTGGACGGCG
TCGATGAGGCCCTTGAGGCGCTCGTAGTACTTGTTCCGTCGCTGTAGCCGGCCGCGGTG

ACGATACCCACATAGAGGTCCTTGGCCATTAGTTTGATGAGGTGGGGCAGGATGGGCG
ACTCGGCATCGAAATTTTTGCCGTCGTCGTACAGTGTGATGTCACCATCGAATGTAATG
AGCTGCAGCTTGCGATCTCGGATGGTTTTGGAATGGAAGAACCGCGACATCTCCAACA
GCTGGGCCGTGTTGAGAATGAGCCGGACGTCGTTGAACGAGGGGGCCACAAGCCGGCG
TTTGCTGATGGCGCGGCGCTCGTCCTCGATGTAGAAGGCCTTTTCCAGAGGCAGTCTCGT
GAAGAAGCTGCCAACGCTCGGAACCAGCTGCACGAGCCGAGACAATTCGGGGGTGCC
GGCTTTGGTCATTTCAATGTTGTCGTCGATGAGGAGTTCGAGGTCGTGGAAGATTTCCGC
GTAGCGGCGTTTTGCCTCAGAGTTTACCATGAGGTCGTCCACTGCAGAGATGCCGTTGC
TCTTCACCGCGTACAGGACGAACGGCGTGGCCAGCAGGCCCTTGATCCATTCTATGAGG
CCATCTCGACGGTGTTCCTTGAGTGCGTACTCCACTCTGTAGCGACTGGACATCTCGAG
ACTGGGCTTGCTGTGCTGGATGCACCAATTAATTGTTGCCGCATGCATCCTTGCACCGCA
AGTTTTTAAAACCCACTCGCTTTAGCCGTCGCGTAAAACTTGTGAATCTGGCAACTGAG
GGGGTTCTGCAGCCGCAACCGAACTTTTCGCTTCGAGGACGCAGCTGGATGGTGTCATG
TGAGGCTCTGTTTGCTGGCGTAGCCTACAACGTGACCTTGCCTAACCGGACGGCGCTAC
CCACTGCTGTCTGTGCCTGCTACCAGAAAATCACCAGAGCAGCAGAGGGCCGATGTGG
CAACTGGTGGGGTGTCGGACAGGCTGTTTCTCCACAGTGCAAATGCGGGTGAACCGGC
CAGAAAGTAAATTCTTATGCTACCGTGCAGTGACTCCGACATCCCCAGTTTTTGCCCTAC
TTGATCACAGATGGGGTCAGCGCTGCCGCTAAGTGTACCCAACCGTCCCCACACGGTCC
ATCTATAAATACTGCTGCCAGTGCACGGTGGTGACATCAATCTAAAGTACAAAAAC

Sequence S11. The nucleotide sequence of the pHIPH4_*ScSInt13* plasmid. The sequence of the *hasB* gene is shown in yellow. The reverse sequence of the *hasAp* gene is shown in green. The sequence of the Int13 encoding gene is shown in red.

GGCCGCCCCCTGCATTATTAATCACCACCCCGTCTACGATGACAGGCTCGCGACTGCAG
AGATGGCCTTTGTTACGGGCAATACATTTGTCACACGGCACCTCGAAGTTGCACTTAAC
TTTGCGCTTCTTGCAGCTGAGACATGTCTTGGAAATACGAGTAGTACGCCGTAGCTTGGT
TATGTCGAGCCTAGTAAACTCCGCAGGAGAATTCCTCTTCTCCGAAGGCACTCTCTCCG
CAGAAGCCATAAATTATTTGAAATCAGAAATGTGAGCGATATAAACACCCCTGCACGG
GGCTGCACCCCGATTTTTTTCGGGTGATCGTATGATGCTACTATGAGCCCGCAGATAAC
ATCTGACTTTACATTAAGGCAAAATTCTGGTGTAGGGTGTCCGCAGGCCGAGTAATGCT
GACCGGTACGCACCTTTTTCGTCCGTTGTGGTGTGCTGCTTCATGGTCAATTTTTTTTAGC
TCAGATGACATCGGAAGGTGTTCCTACAAAGCACACCTCCAATTCGCCGTAAAACCTCG
GAGTAAATCATTCTCCCTGTAGTGTAAACTAAGCTGATGAGGCGTTCGGCAGATCCAAT
TGGACACACCCCGTCATGGGGTGTGAAATACCCCGCCACGGCCATCCGGCAGCCCCAC
TCCACCACTTTGGGTTGAATATGGTCCAATTGTTATGTGCAAATTTTCGGACATTGGAAA
TCACCCACTCGGAGTAAAGCAACTCGTAGTGACTGAAAAATAAGACGTCATCATTAAT
TAGGCTGCAACAGACATATAAATACGAGACACTTTTCCCTCTTCTTAGGCATCCTTCTAT
CAATCAATCAATCAATTTAAAAGCATACATTGTTGTTGTTTTTCCAGATCCAGTTGGTCC
TGTAAATATAAGCAATCCATGTGAGTTTACAATGTGATTGAATTGATTATGAACTTGTTC
ACTGGAATGTTTTCTCCACGTTTAGCTGATTCAATTTGTTCATTGGTCCATTGCAACTTCC
TCTCCCAAGGCATGTATGTCAAGGTACTTGTCTTGTTAAAGACATGACCAGTTTTCTTTT

CCAAGATCAACAATGCAAATTGAAACCATATGTCCTCAGTGTTGTATTCTGGTACAGAA
TCGATAGATTGGCAAGAAGTGATAACCTCTTTGATAATAGTCAACAATTCGTGAGCCAA
TGCATATGTCATAAACATTCCTTGACTAGCTCCTTTAACGTTCATAGATTTCAAGTCATT
ATCGTTAAAGTATGTCTTAATCAACTTCTTGAATGGTGGATGTGCGTTAATCTTTTCAAT
CCAATCATGAGTTAAGGCTGAGAAATTCATACCAACATCGTACTTCTTCATATAGGCAT
AAGAATCATTCTTCACGAACAAAGAGTCATGATTGTCGAATATGATATATTCACAATTC
AAGTTCAACTGTGACAACTTATTAATGTTTGATAAATGTGCCTCGGTCTTGATTAAACGG
TTTGATGTGTAGTATGAGATATCGTTATTCAACAAAATATTCACTTGATGCTTGTGGTAG
AAAGCCAATATCTCTTTCTTGATATCTGGGGTCAAATGGTTCTTGTCCACATGCAAGACA
ATAACGAATATGTTCTTGTTGTACTCGATGATGTTATTCAATTTCTTGACCAAACCATTC
AAAGTATTTGGGTAGAAGATAGATACTGCTATTTTAGCGTCCTTGTTTTGTATGATCTTA
ATGTCCTTTAAGATGTCAATTTCTTCCTGGTACTCAGCTGTTTTGTTAAAGATGTATTTCC
TAGACTCATCTAAGTCATCGAACTCGTCGTAATTGTAGTAAGTGATTCCTTGCCTGTTCA
AACTCTGGTTGACGACCACGAAATGGTTCTTCTTTTGGATACCCAACTTCTTAATACTTG
TATTATCACCATGCAATACCCTGTTATAGCAGATTTTGTTTAAGTGCTTGAACTTACCAA
CTTCACTCAACTTCAAGAACATATCGTAGTCCACAGCATTCTCAATCTTCTCATTAAAAC
CATCGGTCAAATGCCATGCCCTGATAGTGAACATACGGAAGTGATGAGCAATCATTGC
AGTAGTCAACTTCTCCCTACTAAACTCTGGCCAGTTGTAACCATTTGCTATCAAAGAAC
CATCAGGATTGACATTTCTGTTTGTTGTATATACACAAGCCAAAGTTTTGTCTTTCAAGA
ACTCTTTCAAACATAACTCTACGGCATCTGGCTCCAAATAGTCATCTGAATCTAATTGAC
CTATGTAATATCCTTTGGCAAAACTCACTGCTGCATTAGAAGCACTAGCAATACCACCA
TTTGGTTTACTCATGATTCTGACTCTTGGATTGTTACCGTACAACTTGTTGATAACTTCCA
AAGTATTATCTGTAGAACCATCATTGCAAATACAGACTTCCAAATCTACAACTGTCTGG
TTCAAGGCAGAATCAACACAACGTTGAATATAATTGGCACAGTTGTAAGCTGGGATAT
AGATAGAAACCAAAGGGACTCTATTGATGTGTGAATCTTCGATAGGCAACAACTTCCTG
TAAATATATGGTACCTTCTCCCTCATAATATCCAATGTGATGTTCTTTCCAGCTTCCCTAT
CTGTTTCGTTTTCCTTACCAGGTGGCTCTTGATGGTAGGCCATGATACCGTCAATAGTCTT
GAAGAATGAACCATACCTAAACAATCTATATCCAAACTCAACATCTTCACCACCCCAA
TGGTTGAACTCTTCATCAAAGAATCCAGATTTGTTCAACCACTTCTTGGCGAAAGCAAC
ATTACCAGCTGCGAAGAATCTAAAAGGAGAGTCTGACAATCTTAAGTTTTCAGTCTTTT
CGAATTGCTCCAACCTCCAATCCAATGAAACGGTACCTTCACCTTTTGCGGCCACTGAG
TTATTGGTCTTAACTTCTGGCAATGATTCCAACAAAGATGCATTGTTTAAGAAGTCCTTT
GGGTCGATATGTTGTGTATCAATGTACTTTCTTGGTCCAATGATTGTCAAATCATCATCTT
CTAACAATTCAGCGACATATGAATGCACCCATAATGGGTTAGGTGCCATATCGCAATCC
AACAAACCAATGAAATCATACTTTGCTAATCTCAAACCCATATTCCTAGCAGCACTAGC
TTGGAAACCATTGTCCTTTTGTCTGACATAACGGATATCCAATTTGTTTTCATATTGTCTT
ATGATTGGAGACAAATCTTCTTGTGATCCATCATCTGTGACAATCACCTCGAATGGATA
GTGAGTCTTTTGATTAACTAAGCATGCCAAAGTTATACTTAAGATTGCAGGACGATTGA
AAGTGGTGACAATTATTGACAAACCCACGTGTTGATGTTCTGGTTTGATACCTAAACGTT
TCTTTCTCTTCTTGTACCAAGTAAAGTCATTCACATGGTCAGGTAATGGTGCCAACACCA
AATCCTTTGGGAAATCCTTTGGTACCAATGCTACAGCACGAACTTCAGCATTCTCACTTT
TCTTTTCAGTTAACAACTTCCACTTATTCTTCAATGTGTTCTTCTCACTGTCAGATAACAC
TAATTTCTTGACATTTGACAACAACAATTGGGTAGCAATATCCAATGGAGAATCGCACA
CATTAACCTTTTCCTCTTTGTTTACTGACAAATGTGCTGAGTTGACTGAAGGATGAGCTG
AATTGACACTTGGATGGGCAGACAATTTCTCCTTGCACTTAGTAATCTGAAACTCTACTA

TCTTACGTCCGTAGATTTCTGCTGACTTTTCGAACAATTTCAAAGCCAATTGATAGTCAT
TTGAATTGTATGCCTTGATGGCTTGAGATAAGGTATTCATAAGCTTAAATATTCGGAGTT
GTATTTATGTTACTAAAACAACTGGTCAAGTTCTACAAATACAACCGTTATTGAAGCTT
GCATGCCTGCAGGTCGACTCTAGAGGATCGATCCCCGGGCCTGGACATCCAGCCTTCCT
ACGCCATGACCACCTCCGAGGCTAAGAGGGCCGTGCACAAGGAGACCAAGGACAAAA
CCTCGAGACTTGCCTTTGAAGGCTCTTGTTGCGGTAAATAAGTATATAGGACACGACAA
TCTAGTAATCTCCACTATTGACGAGCTCGTCGAACTGCGAAAATAGGTTTTCCATCTGGT
CTGTAGGCATCAGCCCGGCGTCATCCTCCTGCGCAGGAGCAGCGGGCTCAGGGCCGGC
CTGGGCGGGCTGATCCAGAAAGTCGAGGTTCAGCAAATTAAAGCCTTCGAGCGTCCCA
AAACCTTCTCAAGCAAGGTTTTCAGTATAATGTTACATGCGTACACGCGTCTGTACAGA
AAAAAAAGAAAAATTTGAAATATAAATAACGTTCTTAATACTAACATAACTATAAAAA
AATAAATAGGGACCTAGACTTCAGGTTGTCTAACTCCTTCCTTTTCGGTTAGAGCGGATG
TGGGGGGAGGGCGTGAATGTAAGCGTGACATAACTAATTACATGACAATAACGGTTGT
ATTTGTAGAACTTGACCAGTTGTTTTAGTAACATAAATACAACTCCGAATAAAAAATGT
TTCAGATCAAGAAGATTTGTTGTATTGGTGCCGGTTACGTCGGTGGTCCAACCTGTTCTG
TCATTGCACAGATGTGCCCTGACATTAAGGTCACTGTTGTGGATGTGAACCAAGCCAGG
ATCAATGCTTGGAATAGTGACACTTTGCCTATCTACGAACCAGGTTTGAAGGAAGTCGT
AGAGTCATGCAGGGGAAAGAATTTGTTCTACTCAACTGACATTGATGGTGCAATTCAAG
AAGCTGATTTGGTGTTCATCTCAGTCAACACTCCAACAAAAACTTACGGTATGGGTAAG
GGAAGGGCAGCCGACTTGAAATACATTGAGGCTTGCGCTAGAAGAATAGTACAGAATA
GTAACGGATACAAGATTGTTACAGAGAAATCTACTGTGCCAGTTAGAGCTGCTGAATC
AATAAGACGTATCTTCGATGCAAATACTAAACCAGATTTGAACTTGCAGGTATTGAGTA
ACCCAGAGTTTTTGGCAGAGGGTACAGCCATTAAGGATTTGAAGAACCCTGATAGAGTT
TTGATAGGTGGTGACGAAACCCCTGAAGGTCAGAAAGCTGTTAGAGCTTTGTGTGACGT
ATACGAACACTGGGTACCATCTGAGAAAATCATAACCACAAACACCTGGTCTTCTGAG
TTGAGTAAGTTAGCAGCCAACGCATTCTTAGCTCAAAGAATTTCTTCAATCAACTCAAT
TAGTGCCTTATGTGAAGCCACAGGAGCTGACGTTGAAGAGGTTGCCAGAGCTATTGGTA
TGGATCAAAGAATTGGTAACAAGTTTTTGAAGGCTTCAGTGGGATTTGGAGGTTCATGT
TTTCAGAAGGACGTTTTGAACTTGGTTTACTTGTGTGAGGTGTTAAACTTGCACGAAGTG
GCCAAGTACTGGCAACAAGTGATTGATATGAATGATTATCAAAGGAGACGTTTTACAA
CTAGGATAATCGATTGTTTGTTTAACACCGTGACCGATAAGAAAATCGCATTGTTAGGT
TTCGCTTTCAAGAAGGATACAGGTGATACTAGAGAGAGTAGTTCAATCTATATCTCTAA
GTATTTGATGGATGAAGGTGCTAAGTTACATATCTACGATCCAAAGGTCCCACGTGAGC
AGATCATCACTGACTTGAGTCAACCTGGTGTTGCAGCTGACGACAGGGTTTCTCAATTG
GTCCACATAAGTACAGATTTGTACGAAGCCTGTGAGAATGCACACGCTATGGTCATTTG
TACTGAATGGGATATGTTCAAGGAATTAGATTTCAATAGAATCCATAGGATGATGTTAA
AGCCTGCTTTCATATTCGATGGTAGACGTGTTTTAGATGAATTGCATGGAGAATTGCAA
AACATTGGATTTCAGGTGGAAACCATCGGAAAGAAGGTAGCTTCAAAAAGAATACCAT
TCACTCCAACTGCTGATATCCCTAAGTTCGGTTTACAGGACTTGCCACACAAGAAGCAA
CGTGTGTAAACTCACATGGATTGCTTATATTTACAGGACCAACTGGATCTGGAAAAACA
ACAACAATGTATGCGGATCCACTAGTTCTAGAATCCGTCGAAACTAAGTTCTGGTGTTT
TAAAACTAAAAAAAAGACTAACTATAAAAGTAGAATTTAAGAAGTTTAAGAAATAGAT
TTACAGAATTACAATCAATACCTACCGTCTTTATATACTTATTAGTCAAGTAGGGGAAT
AATTTCAGGGAACTGGTTTCAACCTTTTTTTTCAGCTTTTCCAAATCAGAGAGAGCAGAA
GGTAATAGAAGGTGTAAGAAAATGAGATAGATACATGCGTGGGTCAATTGCCTTGTGT

CATCATTTACTCCAGGCAGGTTGCATCACTCCATTGAGGTTGTGCCCGTTTTTTGCCTGTT
TGTGCCCCTGTTCTCTGTAGTTGCGCTAAGAGAATGGACCTATGAACTGATGGTTGGTGA
AGAAAACAATATTTTGGTGCTGGGATTCTTTTTTTTTCTGGATGCCAGCTTAAAAAGCGG
GCTCCATTATATTTAGTGGATGCCAGGAATAAACTGTTCACCCAGACACCTACGATGTT
ATATATTCTGTGTAACCCGCCCCCTATTTTGGGCATGTACGGGTTACAGCAGAATTAAA
AGGCTAATTTTTTGACTAAATAAAGTTAGGAAAATCACTACTATTAATTATTTACGTATT
CTTTGAAATGGCAGTATTGATAATGATAAACTCGAGGATCCCCCACACACCATAGCTTC
AAAATGTTTCTACTCCTTTTTTACTCTTCCAGATTTTCTCGGACTCCGCGCATCGCCGTAC
CACTTCAAAACACCCAAGCACAGCATACTAAATTTCCCTCTTTCTTCCTCTAGGGTGTC
GTTAATTACCCGTACTAAAGGTTTGGAAAAGAAAAAAGAGACCGCCTCGTTTCTTTTTC
TTCGTCGAAAAAGGCAATAAAAATTTTTATCACGTTTCTTTTTCTTGAAATTTTTTTTTTT
AGTTTTTTTCTCTTTCAGTGACCTCCATTGATATTTAAGTTAATAAACGGTCTTCAATTTC
TCAAGTTTCAGTTTCATTTTTCTTGTTCTATTACAACTTTTTTTACTTCTTGTTCATTAGAA
AGAAAGCATAGCAATCTAATCTAAGGGGCGGTGTTGACAATTAATCATCGGCATAGTA
TATCGGCATAGTATAATACGACAAGGTGAGGAACTAAACCATGGGTAAAAAGCCTGAA
CTCACCGCGACGTCTGTCGAGAAGTTTCTGATCGAAAAGTTCGACAGCGTCTCCGACCT
GATGCAGCTCTCGGAGGGCGAAGAATCTCGTGCTTTCAGCTTCGATGTAGGAGGGCGTG
GATATGTCCTGCGGGTAAATAGCTGCGCCGATGGTTTCTACAAAGATCGTTATGTTTATC
GGCACTTTGCATCGGCCGCGCTCCCGATTCCGGAAGTGCTTGACATTGGGGAATTCAGC
GAGAGCCTGACCTATTGCATCTCCCGCCGTGCACAGGGTGTCACGTTGCAAGACCTGCC
TGAAACCGAACTGCCCGCTGTTCTGCAGCCGGTCGCGGAGGCCATGGATGCGATCGCT
GCGGCCGATCTTAGCCAGACGAGCGGGTTCGGCCCATTCGGACCGCAAGGAATCGGTC
AATACACTACATGGCGTGATTTCATATGCGCGATTGCTGATCCCCATGTGTATCACTGGC
AAACTGTGATGGACGACACCGTCAGTGCGTCCGTCGCGCAGGCTCTCGATGAGCTGAT
GCTTTGGGCCGAGGACTGCCCCGAAGTCCGGCACCTCGTGCACGCGGATTTCGGCTCCA
ACAATGTCCTGACGGACAATGGCCGCATAACAGCGGTCATTGACTGGAGCGAGGCGAT
GTTCGGGGATTCCCAATACGAGGTCGCCAACATCTTCTTCTGGAGGCCGTGGTTGGCTT
GTATGGAGCAGCAGACGCGCTACTTCGAGCGGAGGCATCCGGAGCTTGCAGGATCGCC
GCGGCTCCGGGCGTATATGCTCCGCATTGGTCTTGACCAACTCTATCAGAGCTTGGTTGA
CGGCAATTTCGATGATGCAGCTTGGGCGCAGGGTCGATGCGACGCAATCGTCCGATCCG
GAGCCGGGACTGTCGGGCGTACACAAATCGCCCGCAGAAGCGCGGCCGTCTGGACCGA
TGGCTGTGTAGAAGTACTCGCCGATAGTGGAAACCGACGCCCCAGCACTCGTCCGAGG
GCAAAGGAATAATCAGTACTGACAATAAAAAGATTCTTGTTTTCAAGAACTTGTCATTT
GTATAGTTTTTTTATATTGTAGTTGTTCTATTTTAATCAAATGTTAGCGTGATTTATATTTT
TTTTCGCCTCGACATCATCTGCCCAGATGCGAAGTTAAGTGCGCAGAAAGTAATATCAT
GCGTCAATCGTATGTGAATGCTGGTCGCTATACTGCTGTCGATTCGATACTAACGCCGC
CATCCAGTGTCGAAAACGAGCTCGAATTCATCGATGATGTACCCAGCTTTTGTTCCCTTT
AGTGAGGGTTAATTCCGAGCTTGGCGTAATCATGGTCATAGCTGTTTCCTGTGTGAAATT
GTTATCCGCTCACAATTCCACACAACATACGAGCCGGAAGCATAAAGTGTAAAGCCTG
GGGTGCCTAATGAGTGAGCTAACTCACATTAATTGCGTTGCGCTCACTGCCCGCTTTCCA
GTCGGGAAACCTGTCGTGCCAGCTGCATTAATGAATCGGCCAACGCGCGGGGAGAGGC
GGTTTGCGTATTGGGCGCTCTTCCGCTTCCTCGCTCACTGACTCGCTGCGCTCGGTCGTTC
GGCTGCGGCGAGCGGTATCAGCTCACTCAAAGGCGGTAATACGGTTATCCACAGAATC
AGGGGATAACGCAGGAAAGAACATGTGAGCAAAAGGCCAGCAAAAGGCCAGGAACC
GTAAAAAGGCCGCGTTGCTGGCGTTTTTCCATAGGCTCCGCCCCCCTGACGAGCATCAC

AAAAATCGACGCTCAAGTCAGAGGTGGCGAAACCCGACAGGACTATAAAGATACCAG
GCGTTTCCCCCTGGAAGCTCCCTCGTGCGCTCTCCTGTTCCGACCCTGCCGCTTACCGGA
TACCTGTCCGCCTTTCTCCCTTCGGGAAGCGTGGCGCTTTCTCATAGCTCACGCTGTAGG
TATCTCAGTTCGGTGTAGGTCGTTCGCTCCAAGCTGGGCTGTGTGCACGAACCCCCCGTT
CAGCCCGACCGCTGCGCCTTATCCGGTAACTATCGTCTTGAGTCCAACCCGGTAAGACA
CGACTTATCGCCACTGGCAGCAGCCACTGGTAACAGGATTAGCAGAGCGAGGTATGTA
GGCGGTGCTACAGAGTTCTTGAAGTGGTGGCCTAACTACGGCTACACTAGAAGGACAG
TATTTGGTATCTGCGCTCTGCTGAAGCCAGTTACCTTCGGAAAAAGAGTTGGTAGCTCTT
GATCCGGCAAACAAACCACCGCTGGTAGCGGTGGTTTTTTTGTTTGCAAGCAGCAGATT
ACGCGCAGAAAAAAAGGATCTCAAGAAGATCCTTTGATCTTTTCTACGGGGTCTGACGC
TCAGTGGAACGAAAACTCACGTTAAGGGATTTTGGTCATGAGATTATCAAAAAGGATCT
TCACCTAGATCCTTTTAAATTAAAAATGAAGTTTTAAATCAATCTAAAGTATATATGAGT
AAACTTGGTCTGACAGTTACCAATGCTTAATCAGTGAGGCACCTATCTCAGCGATCTGT
CTATTTCGTTCATCCATAGTTGCCTGACTCCCCGTCGTGTAGATAACTACGATACGGGAG
GGCTTACCATCTGGCCCCAGTGCTGCAATGATACCGCGAGACCCACGCTCACCGGCTCC
AGATTTATCAGCAATAAACCAGCCAGCCGGAAGGGCCGAGCGCAGAAGTGGTCCTGCA
ACTTTATCCGCCTCCATCCAGTCTATTAATTGTTGCCGGGAAGCTAGAGTAAGTAGTTCG
CCAGTTAATAGTTTGCGCAACGTTGTTGCCATTGCTACAGGCATCGTGGTGTCACGCTCG
TCGTTTGGTATGGCTTCATTCAGCTCCGGTTCCCAACGATCAAGGCGAGTTACATGATCC
CCCATGTTGTGCAAAAAAGCGGTTAGCTCCTTCGGTCCTCCGATCGTTGTCAGAAGTAA
GTTGGCCGCAGTGTTATCACTCATGGTTATGGCAGCACTGCATAATTCTCTTACTGTCAT
GCCATCCGTAAGATGCTTTTCTGTGACTGGTGAGTACTCAACCAAGTCATTCTGAGAAT
AGTGTATGCGGCGACCGAGTTGCTCTTGCCCGGCGTCAATACGGGATAATACCGCGCCA
CATAGCAGAACTTTAAAAGTGCTCATCATTGGAAAACGTTCTTCGGGGCGAAAACTCTC
AAGGATCTTACCGCTGTTGAGATCCAGTTCGATGTAACCCACTCGTGCACCCAACTGAT
CTTCAGCATCTTTTACTTTCACCAGCGTTTCTGGGTGAGCAAAAACAGGAAGGCAAAAT
GCCGCAAAAAAGGGAATAAGGGCGACACGGAAATGTTGAATACTCATACTCTTCCTTT
TTCAATATTATTGAAGCATTTATCAGGGTTATTGTCTCATGAGCGGATACATATTTGAAT
GTATTTAGAAAAATAAACAAATAGGGGTTCCGCGCACATTTCCCCGAAAAGTGCCACC
TGGGAAATTGTAAACGTTAATATTTTGTTAAAATTCGCGTTAAATTTTTGTTAAATCAGC
TCATTTTTTAACCAATAGGCCGAAATCGGCAAAATCCCTTATAAATCAAAAGAATAGAC
CGAGATAGGGTTGAGTGTTGTTCCAGTTTGGAACAAGAGTCCACTATTAAAGAACGTGG
ACTCCAACGTCAAAGGGCGAAAAACCGTCTATCAGGGCGATGGCCCACTACGTGAACC
ATCACCCTAATCAAGTTTTTTGGGGTCGAGGTGCCGTAAAGCACTAAATCGGAACCCTA
AAGGGAGCCCCCGATTTAGAGCTTGACGGGGAAAGCCGGCGAACGTGGCGAGAAAGG
AAGGGAAGAAAGCGAAAGGAGCGGGCGCTAGGGCGCTGGCAAGTGTAGCGGTCACGC
TGCGCGTAACCACCACACCCGCCGCGCTTAATGCGCCGCTACAGGGCGCGTCGCGCCA
TTCGCCATTCAGGCTGCGCAACTGTTGGGAAGGGCGATCGGTGCGGGCCTCTTCGCTAT
TACGCCAGCTGGCGAAAGGGGGATGTGCTGCAAGGCGATTAAGTTGGGTAACGCCAGG
GTTTTCCCAGTCACGACGTTGTAAAACGACGGCCAGTGAATTGTAATACGACTCACTAT
AGGGCGAATTGGAGCTCCACCGCGGTGGCGGCCGCCACGGCGATATCGGATCTCGACG
CGGAGAACGATCTCCTCGAGCTGCTCGCGGATCAGCTTGTGGCCCGGTAATGGAACCA
GGCCGACGCGACGCTCCTTGCGGACCACGGTGGCTGGCGAGCCCAGTTTGTGAACGAG
GTCGTTTAGAACGTCCTGCGCAAAGTCCAGTGTCAGATGAATGTCCTCCTCGGACCAAT
TCAGCATGTTCTCGAGCAGCCATCTGTCTTTGGAGTAGAAGCGTAATCTCTGCTCCTCGT

TACTGTACCGGAAGAGGTAGTTTGCCTCGCCGCCCATAATGAACAGGTTCTCTTTCTGGT
GGCCTGTGAGCAGCGGGGACGTCTGGACGGCGTCGATGAGGCCCTTGAGGCGCTCGTA
GTACTTGTTCCGTCGCTGTAGCCGGCCGCGGTGACGATACCCACATAGAGGTCCTTGGC
CATTAGTTTGATGAGGTGGGGCAGGATGGGCGACTCGGCATCGAAATTTTTGCCGTCGT
CGTACAGTGTGATGTCACCATCGAATGTAATGAGCTGCAGCTTGCGATCTCGGATGGTT
TTGGAATGGAAGAACCGCGACATCTCCAACAGCTGGGCCGTGTTGAGAATGAGCCGGA
CGTCGTTGAACGAGGGGGCCACAAGCCGGCGTTTGCTGATGGCGCGGCGCTCGTCCTCG
ATGTAGAAGGCCTTTTCCAGAGGCAGTCTCGTGAAGAAGCTGCCAACGCTCGGAACCA
GCTGCACGAGCCGAGACAATTCGGGGGTGCCGGCTTTGGTCATTTCAATGTTGTCGTCG
ATGAGGAGTTCGAGGTCGTGGAAGATTTCCGCGTAGCGGCGTTTTGCCTCAGAGTTTAC
CATGAGGTCGTCCACTGCAGAGATGCCGTTGCTCTTCACCGCGTACAGGACGAACGGC
GTGGCCAGCAGGCCCTTGATCCATTCTATGAGGCCATCTCGACGGTGTTCCTTGAGTGC
GTACTCCACTCTGTAGCGACTGGACATCTCGAGACTGGGCTTGCTGTGCTGGATGCACC
AATTAATTGTTGCCGCATGCATCCTTGCACCGCAAGTTTTTAAAACCCACTCGCTTTAGC
CGTCGCGTAAAACTTGTGAATCTGGCAACTGAGGGGGTTCTGCAGCCGCAACCGAACTT
TTCGCTTCGAGGACGCAGCTGGATGGTGTCATGTGAGGCTCTGTTTGCTGGCGTAGCCTA
CAACGTGACCTTGCCTAACCGGACGGCGCTACCCACTGCTGTCTGTGCCTGCTACCAGA
AAATCACCAGAGCAGCAGAGGGCCGATGTGGCAACTGGTGGGGTGTCGGACAGGCTGT
TTCTCCACAGTGCAAATGCGGGTGAACCGGCCAGAAAGTAAATTCTTATGCTACCGTGC
AGTGACTCCGACATCCCCAGTTTTTGCCCTACTTGATCACAGATGGGGTCAGCGCTGCC
GCTAAGTGTACCCAACCGTCCCCACACGGTCCATCTATAAATACTGCTGCCAGTGCACG
GTGGTGACATCAATCTAAAGTACAAAAACATGGCGGTTGGGATTTACATCAGAGTCTC
AACCCAAGAGCAGGCGAGTGAAGGGCACAGTATTGAAAGCCAAAAAAAGAAACTGGC
GTCTTATTGTGAAATCCAAGGATGGGATGACTACAGGTTCTACATCGAAGAGGGCATAT
CCGGGAAAAACACAAATAGACCGAAGCTTAAGCTATTAATGGAACATATCGAAAAGG
GAAAAATTAACATTTTATTGGTCTACAGGCTGGATAGGTTGACTAGGTCTGTGATCGATT
TACATAAGCTATTAAACTTTTTACAGGAACATGGGTGCGCGTTTAAATCTGCTACAGAA
ACTTACGACACAACTACTGCAAACGGAAGGATGAGTATGGGTATAGTGAGTCTTCTAG
CCCAATGGGAGACAGAAAATATGAGTGAGCGTATTAAACTAAACCTGGAACACAAAG
TCTTGGTTGAGGGGGAAAGAGTAGGGGCGATTCCCTATGGATTCGACTTGTCAGATGAT
GAAAAGCTTGTGAAGAATGAAAAGTCTGCAATTTTATTGGACATGGTCGAAAGGGTGG
AGAACGGCTGGTCCGTCAATAGGATCGTCAACTATCTTAATTTAACTAACAATGATCGT
AACTGGTCACCTAATGGGGTGCTACGTTTGTTAAGGAACCCTGCACTATATGGCGCTAC
AAGGTGGAATGATAAAATCGCAGAGAACACACACGAGGGTATAATTAGCAAGGAACG
TTTCAACCGTCTGCAGCAAATACTTGCAGACCGTAGCATCCATCACAGACGTGATGTGA
AAGGAACATACATATTTCAAGGAGTTTTGAGATGTCCGGTTTGTGATCAGACGCTGTCC
GTTAATAGGTTTATTAAGAAGCGTAAGGATGGAACAGAGTACTGTGGTGTCCTTTATAG
GTGTCAGCCATGTATTAAGCAAAACAAGTACAATTTAGCTATCGGCGAAGCTAGGTTCC
TGAAGGCCCTTAACGAGTACATGTCTACGGTGGAATTCCAGACAGTTGAAGACGAGGT
GATACCCAAGAAAAGTGAGAGAGAAATGTTGGAATCTCAGCTGCAACAGATCGCAAG
AAAGAGGGAGAAATACCAAAAGGCATGGGCGAGCGATTTAATGTCCGATGATGAATTT
GAGAAACTTATGGTCGAGACCCGTGAAACTTATGACGAATGCAAGCAAAAACTGGAG
AGTTGCGAGGACCCTATTAAGATCGACGAGACATATTTGAAGGAAATAGTTTACATGTT
TCATCAAACATTCAATGATTTAGAGTCCGAGAAGCAAAAGGAGTTTATATCAAAATTTA
TAAGGACTATCCGTTACACCGTCAAAGAGCAGCAACCTATCAGACCTGATAAGTCTAA

GACAGGTAAGGGTAAACAGAAAGTGATAATTACGGAAGTGGAGTTTTACCAGTAAAA
GCTTGCATGCCTGCAGGTCGACTCTAGAGGATCGATCCCCGGGCCTGGACATCCAGCCT
TCCTACGCCATGACCACCTCCGAGGCTAAGAGGGCCGTGCACAAGGAGACCAAGGAC
AAAACCTCGAGACTTGCCTTTGAAGGCTCTTGTTGCGGTAAATAAGTATATAGGACACG
ACAATCTAGTAATCTCCACTATTGACGAGCTCGTCGAACTGCGAAAATAGGTTTTCCAT
CTGGTCTGTAGGCATCAGCCCGGCGTCATCCTCCTGCGCAGGAGCAGCGGGCTCAGGG
CCGGCCTGGGCGGGCTGATCCAGAAAGTCGAGGTTCA

Figure S1. Confirmation of integration of both genes into the EMB101 strain by colony PCR of 12 colonies selected after transformation and plating on selective medium. The upper gel shows amplification of the *hasB* gene (1505 bp) using the primer pair hasB_F and hasB_R. The bottom gel shows amplification of the *hasAp* gene (2966 bp) using the primer pair hasAp_F and hasAp_R. The agarose content was 1% and the ladders used were 1kB Ladder Plus M1191/M1192 (M1; Sinapse Inc) 1 kb Ladder K9 (M2; Kasvi). The *O. polymorpha* NCYC495 *yku80* was used as a negative control (C-) for the PCR reactions. The pHIPH4_*hasB* and pHIPZ18_*hasAp* plasmids were used as positive controls (C+) for the PCR reactions of *hasB* and *hasAp*, respectively.

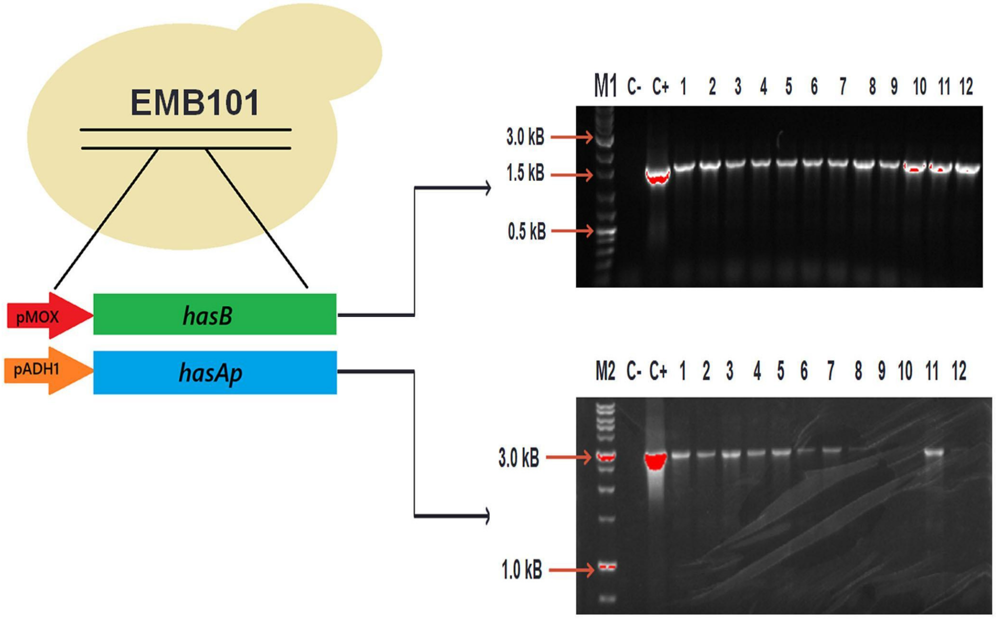

Figure S2. Colony PCRs to verify the *hasAp* (2966 bp) and *hasB* (1505 bp) genes stability in the genome of EMB101 strain after three successive passages on YPD supplemented with zeocin and hygromycin. Five colonies were selected after the transformation and plating on YPD plate containing both antibiotics for the verification of *hasB* and *hasAp* stability on the genome of *O. polymorpha* NCYC495 *yku80*. All PCR reactions for *hasB* gene were performed using the primer pair hasB_F and hasB_R while the PCRs reactions for *hasAp* gene using the primer pair hasAp_F and hasAp_R. In all PCRs reactions performed, the *O. polymorpha* NCYC495 *yku80* was used as a negative control (C-) and the pHIPH4_*hasB* and pHIPZ18_*hasAp* plasmids were used as positive controls (C+) for the of *hasB* and *hasAp*, respectively. The fragments amplified at the height of 1.5 kb in the upper gels correspond to the *hasB* gene and the fragments in the height of 3.0 kb at the bottom gels correspond to the *hasAp* gene. The black arrows indicate a passaging to another YPD plate supplemented with zeocin and hygromycin. The agarose content was 1% and the ladder used was 1 kb Plus DNA Ladder (M1; Invitrogen). **(A)** Colony PCR for the *hasAp* gene of the first passaging on selective medium, **(B)** Colony PCR for the hasB (upper gel) and hasP (bottom gel) genes of the second passaging on YPD plate with both antibiotics. **(C)** Colony PCR for the *hasB* (upper gel) and *hasAp* (bottom gel) genes of the third passaging on a YPD plate with both antibiotics.

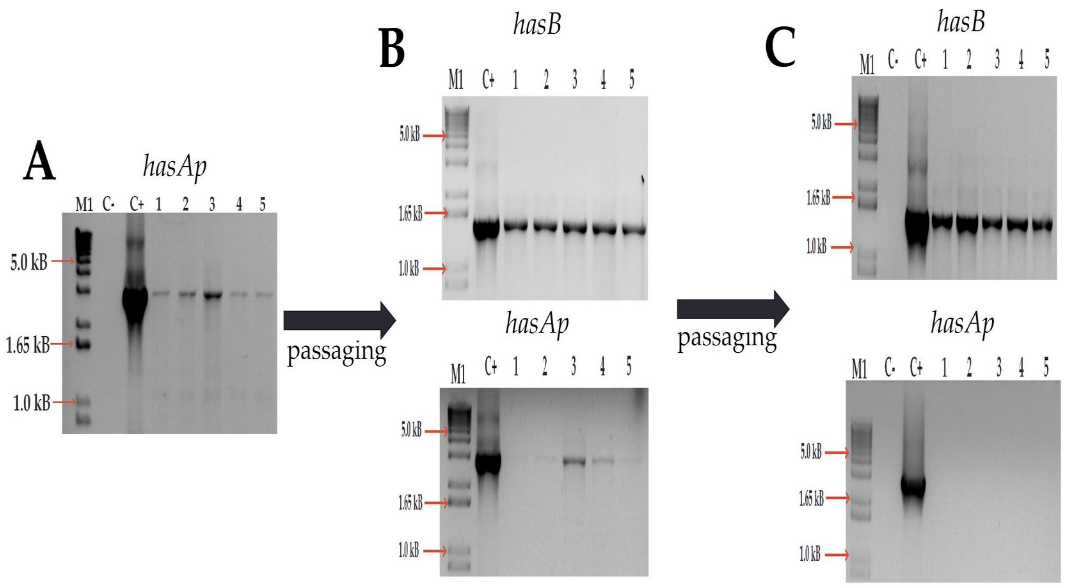

Figure S3. Confirmation of integration of both genes into the EMB102 strain by colony PCR of 12 colonies selected after the transformation and plating on selective medium. The upper gel shows amplification of the *hasB* gene (1.6 kB) using the primer pair hasB_F and hasB_R. The bottom gel shows amplification of the *hasAp* gene (2.9 kB) using the primer pair hasAp_F and hasAp_R. The agarose content was 1% and the ladders used were 1kB Ladder Plus M1191/M1192 (M1; Sinapse Inc) 1 kb Ladder K9 (M2; Kasvi). The *O. polymorpha* NCYC495 *yku80* was used as a negative control (C-) for the PCR reactions. The pHIPH4_*hasB* and pHIPZ7_*hasAp* plasmids were utilized as positive controls (C+) for the PCR reactions of *hasB* and *hasAp*, respectively.

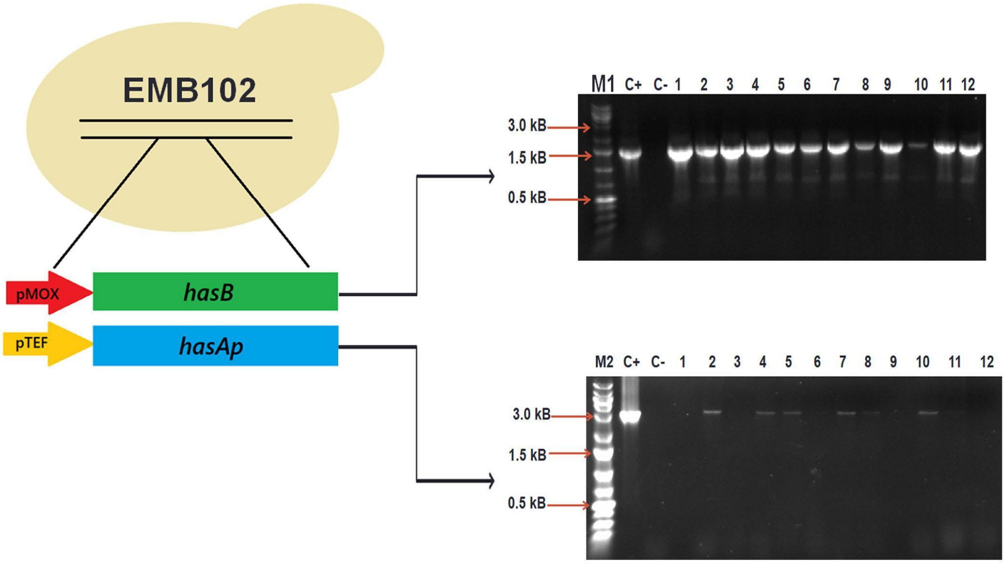

Figure S4. Scheme representing the genetic switch constructed to control the expression of both *hasB* and *hasAp* by a serine integrase. (A) The gene encoding the Int13 codon-optimized for *S. cerevisiae* is regulated by the promoter pAOX which is inducible by methanol (details in Figure S14). Thus, the addition of methanol leads to the production of Int13 that recognizes the sites *attB* and *attP* flanking both *has* genes synthesized in reverse complement orientation. The action of Int13 causes a rotation of 180° in both genes resulting in two different flanking sequences named *attL* and *attR*. In the final, both genes are in ORF with promoter and terminator and can be properly transcripted. The correct gene orientation as well the formation of *attL* sequence could be evaluated by PCR using the pair of primer attB_hasB_F and attP_hasB_R for *hasB* (resulting fragment of 493 bp) and attB_hasAp_F and attP_hasAp_R for *hasAp* (resulting fragment 827 bp). When the genes are in the initial orientation, the primer annealing fails and no amplification occurs. The black arrows represent the primer orientation. (B) Confirmation by PCR of the both genes rotation in the EMB103 strain after methanol induction. For all PCRs reactions, the genomic DNA was utilized as the template and for the extraction the phenol/chloroform method was applied according to [2]. The gel in the left shows the fragment (827 bp) containing the *attL* sequence and the *hasAp* flipped and in the right the fragment for *hasB* (493 bp).The *O. polymorpha* NCYC495 *yku80* was used as a negative control (C-) for the PCR reactions. The pHIPZ18_*hasAp* plasmid which contains the *hasAp* in ORF was utilized as positive controls (C+) for the PCR reactions of *hasAp*. The plasmid PKLAC2-BP constructed previously for our group (REF) was utilized as the control positive for the PCR reaction of *hasB* once all plasmids constructed in this work has the *hasB* gene controlled by endogenous promoters of *O. polymorpha* instead of pGDP promoter from *S. cerevisiae*.

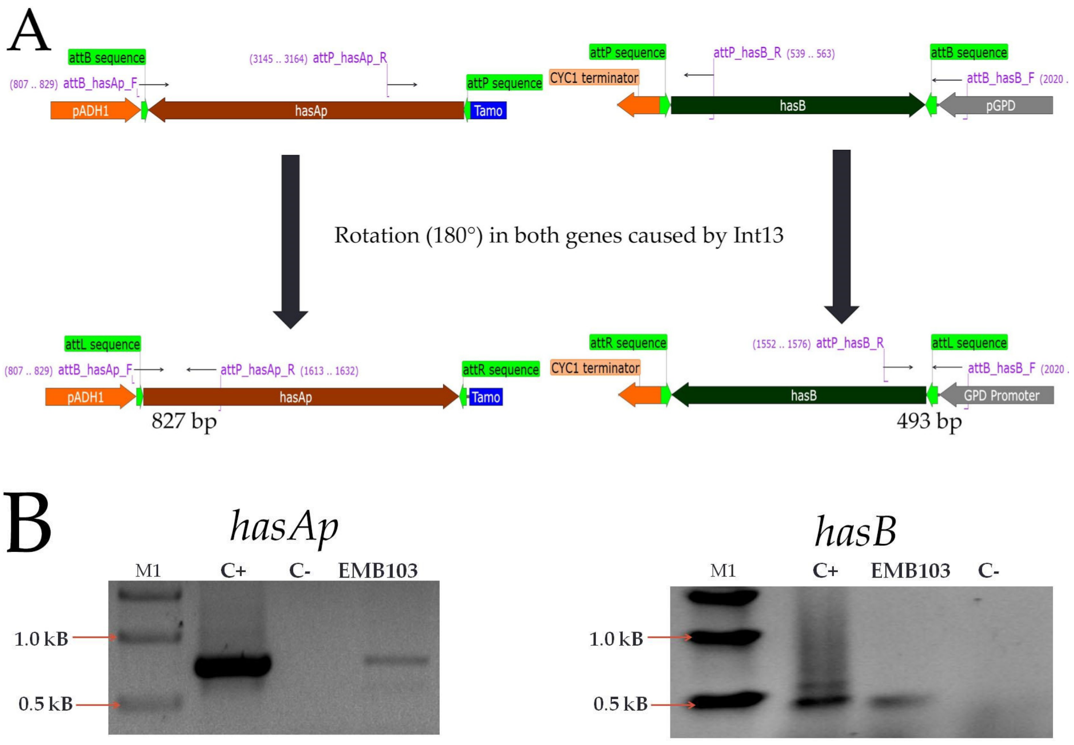

Figure S5. Confirmation of integration of both genes into the EMB104 strain by colony PCR of 12 colonies selected after the transformation and plating on selective medium. The upper gel shows amplification of the *hasB* gene (1.6 kB) using the pair of primers hasB_F and hasB_R. The bottom gel shows amplification of the *hasAs* gene between the pAOX (pMOX) promoter and the *AMO* terminator (1.5 kB) using the pair of primers AOX-Integration_F and AOX-Integration_R. The agarose content was 1% and the ladder used was 1kB Ladder M1181/M1182 (M1; Sinapse Inc). The *O. polymorpha* NCYC495 *yku80* was used as a negative control (C-) for the PCR reactions. The pHIPZ18_*hasB* and pHIPH4_*hasAs* plasmids were used as positive controls (C+) for the PCR reactions of *hasB* and *hasAs*, respectively.

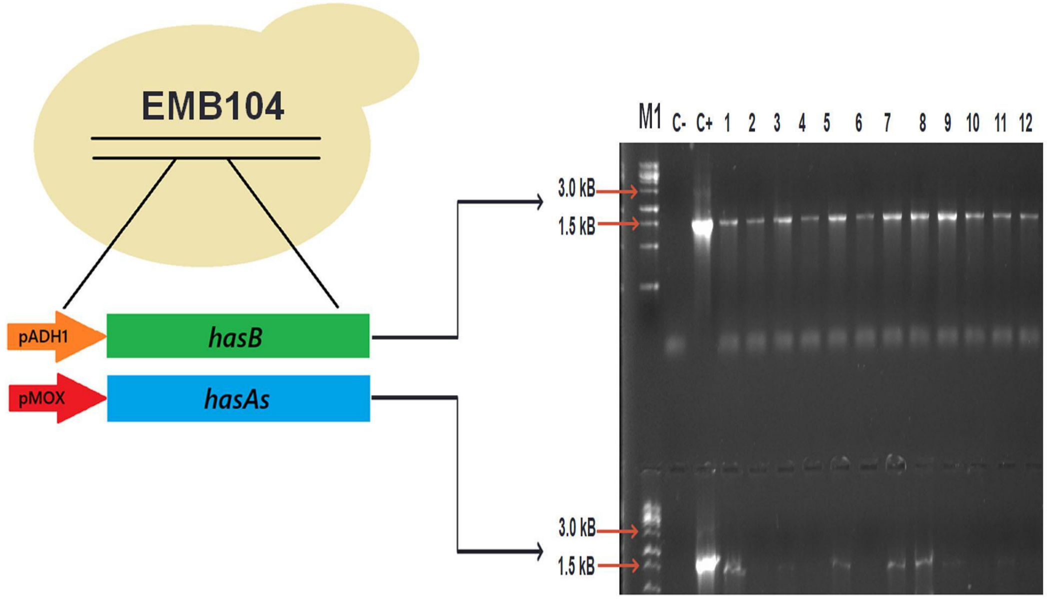

Figure S6. Map of the synthetic plasmid pBSK_*hasB* harbouring the *hasB* gene from *Xenopus laevis*. Oligonucleotide primers for amplification of *hasB* (HasB_F and HasB_R) are shown on their annealing sites.

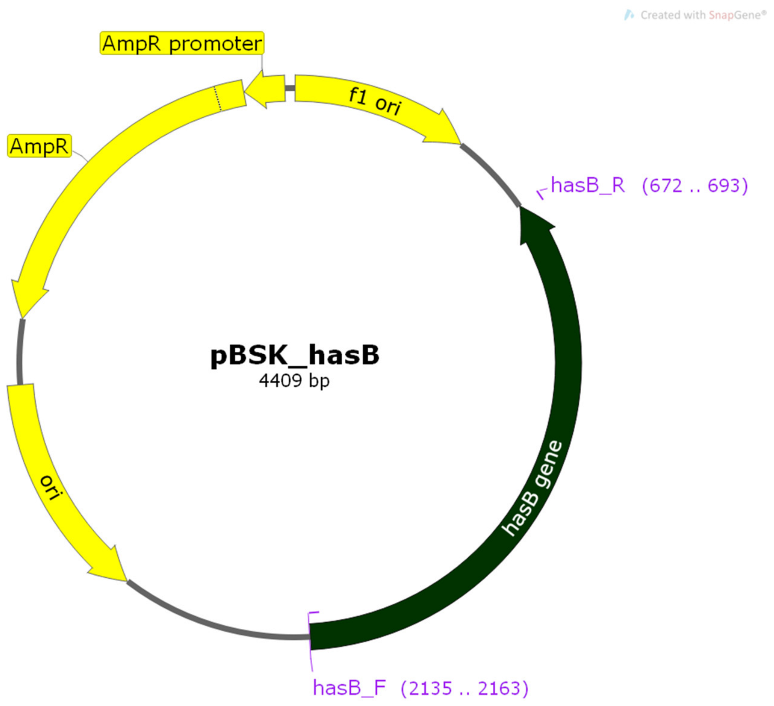

Figure S7. Map of the synthetic plasmid pBSK_*hasAp* harbouring the *hasA* gene from *Pasteurella multocida*. Primers for amplification of *hasAp* (HasAp_F and HasAp_R) are shown on their annealing sites.

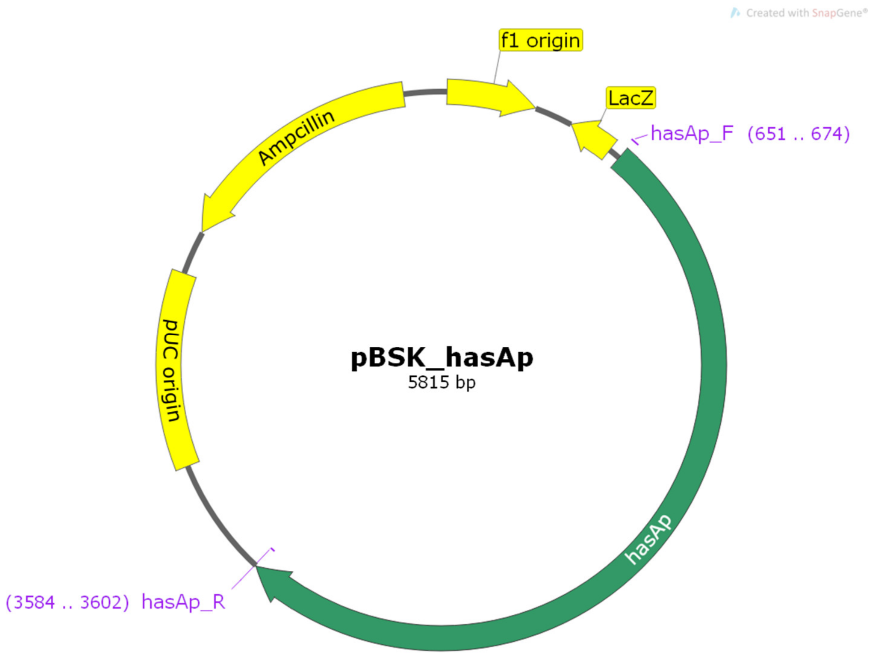

Figure S8. Map of the synthetic plasmid pBSK_*hasAs* harbouring the *hasA* gene from *Streptococcus zooepidemicus*. Restriction sites for HindIII and XbaI are shown.

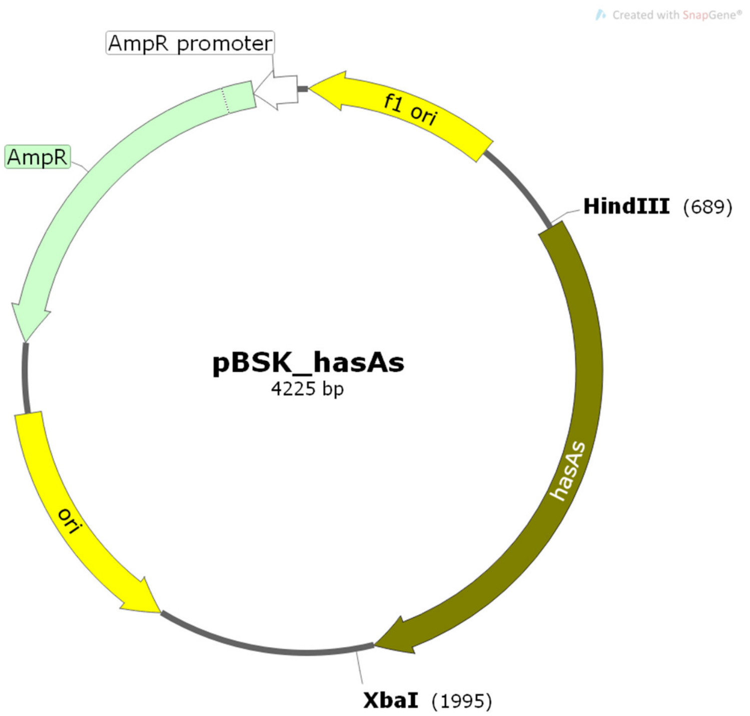

Figure S9. Map of the pGEM_*hasB* plasmid harbouring the *hasB* gene from *X. laevis*. Restriction sites for HindIII and SalI are shown.

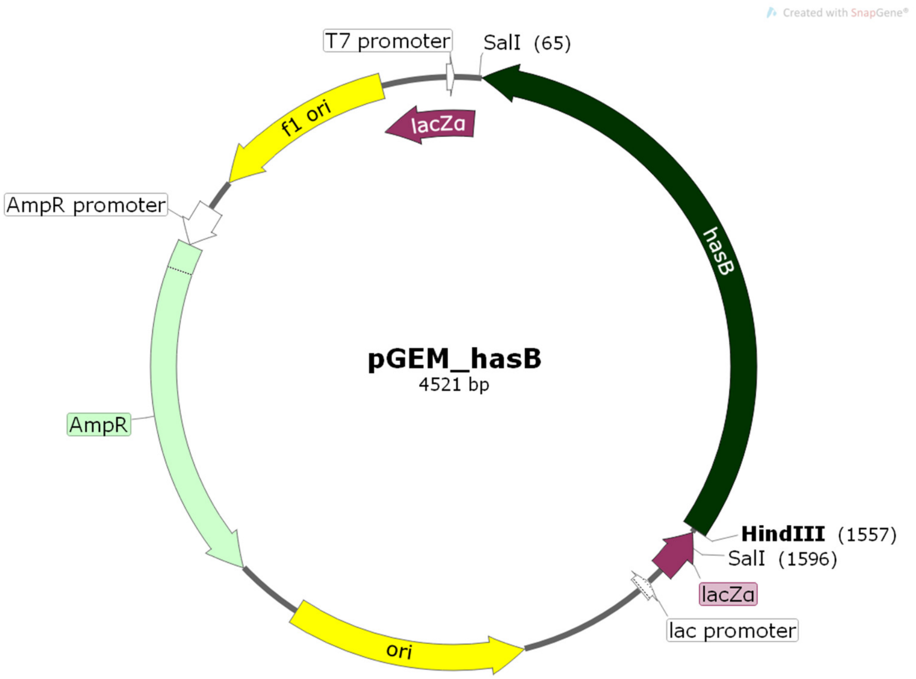

Figure S10. Map of the pHIPZ7_*hasAp* plasmid bearing the *hasAp* gene from *P. multocida*. Primers for amplification of *hasAp* (HasAp_F and HasAp_R) are shown on their annealing sites. Restriction sites for HindIII and XhoI are shown.

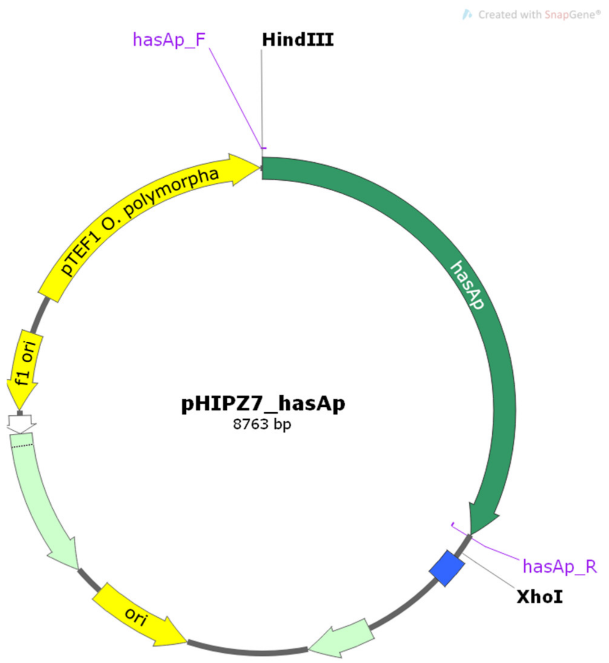

Figure S11. Map of the pHIPZ18_*hasAp* plasmid harbouring the *hasA* gene from *P. multocida*. Primers for amplification of *hasAp* (HasAp_F and HasAp_R) are shown on their annealing sites. Restriction sites for HindIII and XhoI are shown.

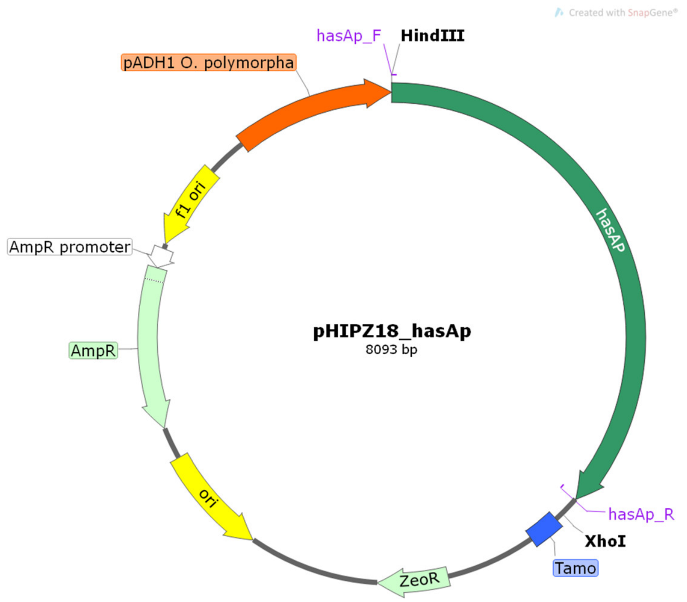

Figure S12. Map of the pHIPZ18_*hasB* plasmid bearing the *hasB* gene from *X. laevis*. Primers for amplification of *hasB* are shown on their annealing sites. Restriction sites for HindIII and SalI are shown.

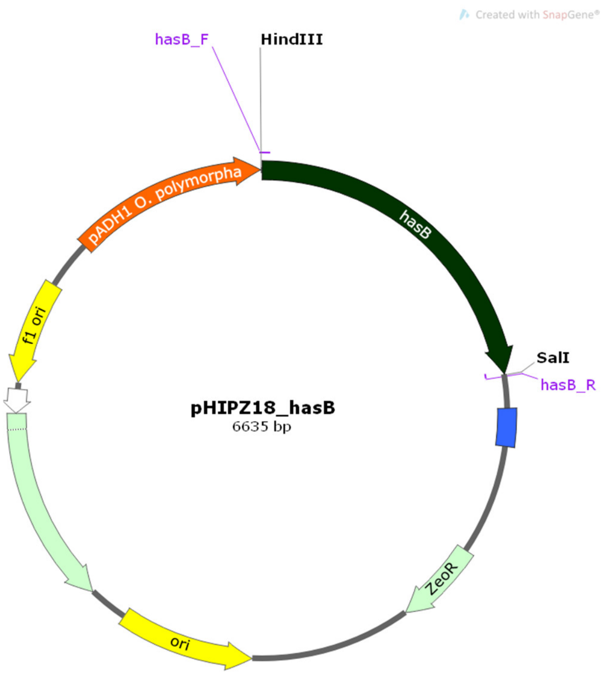

Figure S13. Map of the pHIPH4_*hasB* plasmid harbouring the *hasB* gene from *X. laevis*. Primers for amplification of *hasB* (HasB_F and HasB_R) are shown on their annealing sites. Restriction sites for HindIII and SalI are shown.

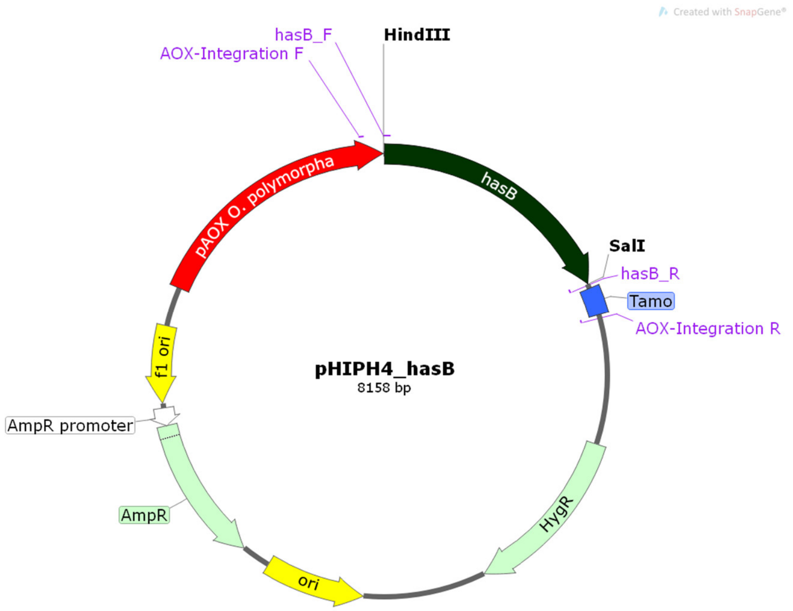

Figure S14. Map of the pHIPH4_*hasAs* plasmid harbouring the *hasA* gene from *S. zooepidemicus*. Primers for amplification of *hasA* (AOX-Integration F and AOX-Integration R) are shown on their annealing sites. Restriction sites for HindIII and XbaI are shown.

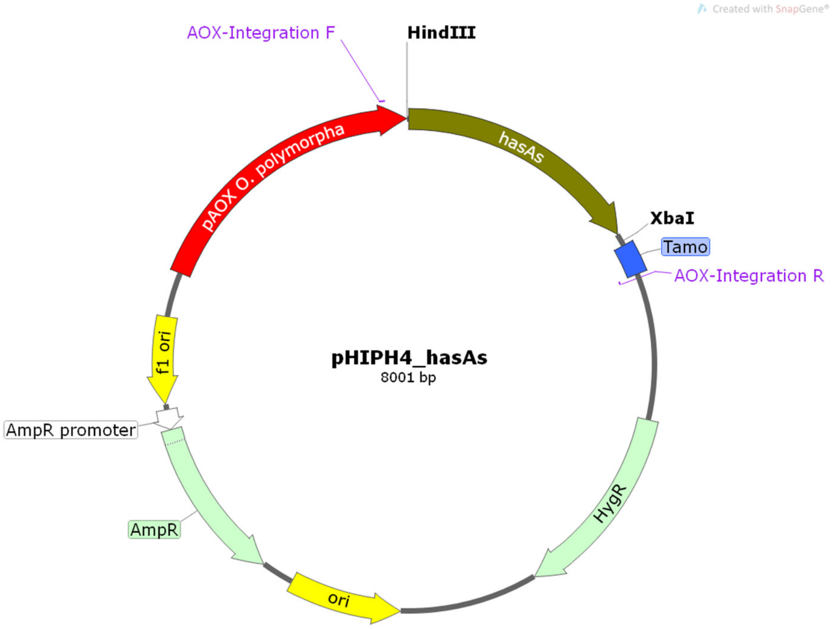

Figure S15. Map of the pHIPH4_*ScSInt13* plasmid harbouring the *hasB* gene from *X. laevis* and the *hasA* gene from *P. multocida*. Primers for amplification of *hasB* (HasB Forward and HasB Reverse) and *hasAp* (HasAp Forward and HasAp Reverse) are shown on their annealing sites.

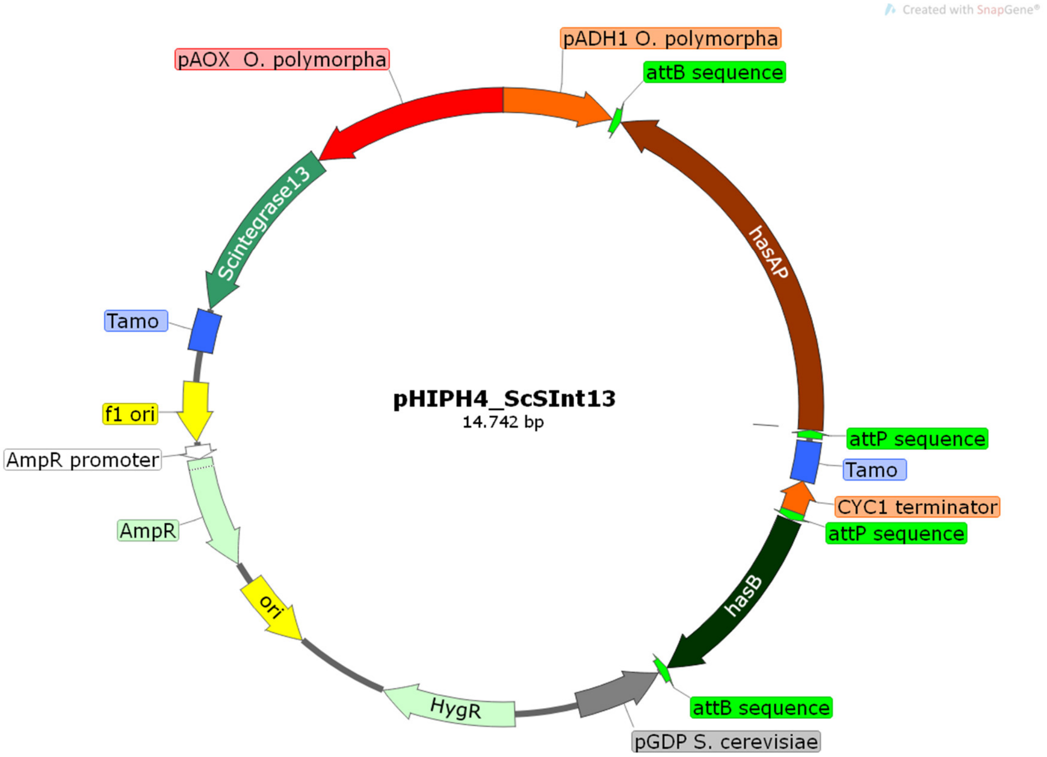

Figure S16. Standard curve obtained by the carbazole method for quantifying HA.

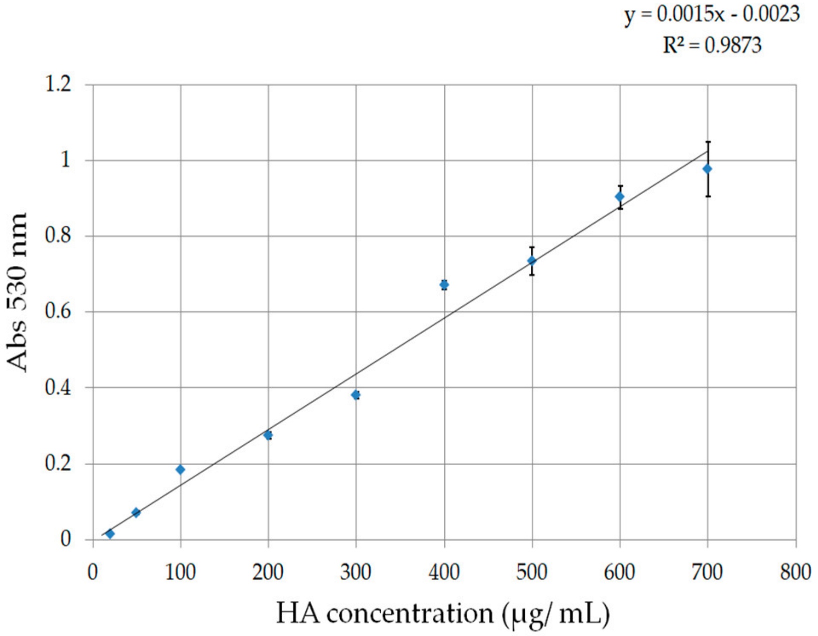

Figure S17. Carbazole assay plate picture. (A) The 96-well plate of the carbazole assay performed to quantify the HA in the supernatants after the 48 h of cultivation. (B) The plate design indicating the position of each sample in (A). I and II indicated the biological replicate. All quantifications were performed in technical replicate. The empty wells are represented with a line."

A

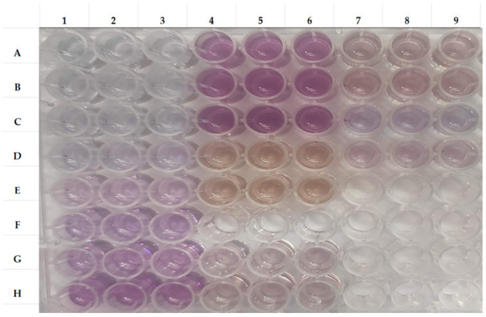

B

	1	2	3	4	5	6	7	8	9
A	Blank	Blank	Blank	500 µg/mL	500 µg/mL	500 µg/mL	EMB103 I	EMB103 I	EMB103 I
B	10 µg/mL	10 µg/mL	10 µg/mL	600 µg/mL	600 µg/mL	600 µg/mL	EMB103 I	EMB103 I	EMB103 I
C	20 µg/mL	20 µg/mL	20 µg/mL	700 µg/mL	700 µg/mL	700 µg/mL	EMB104 I	EMB104 I	EMB104 I
D	50 µg/mL	50 µg/mL	50 µg/mL	WT I	WT I	WT I	EMB104 II	EMB104 II	EMB104 II
E	100 µg/mL	100 µg/mL	100 µg/mL	WT II	WT II	WT II	-	-	-
F	200 µg/mL	200 µg/mL	200 µg/mL	-	-	-	-	-	-
G	300 µg/mL	300 µg/mL	300 µg/mL	EMB102 I	EMB102 I	EMB102 I	-	-	-
H	400 µg/mL	400 µg/mL	400 µg/mL	EMB102 II	EMB102 II	EMB102 II	-	-	-

References

1. van der Klei, I.J. The Hansenula polymorpha expression system Available online: https://www.rug.nl/research/molecular-cell-biology/research/the-hansenula-polymorpha-expression-system (accessed on May 19, 2020).

2. Sambrook, J.; Russell, D.W. *Molecular Cloning - A Laboratory Manual*; 3rd ed.; Cold Spring Harbor Laboratory Press, Cold Spring Harbor: New York, 2001;

3. V. Gomes, A.M.; C. M. Netto, J.H.; Carvalho, L.S.; Parachin, N.S. Heterologous Hyaluronic Acid Production in Kluyveromyces lactis. Microorganisms 2019, 7, 294, doi:10.3390/microorganisms7090294.
